# Shake a Tail Feather: The Evolution of the Theropod Tail into a Stiff Aerodynamic Surface

**DOI:** 10.1371/journal.pone.0063115

**Published:** 2013-05-15

**Authors:** Michael Pittman, Stephen M. Gatesy, Paul Upchurch, Anjali Goswami, John R. Hutchinson

**Affiliations:** 1 Department of Earth Sciences, University College London, London, United Kingdom; 2 Department of Ecology and Evolutionary Biology, Brown University, Providence, Rhode Island, United States of America; 3 Department of Genetics, Evolution, and Environment, University College London, London, United Kingdom; 4 Structure & Motion Laboratory, Department of Comparative Biomedical Sciences, The Royal Veterinary College, Hatfield, United Kingdom; Ludwig-Maximilians-Universität München, Germany

## Abstract

Theropod dinosaurs show striking morphological and functional tail variation; e.g., a long, robust, basal theropod tail used for counterbalance, or a short, modern avian tail used as an aerodynamic surface. We used a quantitative morphological and functional analysis to reconstruct intervertebral joint stiffness in the tail along the theropod lineage to extant birds. This provides new details of the tail’s morphological transformation, and for the first time quantitatively evaluates its biomechanical consequences. We observe that both dorsoventral and lateral joint stiffness decreased along the non-avian theropod lineage (between nodes Theropoda and Paraves). Our results show how the tail structure of non-avian theropods was mechanically appropriate for holding itself up against gravity and maintaining passive balance. However, as dorsoventral and lateral joint stiffness decreased, the tail may have become more effective for dynamically maintaining balance. This supports our hypothesis of a reduction of dorsoventral and lateral joint stiffness in shorter tails. Along the avian theropod lineage (Avialae to crown group birds), dorsoventral and lateral joint stiffness increased overall, which appears to contradict our null expectation. We infer that this departure in joint stiffness is specific to the tail’s aerodynamic role and the functional constraints imposed by it. Increased dorsoventral and lateral joint stiffness may have facilitated a gradually improved capacity to lift, depress, and swing the tail. The associated morphological changes should have resulted in a tail capable of producing larger muscular forces to utilise larger lift forces in flight. Improved joint mobility in neornithine birds potentially permitted an increase in the range of lift force vector orientations, which might have improved flight proficiency and manoeuvrability. The tail morphology of modern birds with tail fanning capabilities originated in early ornithuromorph birds. Hence, these capabilities should have been present in the early Cretaceous, with incipient tail-fanning capacity in the earliest pygostylian birds.

## Introduction

The tails of theropods (bipedal carnivorous dinosaurs) underwent dramatic anatomical changes along the line of descent to modern birds [1], [2], [3], [4], [5]. Ancestrally, *Carnotaurus* and more basal forms had long, massive tails that were more similar to the tail of a crocodile than to the tail of a bird [6]. Theropod tails generally have two regions. Before the ‘transition point’ the caudal vertebrae have neural spines that are dorsoventrally tall and chevrons that are dorsoventrally deep, as well as wide spans between the tips of each vertebra’s transverse processes. After the transition point these features are greatly reduced or become absent. This transition is not actually a ‘point’ *per se* because the changes in the tail features are variable, unsynchronised and occur over several caudal vertebrae [3], [7]. In contrast, extant birds have short, light tails with caudal vertebrae that do not cross a transition point, but the tip of their tails are co-ossified (pygostyle) and support a tail fan [2], [3], [8].

**Figure 1:**
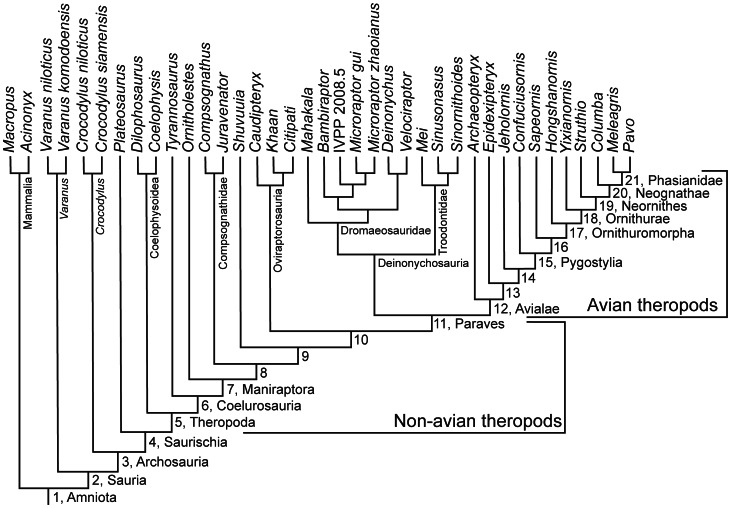
Phylogeny used for data mapping. The composite theropod evolutionary tree used in this study was compiled for non-avian coelurosaurs and for birds from [47], [57], [58], [98], [99], [100] with outgroups from [101]. The names and numbers of the nodes along the theropod lineage between Theropoda and Phasianidae refer to those used in the text.

Basal theropods had a large *caudofemoralis longus* (CFL) muscle that retracted the hind limb via its attachment point on the fourth trochanter of the femur [1], [9]. The position of the last transverse process approximates the distalmost extent of this muscle along the tail. Such a large muscle probably restricted the mobility of the tail base, making the tail behave as a passive stabiliser during walking and running, as in *Alligator* [1], [10], [11]. In coelurosaurs (Fig. 1), the shorter, narrower and lighter tail [3] with a smaller CFL would make a lighter animal if tail functions could be accomplished in other ways [1]. Coelurosaurian tails were probably more dynamic during stabilisation. This might have been used to improve manoeuvring, e.g., in response to faster prey, as a shorter tail should have reduced the animal’s rotational inertia (RI), and thus improved their ability to turn in yaw [10]. However, larger theropods appear to have partly overcome the handicap of their large tail by placing additional weight close to their centre of mass, which reduced the RI expected for their size [12]. But how did coelurosaurs run quickly with a smaller CFL than more basal theropods? Coelurosaurs had an incipient knee-based mechanism of hind limb retraction powered by muscles originating from the pelvis (as seen in living birds), potentially compensating for the smaller CFL [1], [4]. More generally, theropods have been speculated to have lifted their tails to reduce their RI [10], [13], [14], like some extant lizards do [10], as well as for pitch control during jumping; e.g., during aerial attacks on prey [15]. The latter is suggested by a computer model of the dromaeosaurid *Velociraptor* and by jumping experiments performed with a biomechanical robot as well as with living lizards [16], [17]. However, it remains unclear how flexible different theropod tails were, and thus how feasible such behaviours were or how they evolved.

The evolutionary reduction in theropod tail length culminated in the CFL muscle being very small or absent in extant birds. This reduction of the CFL implies that the tail and hind limb became functionally ‘decoupled’, enabling the tail to perform its aerodynamic functions more freely [18]. The evolution of theropod tail function has attracted some attention, but our knowledge of *in vivo* mechanics and control comes almost entirely from the tails of extant birds [2], [8], [19], [20], [21], [22], [23]. What were the intermediate stages during the morphological transformation of plesiomorphically large, muscular tails into short, feathered, aerodynamic modern avian tails, and what were the biomechanical consequences of such a major reorganization?

Basic properties such as length, diameter, taper, mass, centre of mass, and aerodynamic feathering are clearly important components of tail design. For example, tail length shows significant inter- and intraspecific (including ontogenetic) variability in many amniotes; e.g., squamates, mammals and non-avian and avian dinosaurs [24], [25], [26], [27], [28], [29], [30]; which might impact tail function. Herein, we focus on the geometric proportions of vertebrae that affect the mechanical behaviour of intervertebral joints. Each joint’s ability to rotate influences both the tail’s range of motion (mobility) and its resistance to motion. In the absence of muscular forces acting on the tail, the resistance or force needed to deflect a joint through a given arc; e.g., one radian; is its passive; i.e. osseoligamentous; intervertebral joint stiffness [31].

**Figure 2:**
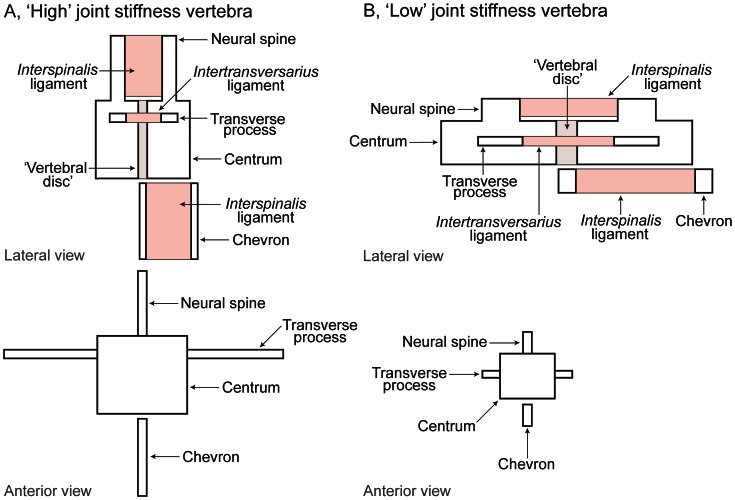
Hypothetical vertebral morphologies associated with ‘high’ and ‘low’ joint stiffness properties and the positions of the soft tissues of interest. Hypothetical models of vertebral morphologies (in lateral and anterior view) that are associated with, A, ‘high’, and, B, ‘low’ intervertebral joint stiffness (after [33]). The position of the ‘vertebral disc’ as well as the *interspinalis* and *intertransversarius* ligaments are marked.

**Figure 3:**
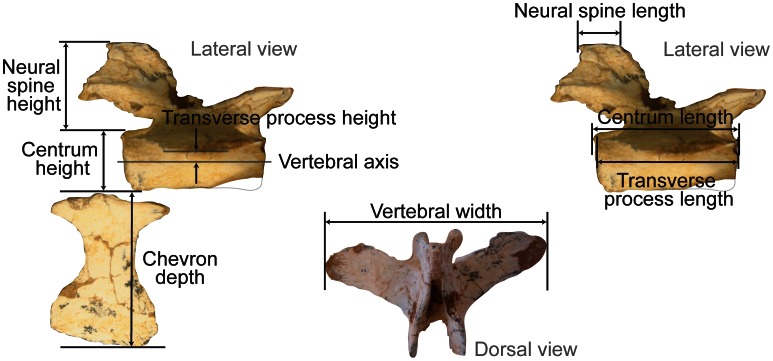
Vertebral parameters measured to reconstruct intervertebral joint stiffness. Eight biomechanically-informative measurements taken from caudal vertebrae to reconstruct intervertebral joint stiffnesses (Caudal from oviraptorosaurid *Citipati osmolskae* (MPC 100/978)).

Following Tyson & Gatesy [32], we adopt the model of Long *et al*. [33] for estimating passive intervertebral joint stiffness in theropod tails (Figs. 2, 3). Our model correlates vertebral morphology with experimentally measured passive intervertebral joint stiffness in dolphins [33] and crocodiles [34]. The model predicts that vertebrae with high joint stiffness exhibit dorsoventrally taller neural spines, centra and transverse processes, dorsoventrally deeper chevrons; craniocaudally longer neural spines and transverse processes; craniocaudally shorter centra; laterally wider centra; and wider spans between the tips of each vertebra’s transverse processes (Fig. 2A, see Materials and Methods). If passive intervertebral joint stiffness is relatively low, the opposite predictions apply (Fig. 2B). These correlations relate to the strain in the soft tissues spanning the joint that resist joint rotation (See Materials and Methods). Active stiffening of the tail by muscular forces was not reconstructed by Long *et al*. [33] and seems to have not been quantified in any living tetrapod tail either [35], [36]. This aspect of tail function cannot yet reliably be reconstructed in extinct theropods. However, active stiffness should be correlated with passive intervertebral joint stiffness to some degree– e.g., taller neural spines stiffen the tail passively but also should correlate with larger epaxial musculature [9], [37], [38] and thus greater active stiffening – and also actuating – ability.

Passive intervertebral joint stiffness is referred to herein, more simply, as joint stiffness. Joint stiffness is closely related to whole tail stiffness through the distribution of soft tissues relative to the bending axis, including the intervertebral disc [39], but this is not always in direct proportion because tail weight and length as well as musculature and other non-arthrological influences also determine whole tail stiffness [40], [41], [42], [43], [44]. Hence we make tentative predictions of how joint stiffness might relate to tail stiffness, in the absence of studies that deal with this relationship specifically. We apply this approach to a broad sample of theropod (and other amniote) taxa to reconstruct the sequence of size-normalised anatomical changes, and joint stiffness, between the nodes Theropoda and Neornithes (extant birds; including Phasianidae). Thus, our goal is to reconstruct how an aerodynamically functioning avian tail [8], [18], [19] evolved from a non-avian tail that probably aided balance [10] and played a major role in terrestrial locomotion [1]. However, passive joint stiffness is only one of several forces that are involved in tail control, which also include inertial [15], [17], gravitational and muscular [1], [2], [4], [5], [6], [8], [9], [19], [45] forces. Additionally, aerodynamic forces produced using feathers potentially could act against gravity [46]. At present it is not possible to include all of the forces involved in tail function into one complete picture of tail evolution.

The tail is only supported by its base so the farther along the tail, the less the load. To get the same deflection per load, we predict a high-low stiffness gradient from proximal to distal (Hypothesis 1). If the number of tail joints scales linearly with tail length, an assumption we will check here for theropod tails, to produce the same angle of deflection in the joints of a shorter, lighter tail, the joints in comparable regions should have lower dorsoventral joint stiffnesses compared to a longer tail (Hypothesis 2). However, this argument does not apply to lateral joint stiffness to the same extent because tail support is not a factor; instead, active tail swinging is the major consideration. All else being equivalent, to sweep through the same arc with a lateral tail swing, the fewer tail joints in a shorter tail must each swing through a larger angle than the joints in a longer tail. The joints of the shorter tail therefore need to be more mobile, which would benefit from having lower lateral joint stiffness (Hypothesis 3). Thus, we predict that evolutionary tail reduction along the theropod lineage involved a stepwise reduction of dorsoventral and lateral joint stiffnesses (Hypothesis 4).

## Analyses and Results

### The Data

We took over 6000 measurements from 38 amniote skeletons at 15 institutions, focussing on coelurosaurian theropods (21) and birds (10); other potentially analogous tails were also studied, such as the tail of a red kangaroo and monitor lizard (Tables S1, S2 in File S1). All measurements were size-normalised using femoral length (Table S1 in File S1). Table S3 in File S1 shows the complete dataset, which contains size-normalised data for all vertebral parameters for all the taxa listed in Table S1 in File S1. Table S4 in File S1 lists average values calculated from the complete dataset by partitioning the tail into three equal regions for each vertebral parameter: proximal, middle and distal (0–33.3%, 33.4–66.6% and 66.7–100% of tail length). Standard deviations, calculated from the complete dataset for the three tail regions for each of the vertebral parameters, remain reasonably low (Table S5 in File S1). This indicates that the values we focus on are close to the average data values (Table S4 in File S1), which implies that subdividing the tail into proximal, middle and distal regions accurately extracts representative morphological information. Consequently, the three regionally averaged data partitions are suitable for reconstructing evolutionary patterns in this study.

### Principal Components Analysis (PCA) Results

**Figure 4:**
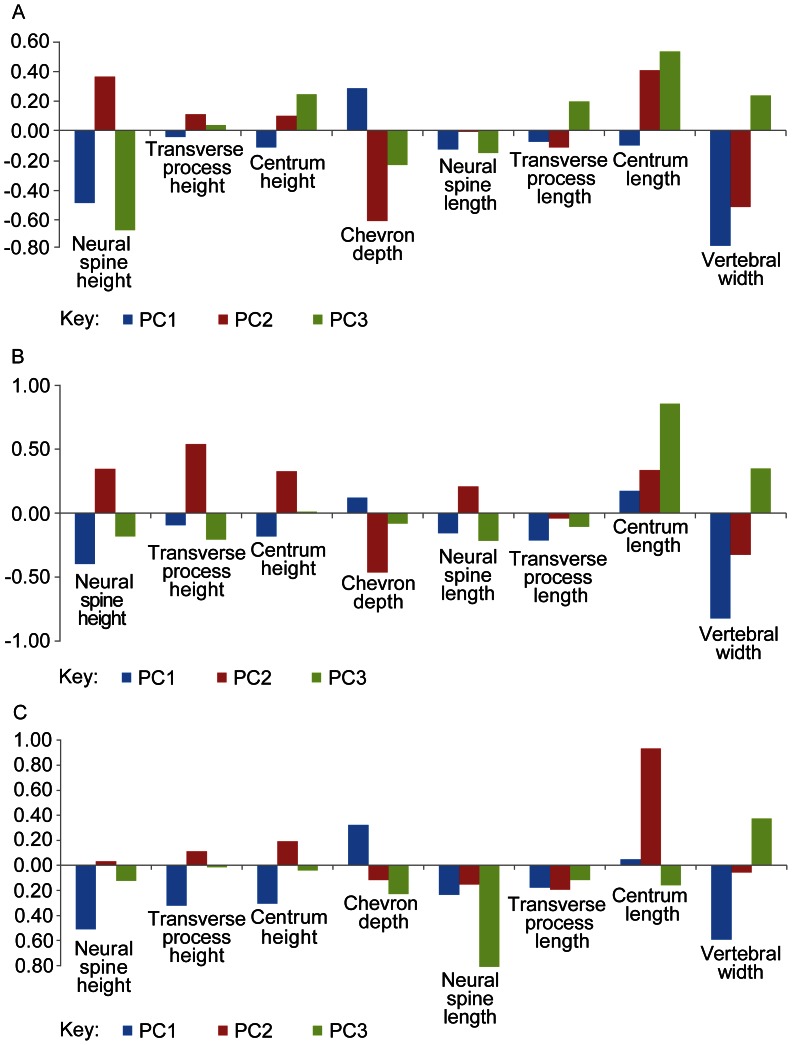
PCA results: vertebral parameter loadings for PCs 1–3. PCAs of complete, theropod-only and non-avian theropod-only datasets - vertebral parameter loadings for PCs 1–3. A, complete dataset. For PC1, neural spine height, chevron depth, and vertebral width explained relatively large portions of the variance. B, theropod-only (outgroups excluded) dataset. For PC1, neural spine height and vertebral width explained large portions of the variance. C, non-avian theropod (no outgroups and Avialae/Aves) dataset. For PC1, neural spine height and vertebral width explained the largest portions of the variance.

Three PCAs evaluated how the vertebral parameters explained the variance in three datasets: the complete dataset, theropod-only data (outgroups excluded) and non-avian theropod-only data (outgroups and Avialae/Aves excluded) (Tables 1, 2, 3, S6 in File S1, Fig. 4). The results helped to assess the degree of similarity in the geometric dimensions of the tails of the non-avian and avian theropods, and the outgroup taxa studied. In all three PCAs (Tables 1, 2, 3), the first three PCs each represented more than 5% of the total variance in the dataset, and combined represented more than 90% of the total variance in the dataset.

**Table 1:** Percentage variance explained by the principal components for the complete dataset.

PC	Eigenvalue	% variance
1	0.033	**75**
2	0.0045	**10**
3	0.0031	**7.0**
4	0.0015	3.3

PCA of complete dataset - the first three PCs explained more than 90% of the total variance, and each individually explained more than 5% of the total variance.

**Table 2:** Percentage variance explained by the principal components for the theropod dataset.

PC	Eigenvalue	% variance
1	0.014	**65**
2	0.0050	**22**
3	0.0015	**6.5**
4	0.00062	2.8

PCA of theropod (outgroups excluded) dataset - the first three PCs explained more than 90% of the variance, and each individually explained more than 5% of the total variance.

**Table 3:** Percentage variance explained by the principal components for the non-avian theropod dataset.

PC	Eigenvalue	% variance
1	0.013	**79**
2	0.0018	**10**
3	0.00085	**5.0**
4	0.00047	2.8

PCA of non-avian theropod (no outgroups and Avialae/Aves) dataset - the first three PCs explained more than 90% of the variance, and each individually explained more than 5% of the total variance.

### PCA of the Complete Dataset

For PC1 in this analysis, neural spine height, chevron depth, and vertebral width explained relatively large portions of the variance (Table S6A in File S1, Fig. 4A). This was also the case in PC2, but centrum length also explained a large proportion of the variance on this axis. For PC3, a large portion of the variance was explained by neural spine height and centrum length.

### PCA of the Theropod Dataset

For PC1 in this analysis, neural spine height and vertebral width explained large portions of the variance (Table S6B in File S1, Fig. 4B), whereas for PC2, transverse process height and chevron depth explained the largest portions of the variance. For PC3, centrum length (and to a lesser extent vertebral width) explained a significant portion of the variance.

### PCA of the Non-avian Theropod Dataset

For PC1 in this analysis, neural spine height and vertebral width again explained the largest portions of the variance (Table S6C in File S1, Fig. 4C), whilst for PC2, centrum length explained most of the variance. For PC3, neural spine length and vertebral width explained most of the variance.

### Key Vertebral Parameters Contributing to PC Variation

Given that the same vertebral parameters (neural spine height and vertebral width) contributed most of the variation on PC1 in all three analyses, all the parameters and taxa within the regionally averaged data (Table S4 in File S1) can be reasonably analysed together. Centrum length contributed to much of the variation in PCs 2 and 3 in all three analyses, so together with neural spine height and vertebral width, these three parameters are the most important to consider in reconstructing intervertebral joint stiffness from the dataset.

### Reconstructed Nodal Values for Tail Parameters

**Figure 5:**
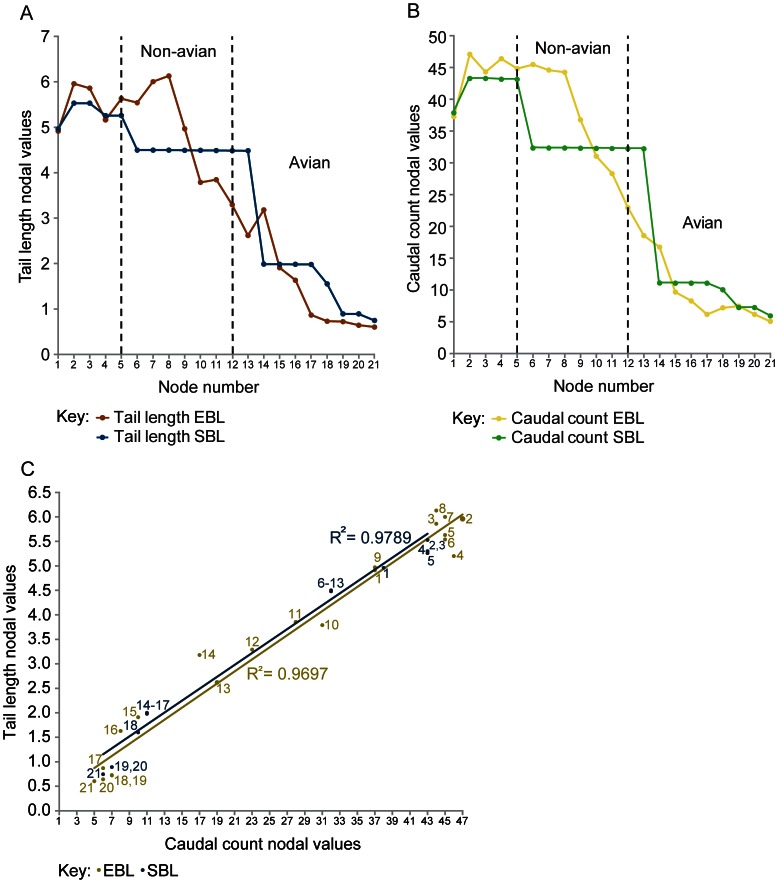
Size-normalised tail length and caudal count nodal values reconstructed for amniotes. Size-normalised amniote nodal values (See Fig. 1; also Materials and Methods): A, tail length, and B, caudal count. Nodes 5–11 are non-avian theropods whereas nodes 12 onwards are birds. Mapping results under EBL and SBL branch length assumptions are labelled as “EBL” and “SBL” respectively. C, tail length and caudal count appear to be proportional (EBL data: y = 0.1233x+0.2553, R^2^ = 0.9697, *r = *0.985 which is significant at the 0.01 level (*p* (2-tailed) = 0.000); SBL data: y = 0.1217x+0.4225, R^2^ = 0.9789, *r = *0.989 which is significant at the 0.01 level (*p* (2-tailed) = 0.000)). Node numbers (1–21) are marked next to each EBL and SBL data point.

**Figure 6:**
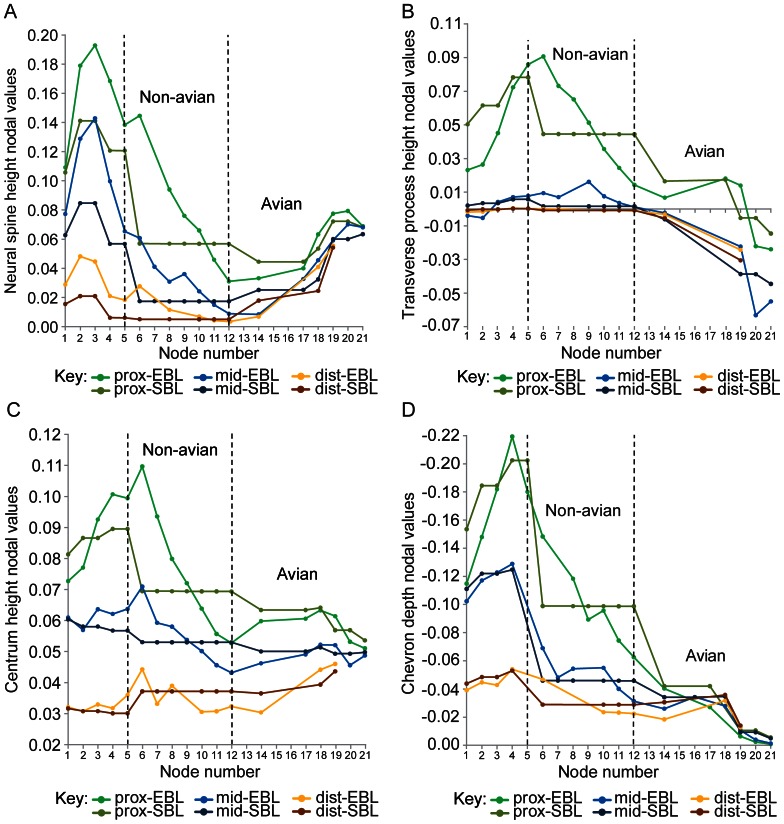
Size-normalised height and depth nodal values reconstructed for amniotes. Size-normalised amniote nodal values: A, neural spine height, B, transverse process height, C, centrum height, and D, chevron depth. The proximal, middle and distal regions of the tail are abbreviated as: “prox”, “mid” and “dist”. See Figure 5 for more information.

**Figure 7:**
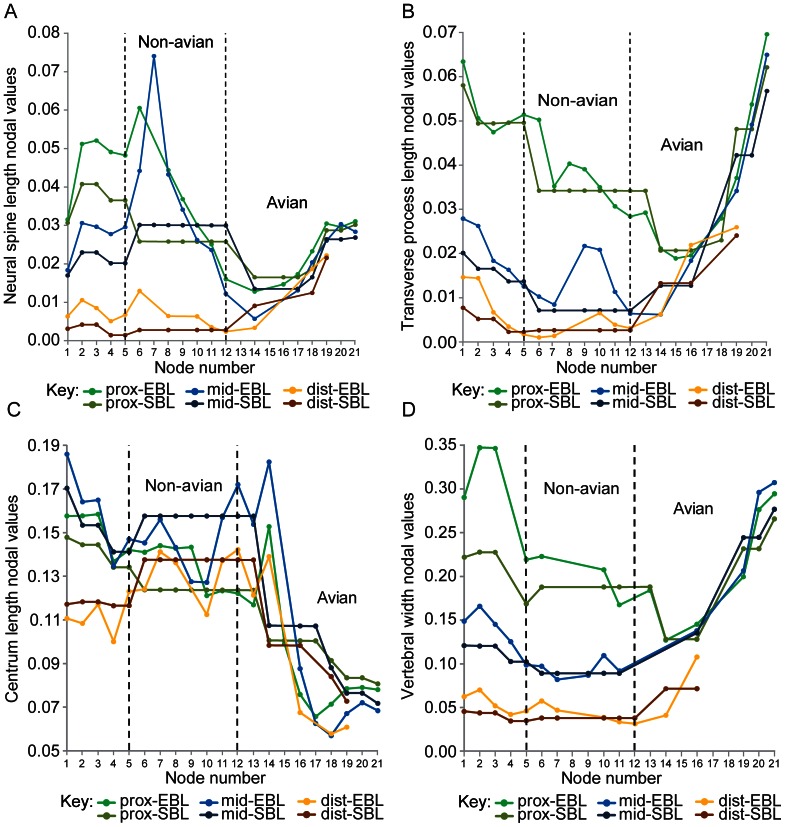
Size-normalised length and width nodal values reconstructed for amniotes. Size-normalised amniote nodal values: A, neural spine length, B, transverse process length, C, centrum length, and D, vertebral width. See Figures 5 and 6 for more information.

Figures 5, 6, 7 display nodal values reconstructed by mapping the averaged data from Tables S4 and S7 in File S1 on a composite theropod-focussed amniote phylogeny (Fig. 1) using equal and stratigraphically calibrated branch length (EBL and SBL) assumptions (See Materials and Methods), to estimate patterns of evolution in theropod tail morphology (Tables S8–S10 in File S1). All results were substantiated by both EBL and SBL analyses, except where stated otherwise. In instances where one set of branch length assumptions showed a constant trend, whereas the other set showed an increasing/decreasing trend, then the latter was favoured as the overall trend. This interpretation was favoured because the average of “no change” and “some change” is still “some change” (See Material and Methods). The EBL and SBL patterns could change as more taxa are included into the dataset, but an attempt was made to minimise these potential changes by sampling tail specimens as evenly across theropod phylogeny as possible. Here we examine the evolutionary trends across major nodes from Amniota to crown group birds (Aves/Neornithes) focussing on those from Theropoda to Phasianidae. The node numbering scheme that was adopted can be found in Figure 1.

### Whole Tail Parameters

Tail length (Fig. 5A): The tail’s length (sum of all centrum lengths and, when applicable, the length of a completely fused pygostyle or the sum of element lengths within a partially ankylosed pygostyle) generally shortened between Theropoda and Phasianidae, in accordance with previous studies [1], [3]. However, there are more subtle, complex patterns that depend on the branch length conditions adopted for the data mapping. The EBL data mapping shows that tail length was similar at Theropoda and Coelurosauria, whereas the SBL mapping results reveal a shorter tail at Coelurosauria than at Theropoda. According to the EBL results, tail length increased to its maximum value at node 8, greatly shortened to node 10, but maintained a similar length to Paraves. In contrast, the tail’s length remained unchanged between Coelurosauria and node 13, according to the SBL data. The EBL data show tail shortening between Paraves and node 13, and lengthening to node 14; however the SBL data show constant tail length between Paraves and node 13, and dramatic shortening between nodes 13 and 14. EBL mapping shows that the tail generally shortened from node 14 to Phasianidae, but was of uniform length between Ornithuromorpha and Phasianidae. The EBL and SBL mapping agree that tail length was constant between Neornithes and Phasianidae because under SBL, tail length was constant between nodes 14 and Ornithuromorpha. However, both mapping results disagree at the remaining nodes because under SBL, tail length decreased from Ornithuromorpha to Neornithes.

Caudal count (Fig. 5B): The caudal count results (total number of vertebrae in the tail excluding the pygostyle) are very similar to the tail length results above: caudal count decreased between Theropoda and Phasianidae. Following the EBL data, caudal count was almost uniform from Theropoda to node 8, and then steadily decreased to Ornithuromorpha. Caudal count then increased slightly from Ornithuromorpha to Neornithes, and decreased slightly to Phasianidae. However, caudal count was relatively similar between Ornithuromorpha and Phasianidae. The SBL results are the same as the SBL results for tail length –except that caudal count decreased more steeply between Theropoda and Coelurosauria, and between nodes 13 and 14, but more shallowly between Ornithuromorpha and Neornithes. Overall, there is more agreement between the EBL and SBL results compared to the tail length results. A graph of tail length against caudal count has a high correlation coefficient; this indicates that the number of tail joints scales linearly with tail length (Fig. 5C). All other parameters being equal, this implies lower dorsoventral joint stiffnesses in comparable regions in shorter, lighter tails compared to longer, heavier ones (supporting Hypothesis 2).

### Tail Parameters Relating to Specific Vertebral Shape Features

Neural spine height (Fig. 6A): In all regions of the tail, the EBL results show that neural spine height generally decreased from Theropoda to Paraves, although this decrease was shallower in the distal tail. At Theropoda, proximal and distal neural spine heights were lower than at Coelurosauria, whereas the middle neural spines were dorsoventrally taller at Theropoda than at Coelurosauria. The proximal and middle tail SBL results indicate higher neural spine heights at Theropoda than at Coelurosauria, but in the distal tail, neural spine height at both nodes was the same. SBL mapping shows constant neural spine heights between Coelurosauria and Paraves.

From Avialae to Phasianidae, neural spine height increased weakly for all regions of the tail, but not to the same height that was found at Theropoda. The EBL and SBL results are contrasting in the proximal and middle tail between Avialae and node 14. However, not all data follow this trend – proximal neural spine height was nearly constant between node 14 and Ornithuromorpha, whilst the proximal tail results of the SBL mapping are most consistent with decreased neural spine height between Avialae and node 14. Neural spine height increased the most steeply between Ornithuromorpha and Neornithes. Neural spine height in the proximal tail decreased slightly between Neognathae and Phasianidae; the same change happened in the middle tail too, but only following the EBL mapping data.

Transverse process height (Fig. 6B): Proximal transverse process height (Figs. 2, 3) was lower at Theropoda than at Coelurosauria, according to EBL data mapping; however the SBL results show that it was dorsoventrally higher at Theropoda than at Coelurosauria. According to EBL mapping, proximal transverse process height decreased sharply from Coelurosauria to Paraves, whereas this height was constant between the same nodes under SBL mapping. In the middle and distal tail, transverse process height was constant between Theropoda and Paraves, and remained at or near the vertebral axis.

From Avialae to Phasianidae, the dorsoventral height of the transverse processes generally decreased, and became positioned below the vertebral axis (negative values in Fig. 6B). However, their height in the proximal tail increased between nodes 14 and Ornithurae under EBL mapping, but was constant between the same nodes under SBL mapping. Similarly, the middle tail EBL results between Neognathae and Phasianidae deviate from the general trend observed because they also increased. Between Neornithes and Neognathae, the SBL results show constant transverse process height for the proximal and middle tail.

Centrum height (Fig. 6C): The EBL mapping shows lower centrum height (all joints) at Theropoda than at Coelurosauria. This trend is opposed by the proximal and middle tail SBL results but supported by the distal tail SBL data. Our findings with the EBL mapping show that centrum height generally decreased between Coelurosauria and Paraves in all regions of the tail, although this decrease was less steep in the distal tail. The SBL mapping reveals that the same centrum height persisted between Coelurosauria and Paraves.

From Avialae to Ornithurae, the EBL mapping indicates that proximal centrum height increased, except for remaining constant between node 14 and Ornithurae, and then decreased to Phasianidae, with the heights at Phasianidae and Avialae similar. SBL mapping suggests that the proximal tail’s mean centrum height generally decreased between Avialae and Phasianidae. However, the SBL results show some exceptions to this trend: a constant proximal centrum height between node14 and Ornithurae, and between Neornithes and Neognathae. In the tail’s middle region, EBL mapping suggests that centrum height increased between Avialae and Ornithurae, retained its height at Neornithes, decreased to Neognathae, and then increased to Phasianidae. In contrast, the SBL results show that middle centrum height was relatively constant. Centrum height in the distal tail decreased very slightly between Avialae and node 14, but then increased to Neornithes.

Chevron depth (Fig. 6D): From Theropoda to Paraves, chevron depth (Figs. 2, 3) broadly decreased according to the EBL results; although it was relatively constant in the middle tail between Maniraptora and node 10. The SBL results show constant chevron depth between Coelurosauria and Avialae; however proximal tail chevron depth was deeper at Theropoda as in the EBL results.

Between Avialae and Phasianidae there was a steep reduction in proximal and middle tail chevron depth, although the EBL results show that chevron depth increased in the middle tail between nodes 14 and 16. According to the SBL results, the exceptions to this general decreasing trend are the nodes where chevron depth remained constant: proximal tail between node 14 and Ornithuromorpha (Ornithurae in middle tail), and between Neornithes and Neognathae. The EBL mapping results reveal that distal chevron depth decreased slightly from Avialae to node 14, and then increased to Ornithurae; but according to the SBL mapping, depth just increased from Avialae to Ornithurae. The EBL and SBL results show decreased distal chevron depth from Ornithurae to Neornithes.

Neural spine length (Fig. 7A): The neural spines were craniocaudally shorter (Figs. 2, 3) at Theropoda than at Coelurosauria, according to the EBL data. In contrast, the SBL results for the proximal tail exhibit the opposite trend, whereas the middle tail has the same pattern as the EBL results, and distal neural spine length was the same at Theropoda and Coelurosauria. The EBL mapping results indicate that the craniocaudal length of the neural spine shortened between Coelurosauria and Paraves –except in the middle tail where neural spine length was significantly shorter at Coelurosauria than at Maniraptora, and in the distal tail where neural spine length was constant between nodes 8 and 10. When we employed SBL data mapping, neural spine length was constant between Coelurosauria and Paraves, but at the theropod node, neural spine length was slightly longer in the proximal tail, but shorter in the middle and marginally in the distal tail.

Along the avian lineage, the proximal and middle tail share similar trends. Between Avialae and node 14, proximal and middle tail neural spine length decreased. Between node 14 and Ornithuromorpha, the EBL results reveal that neural spine length increased, whereas the SBL results show that it remained unchanged. From Ornithuromorpha to Neornithes, neural spine length increased in the proximal and middle tail, and then remained relatively fixed to Phasianidae. Distal tail neural spine length increased between Avialae and Neornithes.

Transverse process length (Fig. 7B): The mean craniocaudal length of the proximal tail’s transverse processes (Figs. 2, 3) shortened overall between Theropoda and Paraves, when EBL mapping was adopted. In the middle and distal tail, the EBL results imply a high degree of variability in transverse process length. For the middle tail, the EBL findings denote craniocaudal shortening of the transverse processes from Theropoda to Maniraptora, then lengthening of them from Maniraptora to node 9. This length was maintained at node 10, but then shortened between node 10 and Avialae. For the distal tail, the EBL mapping suggests that transverse process length was reasonably constant between Theropoda and Maniraptora, increased between Maniraptora and node 10, and then shortened from node 10 to Avialae. Under SBL branch length conditions, transverse process length was constant between Coelurosauria and Avialae. Proximal and middle transverse process length was significantly longer at Theropoda than at Coelurosauria, but both nodes had the same length in the distal tail.

Between Avialae and node 13, proximal tail transverse process length was relatively constant; however it shortened between nodes 13 and 14, and then was similar between nodes 14 and 16. From node 16 to Phasianidae, the craniocaudal length of the transverse processes increased sharply –except between Neornithes and Neognathae where SBL mapping results suggest that transverse process length was constant. In the middle tail, the transverse processes length increased overall between Avialae and Phasianidae; the exceptions to this trend are: the constant lengths between Avialae and node 14 (EBL results), and between nodes 14 and 16 and Neornithes and Neognathae (SBL results). In the distal tail, transverse process length increased between Avialae and Neornithes; however the SBL results suggest that transverse process length was constant between nodes 14 and 16.

Centrum length (Fig. 7C): Our EBL mapping results show that the proximal centra retained a similar length between Theropoda and node 9. Proximal centrum length shortened sharply at node 10 but this did not change up to Paraves. The EBL results for middle and distal centrum length share a common pattern: between Theropoda and Coelurosauria centrum length was constant, and then it increased to Maniraptora, decreased to node 10 (constant between nodes 9 and 10 in middle tail), but then increased to Avialae. According to the results of the SBL mapping, proximal centrum length was longer at Theropoda than at Coelurosauria, whereas middle and distal centrum length was shorter at Theropoda than at Coelurosauria. The results of our SBL mapping also indicate that centrum length remained uniform in all tail regions from Coelurosauria to node 13.

Overall, the caudals shortened dramatically between Avialae and Phasianidae. The EBL mapping indicates that the centra shortened between Avialae and node 13, but then lengthened significantly at node 14 (maximum length recorded). The mapping also shows that centrum length decreased steeply from nodes 14 to 16; then it continued to decrease to Ornithuromorpha in the proximal tail, whereas it continued to decrease to Ornithurae in the middle and distal tail. Our EBL results specify craniocaudal lengthening of the proximal centra between Ornithuromorpha and Neornithes, but a similar length at subsequent nodes up until Phasianidae. The middle and distal tail show the same trend but starting later at Ornithurae. Our SBL results record a dramatic shortening of the centra between nodes 13 and 14. These results also show that the proximal and middle centra kept the same length between node 14 and Ornithuromorpha, and then shortened towards Phasianidae (similar centrum length between Neornithes and Phasianidae). In the distal tail, the SBL results identify the same centra length between nodes 14 and 16, but this shortened to Neornithes. The EBL and SBL data are contradictory at the following nodes: the proximal and middle tail between nodes 13 and 14, and between Ornithuromorpha (from Ornithurae in the middle tail) and Neornithes.

Vertebral width (Fig. 7D): The span between the tips of each vertebra’s transverse processes (vertebral width; Figs. 2, 3) was constant between Theropoda and Paraves –except the proximal tail’s mean vertebral width, which narrowed slightly according to the EBL mapping results. Vertebral width generally increased along the avian lineage. In the proximal tail, the EBL results show that vertebral width increased between Paraves and node 13, whereas the SBL results show that nodes 12 and 13 maintained the same vertebral width crownward from Coelurosauria. Both branch length results show that vertebral width decreased from nodes 13 to 14, and then generally increased to Phasianidae (with the exception of the constant widths between nodes 14 and 16, and between Neognathae and Neornithes (SBL results)). In the middle tail, vertebral width increased from Paraves to Phasianidae, but according to the SBL results width was constant between Neornithes and Neognathae. In the distal tail, the span between the tips of each vertebra’s transverse processes was the same between Paraves and Avialae, and then increased from Avialae to node 16; although it was constant between nodes 14 and 16 according to the SBL results.

### Qualitative Character Mapping Results

**Figure 8:**
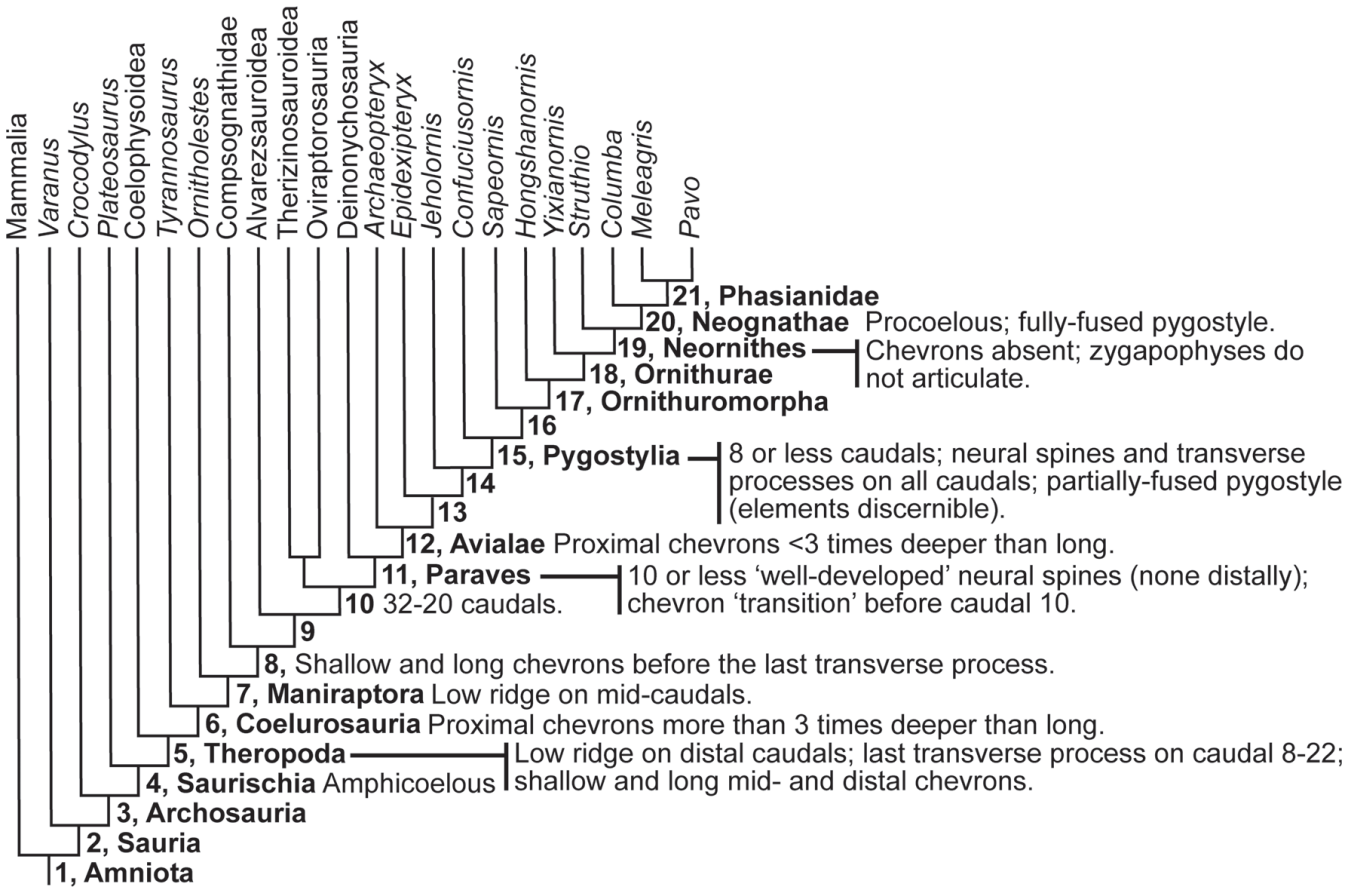
Qualitative character mapping results for the complete amniote dataset. Qualitative tail characteristics reconstructed at amniote nodes using a matrix of data compiled from the entire dataset, and first-hand observations of specimens.

Figure 8 shows the results of mapping qualitative phylogenetic tail character data (Table S12 in File S1) over the composite tree topology displayed in Figure 1 (See Materials and Methods). Between Theropoda and Phasianidae, a proximal shift along the tail of the location of low ridges on the dorsal surface of the centrum (Characters 4,5 in Table S11 in File S1), and of shallow and long chevrons (Characters 10–12), indicate that dorsoventral tail height decreased between these nodes. Dorsoventral tail height was particularly low at Paraves as indicated by 10 or fewer ‘well-developed’ neural spines, and their absence in the distal tail (Characters 3,5). Caudal count decreased between Theropoda and Phasianidae to a low at Pygostylia (8 or fewer caudals (Character 1)). At this node, there were neural spines and transverse processes on each caudal (Characters 1, 3–6), and the tail terminated in a pygostyle (Character 13). At Neornithes, chevrons and articulating zygapophyses were absent (Characters 7–10), and the tail articulated through procoelous rather than amphicoelous intervertebral joints (Character 2).

## Discussion

### The Theropod Lineage to Extant Birds

We found that the tail of theropods changed in complex patterns along the lineage to crown group birds. In addition to the widely known shortening of the tail (and proportionate reduction of joint number) there were, for example, clear reductions of chevron depth, transverse process height and centrum length (Figs. 5, 6, 7). However, how did these changes relate to tail joint stiffness and perhaps even whole tail stiffness? We adopted a novel graphical approach for depicting nodal morphologies along our phylogeny (Fig. 1; see Materials and Methods; also Movie in File S2) and now use these models here to trace potential patterns of the evolution of tail joint stiffness, focusing on four tail ‘types’ that are strongly representative of overall tail changes across the tree. These types were ancestral reconstructions for the nodes for Theropoda, Avialae/Aves, Pygostylia and Neornithes.

**Figure 9:**
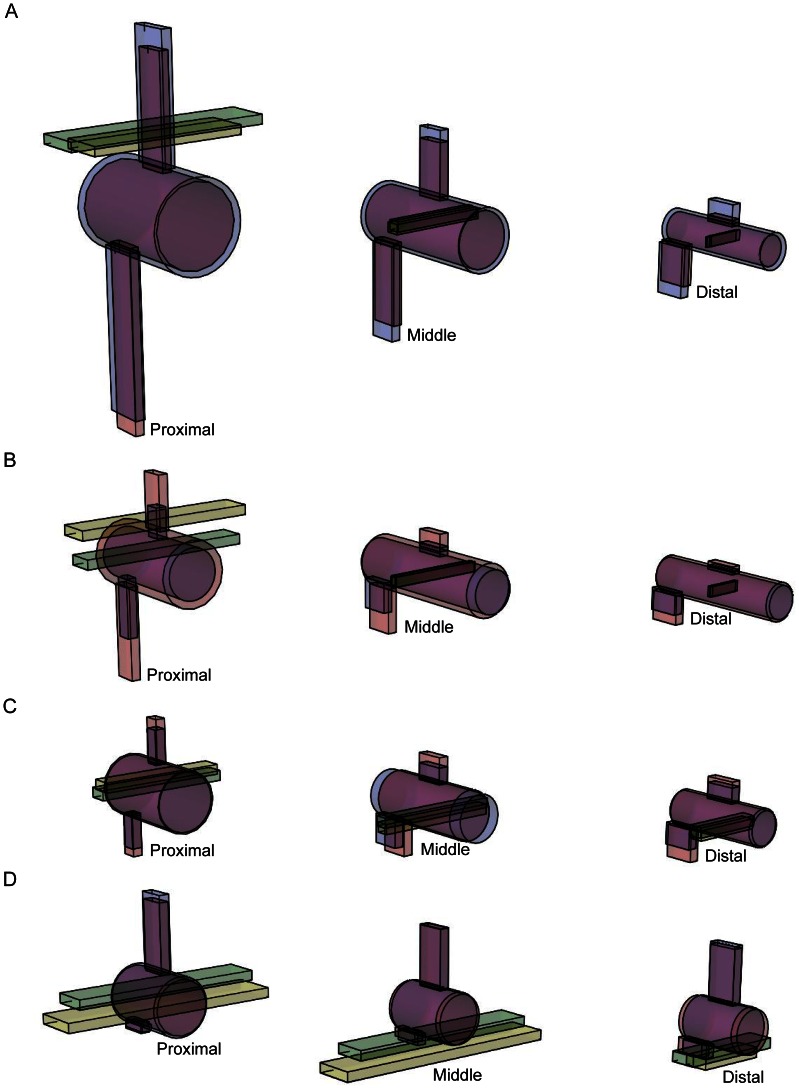
Pictorial renderings of hypothetical proximal, middle and distal caudal vertebrae reconstructed at theropod nodes. Figure 9. Hypothetical pictorial renderings of proximal, middle and distal caudal vertebrae reconstructed at the nodes: A, Theropoda (node 5), B, Avialae (node 12), C, Pygostylia (node 15), and D, Neornithes (node 19).

The ancestral theropod tail (node 5) was characterised by being much taller dorsoventrally than wide laterally, particularly in the proximal and middle tail, as well as having relatively craniocaudally long vertebrae (Fig. 9A). However, the impression of the latter depends on centrum height as well. The dorsoventral and lateral dimensions of the vertebrae diminished along the length of the tail. This tail form was similar to that of ancestral Saurischia (node 4). In the ancestral theropod, therefore, joint stiffness was higher dorsoventrally than laterally, and decreased along these tails.

As predicted by Hypotheses 2–4, dorsoventral and lateral joint stiffness decreased between the theropod and paravian nodes. Decreased dorsoventral joint stiffness (consistent with Hypothesis 2) is supported by decreased proximal transverse process height. It is also supported for all tail regions by decreased neural spine and centrum height, chevron depth, neural spine and tail length, and caudal count. Tail length and caudal count show notable variations within different theropod clades (Figure 1 and Table S7 in File S1) [29] so the addition of new taxa to this study’s dataset might be more likely to affect these two parameters compared to other ones. Decreased lateral joint stiffness (consistent with Hypothesis 3) is supported by decreased proximal transverse process length and vertebral width and also decreased tail length and caudal count. Qualitatively, these trends toward decreasing joint stiffness are supported by a reduction in the height/depth of the neural spines and chevrons, and their extent along the tail, as well as decreased caudal count (Fig. 8). Reduced joint stiffness (supporting Hypothesis 4) potentially reflects a transition from a passively to dynamically stabilizing tail, although this depends on the assumption that tail joint stiffness and whole tail stiffness are somewhat proportional. This transition is plausible because a lighter, less muscular tail with lower stiffness could make greater (e.g., more rapid) use of inertial forces than a stiffer, heavier and muscular tail.

At the avialan/avian node, the tail was also dorsoventrally taller than laterally wide (Fig. 9B) and the caudals decreased in size along its length. Its joint stiffness was therefore higher dorsoventrally than laterally, and decreased distally. Between the paravian and avialan nodes, the dorsoventral and lateral joint stiffnesses for the proximal and middle tail decreased (consistent with Hypotheses 2–4). This is supported by decreased proximal and middle neural spine and centrum height, chevron depth, neural spine and transverse process length, as well as decreased proximal transverse process height between these nodes, in addition to increased middle centrum length. Between the paravian and avialan nodes, distal dorsoventral and lateral joint stiffness was constant (these results neither confirm nor contradict Hypotheses 2–4). This interpretation is supported by all of the vertebral parameters except for centrum length, which increased slightly. Between Avialae and extant birds, most quantitative tail parameters indicate increased dorsoventral and lateral joint stiffness. Increased dorsoventral joint stiffness is inferred in all tail regions from increased neural spine height and increased transverse process depth (negative height values). Increased dorsoventral joint stiffness is also inferred from increased neural spine length in the distal tail. Increased lateral joint stiffness is implied for the middle and distal tail joints by increased vertebral width and the increased craniocaudal length of the transverse processes. Increased lateral stiffness is also implied for all regional tail joints by decreased centrum length, based on Long *et al*.’s [33] interpretations. Our qualitative character data also support this trend, particularly the presence of neural spines and transverse processes on all pygostylian caudals, and the origination of the pygostyle (Fig. 8). Decreased dorsoventral and lateral joint stiffness between Avialae and Phasianidae is favoured by decreased proximal and middle tail chevron depth, as well as decreased tail length and caudal count, in line with Gauthier’s observations of tail shortening, narrowing and lightening along the theropod lineage to extant birds [3]. Decreased dorsoventral and lateral joint stiffness is also recorded between these nodes by reduced caudal count and the reduction and loss of the chevrons in the qualitative data. However, some specimens that were not studied have the potential to alter the tail length and caudal count trends that were recovered. For example, *Epidendrosaurus* (CAGS 02-IG-gausa-1, see Table S2 in File S1) is the sister taxon of *Epidexipteryx* [47] (Table S1) but has a longer tail and a higher caudal count than the latter (22+ compared to 16 caudals respectively [48], [49]). Therefore, if *Epidendrosaurus* had been included in this study the tail length and caudal count reconstructed at node 13 would have been slightly greater (Figs. 1, 5).

Overall, the height and width of the tail vertebrae generally increased between Avialae and Neornithes/Phasianidae (Fig. 9B–D), indicating that dorsoventral and lateral joint stiffness increased between these nodes. The latter is also supported by the stronger weighting assigned to neural spine height compared to chevron depth, for the interpretation of joint stiffness (Table S6, Fig. 4). However, reduced dorsoventral and lateral joint stiffness is still inferred from decreased tail length and caudal count between Avialae and Neornithes/Phasianidae. It is unclear how these two contrasting trends can be reconciled in absolute terms, but it seems more appropriate to place emphasis on the conclusions derived from the vertebral parameters, because these are more directly related to the soft tissues spanning the intervertebral joint. Thus, as the bony tail gets smaller and smaller in birds, dorsoventral and lateral joint stiffness appears to be rising unexpectedly, contrary to Hypotheses 2–4. The reason could be that the tail’s burden/load is now dominated by aerodynamic loads via the feathers. When we see our hypotheses of joint stiffness break down with shorter tails, we appear to be getting a signal of significant rectricial loading. This new design could potentially be tested through experiments involving the short, non-aerodynamic tails of living ratites. Might they follow the original hypotheses (Hypotheses 2–4) even after going through a volant stage? The capacity for increased resistance to aerodynamic loads, as indicated by the joint stiffness trends, allowed birds to utilise increasingly larger lift forces with their tails, including the asymmetrical ones needed for turning manoeuvres [19]. This was probably beneficial to their flight capacity, including both straight flight and turning.

Starting at node 14 (denotes the common ancestor between *Jeholornis* and *Pavo*), but typified at the pygostylian node (15), the tail’s dorsoventral height became similar to its lateral width, and the centra became craniocaudally shorter (Fig. 9C). This tail form persisted to node 16 (denotes common ancestor of *Sapeornis* and *Pavo*). Dorsoventral and lateral joint stiffness was therefore relatively similar between nodes 14 and 16. Joint stiffness also decreased along these tails as their caudals became smaller distally. The first recorded appearance of a pygostyle in the tail of Pygostylia was therefore not associated with a single distinct tail morphology. The proximal and middle sections of the tail in Ornithuromorpha (17) became noticeably wider laterally in relation to its dorsoventral height. Thus, for the first time along the theropod lineage, lateral joint stiffness appears to have exceeded dorsoventral joint stiffness at the ornithuromorphan node.

The novel tail shape and joint stiffness characteristics that first evolved in basal birds became more pronounced at the remaining avian nodes, including the tail of Neornithes (Fig. 9D). The ancestral tail form in Neornithes had dorsoventrally taller neural spines, deeper transverse processes that lie below the vertebral axis, and more widely extended transverse processes than in non-neornithine birds. Thus, dorsoventral and lateral joint stiffnesses continued to increase toward the crown group – in contradiction to Hypotheses 2–4. These morphological characteristics also should have provided later birds with larger moment arms and muscle areas to amplify the forces of tail lifting, depressing and swinging muscles. In the proximal tail of Neornithes, muscular leverage for tail depressor muscles [50], in the absence of chevrons, was switched to the deep transverse processes. Having two transverse processes per vertebra potentially amplified the force of the tail depressor muscles more than a single chevron did ancestrally, because the anchor points of the muscles on each side of the tail were positioned further away from the sagittal plane in the former compared to the latter. However, this is speculative because the modified musculature related to these skeletal changes are uncertain. Whilst the reduction of the chevrons is probably associated with the reduction of the caudofemoralis muscle [1], [2], [3] it is unclear if the development of deeper transverse processes reflects the increased importance of the *M. depressor caudae* or if a wider suite of separate muscles was involved. This speculation therefore needs to be tested quantitatively using more detailed musculoskeletal reconstructions. Such a change in tail depressor muscles could have allowed neornithines to resist and use larger lift forces during flight. However, this ‘deep transverse process’ tail depression mechanism seems to have been present in the middle and distal tail from Avialae crownwards, because these taxa all had transverse processes lying below the vertebral axis. The absence of chevrons in the proximal tail of Neornithes improved tail mobility because they no longer impeded joint movement, particularly in the ventral direction. This increased mobility was further enhanced by the absence of articulating caudal zygapophyses in Neornithes, which removed bone-on-bone forces that restricted joint movement in ancestral birds. This should have allowed neornithines (including extant birds with fully derived tail fans and retricial bulbs) to produce a wider range of muscular force vectors than non-neornithine birds. Consequently, the range of lift force vectors that could be utilised in flight was potentially expanded, supported by other evidence for greater flight proficiency and manoeuvrability in modern birds compared to their predecessors [21], [51], [52], [53], [54], [55], [56].

The singular appearance of a ‘wider than tall’, short but stiffened tail morphology at Ornithuromorpha, that was inherited by Neornithes (Fig. 9D), inspires the conclusion that the fully derived tail fanning observed in modern neornithines [2], an integral element of modern flight ability, was already present at Ornithuromorpha, rather than later at Ornithurae [57]. Thus, the first pygostyles could simply have been associated with tail reduction before tail fanning capabilities evolved [2], [18], [50], [57], although the pygostyle itself might indicate the presence of an incipient form of tail fanning [8], [50]. It is possible that the latter is also true for incipient pygostyles that consist of unfused caudals with strong morphological associations, for example, the last four caudals of *Zhongornis* (D 2455/6, Table S2) form a continuous lateral flange [58]. However, inferring the function of this structure in D 2455/6 is speculative because it is not preserved with a tail fan but only has faint traces of vaned feathers that align with the tail [58]. In contrast, distal tail fronds are preserved in articulation with unfused, closely oppressed distalmost caudals in the oviraptorosaur *Caudipteryx*, as seen in specimens NGMC 97-4-A and 97-9-A (Table S2) and IVPP V12430 (Tables S1 and S2) [59], [60]. However, the mechanism by which these feathers were controlled is unknown [5], even in the oviraptorosaur *Similicaudipteryx* (STM 4-1 and 22-6, Table S2), in which the tail frond is attached to a fused pygostyle-like structure as in birds [61], [62]. In non-ornithuromorphan birds such as *Archaeopteryx* that had yet to evolve a tail fan but instead retained an ancestral tail frond, this feature still conferred aerodynamic improvements to the body that benefitted their gliding and volant abilities [2]. However, the ‘palm-like’ frond of *Jeholornis palmapenis* appears to have lacked any aerodynamic benefit since it does not form a cohesive airfoil. Its frond was most likely used for a display function [63], although it is possible that the feathers of the frond are partially disarticulated so the frond might have had an aerodynamic function, as in *Archaeopteryx* [2]. Tail fronds present in non-avian theropods such as *Microraptor* also could have benefitted the aerial performance of these animals [64], [65], [66], [67].

To summarise, the gradual changes recovered in the tail between Theropoda and Neornithes/Phasianidae might reflect adaptive trends (Figs. 5, 6, 7), although the nature of these changes could be affected by additional data and be different along the side branches of this lineage (See below): reduced dorsoventral and lateral joint stiffness to enable dynamic tail stabilisation [15], [18], [32], and increased dorsoventral and lateral joint stiffness that could have facilitated improvements in flight ability [21], [51], [52], [53], [54], [55], [56]. Nevertheless, testing for an adaptive trend would require other analyses [68]. The inflection of most trends in our data at Avialae (Figs. 6–7) suggests that the reorganization of the hip extensor muscles into separate modules for bipedal locomotion and tail-aided manoeuvring [1], [18], [69] happened early on in avian evolution, and seemingly was a rapid process. Nonetheless, this hypothesis needs to be tested further in light of other important evidence – especially active controls of tail motion that would be represented by tail muscle volumes and fourth trochanter size [1], [6], [9], [37], [70], [71], [72], [73], that are beyond the scope of this study. Other passive determinants of stiffness and mobility, such as articular surface shapes and ranges of joint mobility [50], likewise deserve further quantitative scrutiny as we have only considered them qualitatively.

Side branches along the theropod lineage to extant birds.

Individual side branches in the maniraptoran lineage displayed unique trends in tail stiffness. These include the oviraptorosaur and dromaeosaurid side branches (See below).

### Oviraptorosaur Tail Evolution

Persons *et al*. [5] inferred that the short, broad and deep tails of oviraptorosaurs had ‘a high degree of tail flexibility per unit of absolute tail length’ based on their craniocaudally short and laterally broad prezygapophyses as well as craniocaudally short centra. Short and broad prezygapophyses permitted a larger range of motion (mobility) per joint [74], [75], [76], [77], which could have increased overall tail mobility because the craniocaudally shorter centra allowed the tail to accommodate more joints per unit length [5]. Persons *et al*. [5] reconstructed large muscle volumes in oviraptorosaur tails which indicate that these theropods had a relatively greater capacity for actively stiffening the tail. This compliments the high passive joint stiffness predicted by their short, broad and deep tails, according to the model of Long *et al*. [33]. The muscular tails of oviraptorosaurs were mechanically appropriate for holding up their terminal feather fronds [59], [61], [62], [78] and making active use of their high joint mobility (i.e., ranges of motion) to produce a wide range of muscular force vectors that could possibly have been used to create complex displays, such as those seen in modern birds [79], [80], [81], [82]. The short but muscular tail of oviraptorosaurs also appears to indicate an unusually strong capacity for hip extension for a theropod with such a short tail [1], [5].

### Dromaeosaurid Tail Evolution

The long tails of many dromaeosaurids appear to have been distally stiffened by elongated prezygapophyses and the elongated processes extending from the anterior portions of the chevron tips [4], [15], [83]. Persons *et al*. [4] argued that this stiffening affect was greater in dorsoventral flexion compared to lateral flexion because the second moment of area calculated from three cross-sections made through the articulated tail of *Deinonychus antirrhopus* (YPM 5202, Table S2 in File S1) was greater in the former than the latter. The second moment of area relates to the cross-sectional geometry of the vertebrae, which according to the physics governing the bending of linearly elastic, isotropic and uniformly cross-sectioned beams known as ‘beam theory’, is proportional to the force needed to rotate the vertebral joints [84]. Persons *et al*.’s [4] approach is valuable for determining the tail stiffness of dromaeosaurids because their elongated bony structures prevent a meaningful application of Long *et al.*’s [33] model. However, as Persons *et al*. [4] acknowledge, the elongated prezygapophyses and chevrons were not completely rigid in life, as ‘beam theory’ assumes. Nevertheless, their study provides a more compelling argument that dromaeosaurid tail specimens preserved in different bending orientations (*Velociraptor*, MPC 100/25 and 100/986; *Bambiraptor*, AMNH 001 and *Saurornitholestes*, TMP 1982.26.1 and 1988.121.39; Tables S1 and S2 in File S1) might have been genuine and not simply preservational artefacts [4], [85], [86], [87]. Calculating the second moment of area in other tail specimens in conjunction with other biomechanical modelling would nonetheless be useful for investigating dromaeosaurid tail mobility in the future.

Biomechanical modelling and experiments have suggested that dromaeosaurids probably used their tails as dynamic stabilisers [15], [17], so Persons *et al*.’s [4] second moment of area calculations imply that the distal tail contributed more dynamic stabilisation laterally than dorsoventrally. Some dromaeosaurids such as *Utahraptor* (BYU 15465, Table S2 in File S1) appear to have secondarily shortened their prezygapophyses and chevrons, producing a more mobile tail that was suggested to have been a response to the biomechanical demands of a larger body size [83]. Persons *et al*. [4] used their calculations and volumetric reconstructions indicating small tail muscles as possible evidence of secondary flightlessness or a secondary loss of gliding ability in dromaeosaurids. Specimens BMNHC PH881, IVPP V13352 and TNP 00996 of *Microraptor* (Tables S1 and S2 in File S1) preserve distal tail fronds that suggest that at least some dromaeosaurid tails had potential aerodynamic and display capabilities [64], [65], [66], [67], [88], [89]. Secondary flightlessness or a secondary loss of gliding ability in dromaeosaurids [90], [91], [92], [93], [94] could dramatically alter our understanding of flight evolution but this hypothesis has yet to gain wide acceptance.

### Conclusions

The passive stiffness of tail joints changed dramatically along the theropod lineage between Theropoda and extant birds. Our results support the traditional view of theropod tail function, which distinguishes between a non-avian theropod tail primarily used for balance, and a tail of Avialae/Aves used more aerodynamically, and provides new details of how these functions evolved. Initially, the tails of non-avian theropods were suited for support against gravity, via passive tail joint stabilisation. However, non-avian theropods gradually became more dominantly stabilized by dynamic properties as dorsoventral and lateral joint stiffness decreased towards the paravian node, as predicted by Hypotheses 2–4. This functional transition, in addition to tail shortening, might have offset detriments to their turning ability caused by their long, massive tail [10]. In contrast, the tail joints of birds became stiffer dorsoventrally and laterally between Avialae and the crown group which contradicts our original expectations (Hypotheses 2–4). This therefore indicates a reversal of the joint stiffness trend observed between Theropoda and Paraves. This unexpected trend of increased joint stiffness appears to be a signal of significant rectricial loading. Increased joint stiffness would have enabled birds to produce larger muscular forces allowing them to use larger lift forces. Greater tail joint mobility in neornithine birds, because of the absence of chevrons as well as articulating zygapophyses, probably enabled more extensive adjustments to the shape and inclination of the aerodynamic surface compared to ancestral birds. Such changes would have allowed production of a wider range of lift force vectors, lending support to the idea that extant birds are generally more flight proficient and manoeuvrable than their predecessors [21], [51], [52], [53], [54], [55], [56]. The tail skeleton of the common avian/avialan ancestor, present by at least the Bathonian, had a ‘non-avian theropod’ form that remained largely unchanged until approximately 30 million years later in the Barremian, when tail fanning capabilities of potentially incipient and modern aspects first appeared at the pygostylian and ornithuromorphan nodes respectively. However, the fronds of feathers that are attached to the distal tails of basal avialans and some non-avian dromaeosaurid theropods might still have conferred important aerodynamic capabilities [2], [64], [65], [66], [67]. It is possible that tail fanning capabilities were not limited to avian theropods, as suggested by the bird-like tail frond and pygostyle association found in *Similicaudipteryx,* but this is speculative in the absence of an empirically supported control mechanism [5], [61], [62]. We infer that despite a slow start, the core function of the modern tail locomotor module as a precisely controlled aerodynamic surface [18] was established early in avian history. This helps to explain how the tail locomotor module has become so highly elaborate [18] in the more than 10,000 species of living birds [56].

In this study, we have provided an important first step focusing on experimentally supported determinants of passive intervertebral joint stiffness that are evident from vertebral morphology, and how these determinants reveal the evolution of tail joint stiffness. This study will therefore contribute towards broader reconstructions of tail evolution that incorporate other forces acting on the tail including inertial, gravitational, aerodynamic and muscular ones. Our simple biomechanical approach, adapted from [33], has great potential to reveal the functional capabilities and evolutionary histories of other remarkable dinosaur tails, e.g., armoured thyreophorans (Ornithischia) and whip-lash diplodocids (Sauropodomorpha), as well as the tails, backs and necks of other vertebrates. We have also presented a novel technique for reconstructing the 3D morphology of vertebral form for ancestral nodes with a simple graphical display (Fig. 9) that has promise for reconstructing other aspects of the evolution of the axial column.

## Materials and Methods

We confirm that permission was obtained to access specimens housed in the collections of the following institutions (See Table S2 in File S1 for institutional abbreviations): American Museum of Natural History, Bayerische Staatssammlung für Paläontologie und Geologie, Chinese Academy of Geological Sciences, Institute of Vertebrate Paleontology and Paleoanthropology, Jura Museum, Mongolian Paleontological Centre, Museum für Naturkunde Berlin, Oxford University Museum of Natural History, Smithsonian Institution National Museum of Natural History, The Natural History Museum (London), Tianjin Museum of Natural History, University Museum of Zoology, UCL Grant Museum of Zoology and Comparative Anatomy, University of California Museum of Paleontology, Yale Peabody Museum. These specimens were excavated (fossils) and/or prepared (modern animals) by these institutions.

The multivariate ANOVA analysis of Long *et al*. [33] identified vertebral measurements that correlated well with experimental measurements of intervertebral joint stiffness. These results were explained by lever and beam mechanics [84], and were summarised as two hypothetical models of the predicted geometry of vertebrae with relatively high and relatively low joint stiffness in dorsoventral bending. Vertebrae with relatively high joint stiffness have: dorsoventrally taller neural spines, centra and transverse processes, dorsoventrally deeper chevrons; craniocaudally longer neural spines and transverse processes; craniocaudally shorter centra; laterally wider centra; and wider spans between the tips of each vertebra’s transverse processes (Fig. 2A). Tails with relatively low joint stiffnesses have vertebrae with the opposite geometric characteristics (Fig. 2B). Of these characteristics, the craniocaudal length of the transverse processes, the span between the tips of a vertebra’s transverse processes, and centrum width are related to lateral joint stiffness. With the exception of centrum length, which appears to be related to both dorsoventral and lateral joint stiffness, the remaining vertebral parameters are related to dorsoventral joint stiffness. These principles should apply for qualitatively and comparatively assessing the intervertebral joint stiffness of almost any vertebrate taxon from morphology, because they simply relate Newtonian mechanics to morphology and Newton’s laws apply similarly to all vertebrates. However, the absolute quantitative relationships between stiffness and morphology (as Long *et al.* [33] determined with their ANOVA) are certain to vary due to evolutionary changes in the contributions of different structures and tissues to joint stiffness.

The mechanical explanation for these correlations relates to the strain in the soft tissues spanning the joint. For example, an *interspinalis* ligament running from one neural spine to the next (Fig. 2) will be loaded in tension when the intervertebral joint is flexed in the ventral direction. A dorsoventrally taller neural spine positions ligamentous tissue farther above the axis of bending; this creates a longer moment arm for this tissue to leverage its resistance to ventrally directed joint rotation. By the same argument, a laterally wide *intertransversarius* ligament between two adjacent transverse processes (Fig. 2) resists lateral joint rotation. This lever mechanics argument is also applicable to transverse process height and chevron depth, and their associated soft tissues (Fig. 2). Thus, vertebral shape confers directional differences in joint stiffness: dorsoventrally and laterally. Centrum height (which approximates intervertebral disc height; Fig. 2) is positively correlated with dorsoventral joint stiffness because under a constant load, a taller disc bends less dorsoventrally than a shorter disc. Centrum height also approximates centrum width because the centrum is roughly circular. Centrum width affects joint stiffness in the same way as centrum height but in the lateral plane. The craniocaudal lengths of the neural spines and transverse processes are inversely correlated with the length of *interspinalis* and *intertransversarius* ligaments that span between adjacent neural spines, and neighbouring transverse processes. For a craniocaudally longer transverse process which leaves space for a craniocaudally shorter *intertransversarius* ligament, this ligament will be in tension more during a given amount of lateral joint rotation, compared to the situation if it was craniocaudally shorter. More muscle tension increases the amount of resistance to lateral joint rotation; thus the craniocaudal length of the transverse process is proportional to lateral joint stiffness (the same argument applies to the affect of neural spine length on dorsoventral joint stiffness). More difficult to explain is the effect of centrum length on joint stiffness. The length of a centrum should be proportional to the stiffness of the joints that border it because the length of a centrum is proportional to length of the tail it belongs to, although the latter relationship is more weakly supported by distal centrum length data (Figure S1 in File S3). The reason why the relationship between centrum length and joint stiffness can be inferred is because joint stiffness is correlated with tail length, as outlined in the introduction. Contrarily, Long *et al*. [33] identified that centrum length was inversely correlated with stiffness. This suggests that centrum length is correlated with other vertebral parameters that are related to intervertebral tissues. Alternatively, this might be because centrum length affects the length of muscle spanning the vertebrae, which might be inversely correlated with joint stiffness. Given that Long *et al*.’s [33] interpretation is based on experimental data, centrum length is treated as an inverse correlate of joint stiffness. However, the uncertainty in this relationship means that this vertebral parameter should carry a lower weighting when joint stiffness is inferred.

The measurements taken from each caudal vertebra exclude soft tissue parameters absent in fossils (Fig. 3). Specimens with severe distortions were avoided, but in some instances, specimens with minor translational distortions were used. To correct for the distortion in the latter, vertebral features were measured in pairs and an average was taken of these measurements. Chevrons were positioned with the caudals lying cranial to them.

All measurements were size-normalised using femoral length (Table S1 in File S1) because its utility as a reasonable, simple body size proxy has been demonstrated in theropods [95]. However, there are a wide range of proxies available for body size. To assess the relative contribution of the eight vertebral measurements to morphological variation in the tail, principal components analysis (PCA) was used to calculate the major axes of variation using data from complete vertebrae. This was performed in the statistical software PAST [96] in three separate analyses (Fig. 4) using: all of the taxa, theropods only (no outgroups), and non-avian theropods only (no outgroups and Avialae/Aves). A standard significance level of 5% was used to identify the number of PCs that explain large proportions of the dataset. The loadings of the eight vertebral measurements were further analysed to determine which contribute most to the variation explained by each significant PC (Tables 1, 2, 3, Table S6 in File S1). For each of the taxa in the entire dataset average values were calculated for each vertebral parameter within three equal tail portions (the proximal, middle and distal tail represent 0–33.3%, 33.4–66.6% and 66.7–100% of tail length respectively). However, these averages excluded extrapolated measurements. This approach maximises the number of taxa that can be included in the analyses, and makes it easier to compare tail stiffness between taxa (Table S4 in File S1). To ensure that these average values accurately reflect the morphology of the three tail regions, a standard deviation (σ) was calculated to measure the degree of variation about the averages (Table S5 in File S1). Low standard deviations imply that the measurements in the tail region are close to the average values, so do reflect the region’s tail morphology. High standard deviations imply the opposite conclusions.

Evolutionary patterns were reconstructed for each of these three tail portions using Mesquite (v2.7.5) software [97] on an up-to-date composite phylogeny for non-avian coelurosaurs and basal and modern birds [47], [57], [58], [98], [99], [100], with additional dinosaurian taxa and non-dinosaurian outgroups [101] (Fig. 1). While much of the theropod phylogeny used here has reached a relative consensus, some areas such as maniraptoran or basal bird relationships should be re-examined once consensus is reached. Nodal values were optimized using squared-change parsimony [102], one of several methods that average data over a tree topology to reconstruct ancestral states [97]. Squared-change parsimony is the default setting in Mesquite (v2.7.5), but other methods produced qualitatively similar results. Two branch length scenarios were used to incorporate the effects of gradualistic and punctuated equilibrium evolutionary models [103], [104], [105]. Equal branch lengths (EBL) (lengths of one) approximated the gradualistic model, whereas stratigraphically-calibrated branch lengths (calculated from stratigraphic and ghost ranges) (SBL) approximated the punctuated equilibrium model. These models are unrealistic but should roughly bracket actual theropod tail evolution, which can be interpreted with greater confidence in cases where both models agree. In instances where an EBL or SBL result indicates a near-constant value across multiple nodes under one set of branch length assumptions, but a trend of increase/decrease for the other set, then the latter would be favoured as the qualitative conclusion (because the average of “no change” and “some change” is still “some change”). For example, an increase in the EBL results and a constant SBL result should correspond to an overall increase, although the size of this increase would be indeterminable. In contrast, opposing EBL and SBL trends cannot be interpreted because the trends cannot simply be reconciled as a moving average. The quantitative results were analysed from graphs of each vertebral parameter.

Pictorial renderings (Fig. 9, Movie in File S2) provide general comparisons with the hypothetical models of high and low intervertebral joint stiffness (Fig. 2), which helped with the interpretation of the graphed results (Figs. 5, 6, 7). Thus, the renderings only had a support role in the data analysis, and as such were non-essential to it but are valuable for data interpretation, especially to assess overall changes in joint stiffness. To convert the raw data into renderings, a mathematical model was created in the 3D modelling software, Autodesk Maya 2010 [106]. The mathematical model could not accommodate missing data, so these were filled artificially using the linear interpolation. Since not all vertebral measurements were collected, some assumptions were necessary in the mathematical model: centrum width was made to equal centrum height (measured); the craniocaudal length of each chevron was made to equal neural spine length (measured); the neural spines and transverse processes were placed mid-way along the craniocaudal length of the centrum; and the chevrons were placed in articulation with the lateral mid-point of the ventral edge of the posterior articular face of the centrum. In addition, the renderings were colour-coded so that blue shading, and green shading for the transverse processes, represented nodal values derived from mapping on an EBL tree. Red shading, and yellow shading for the transverse processes, denoted values mapped on an SBL tree. As the missing data and the model assumptions were known, the pictorial renderings were evaluated with this in mind.

Qualitative phylogenetic tail characters were developed from the quantitative tail data, and from firsthand observations of tail specimens (Table S11 in File S1). These characters were scored in a matrix from all the tail specimens studied (Table S12 in File S1). The data matrix was mapped over the composite tree topology (Fig. 1) in a similar way to the quantitative data to produce the results summarised in Fig. 8. The majority of the phylogenetic tail characters broadly reflect the geometric proportions of the tail, so as well as being explained in the same way as the quantitative vertebral parameters, we would also expect them to yield similar results to the quantitative data. Qualitative characters (such as the geometry of the articular faces of the centrum, the length of the prezygapophyses, and the presence/absence of the pygostyle) also capture information relevant to inferences of the tail’s range of movement. For example, procoelous articular faces have a tapered rim that should have allowed the intervertebral joint to bend more before it was impeded. The length of the prezygapophysis indicates the extent of bone-on-bone resistance to joint movement. As a series of fused distal caudals, the presence of a pygostyle indicates that the tail tip had maximal stiffness and minimal mobility.

## Supporting Information

File S1 **Supporting Information Tables S1–12 including a list of the taxa studied as well as the complete dataset.** Table S1. List of 31 coelurosaurian theropods (including birds) studied (and 7 outgroup taxa) and their associated femoral lengths in millimetres. Table S2. Abbreviations for the institutions visited as part of data collection and whose specimens have been discussed in the paper but from which data were not collected. Table S3. Complete dataset (all taxa studied and all vertebral parameters). Taxon abbreviations are given in Table S1. The loss of a vertebral feature is indicated by the zero values in bold. Table S4. Average values for the three regions of the tail using the complete dataset. ‘Prox’, ‘mid’, and ‘distal’ represent the proximal, middle and distal tail regions respectively. Taxon abbreviations are given in Table S1. Table S5. Standard deviations for the complete dataset (Table S3). Taxon abbreviations are given in Table S1. Table S6. Vertebral parameter loadings on the first three principal components: A, complete dataset, B, theropod dataset (outgroups excluded), and C, non-avian theropod dataset (no outgroups and Avialae/Aves). Table S7. Size-normalised tail lengths for all of the taxa studied (sum of all centrum lengths and, when applicable, the length of a completely fused pygostyle or the sum of element lengths within a partially ankylosed pygostyle) and the caudal counts of these tails (number of caudal vertebrae excluding the pygostyle). Missing values were filled artificially using linear interpolation and extrapolation. Table S8. Reconstructed nodal values for the vertebral parameters using EBL assumptions (interpolated values in bold font). ‘Prox’, ‘Mid’ and ‘Dist’ represent the proximal, middle and distal tail regions. Node numbers correspond to those in Figure 1. Table S9. Reconstructed nodal values for the vertebral parameters using SBL assumptions (interpolated values in bold font). ‘Prox’, ‘Mid’ and ‘Dist’ represent the proximal, middle and distal tail regions. Node numbers correspond to those in Figure 1. Table S10. Tail length (size-normalised) and caudal count nodal values using EBL and SBL assumptions. Node numbers correspond to those in Figure 1. Table S11. List of functionally informative, qualitative phylogenetic theropod tail characters (1–13) for character mapping. Table S12. Matrix of functionally informative, qualitative phylogenetic theropod tail data. ‘?’ denotes missing data, whilst ‘-’ is a character that is not applicable to the taxon coded. The character list (1–13) is given in Table S11.
(DOCX)

File S2 **Supporting Information Movie showing a sequence of hypothetical pictorial renderings of proximal, middle and distal caudal vertebrae reconstructed between the nodes, Amniota and Phasianidae (nodes 1 to 21).** See Materials and Methods for more information.
(MOV)

File S3 **Supporting Information Figure showing the correlations between size-normalised tail and centrum length nodal values reconstructed for amniotes.** Figure S1. Correlations between size-normalised amniote tail and centrum length nodal values: A, tail length shows a strong linear correlation with proximal centrum length (EBL data: y = 59.164x−3.5387, R^2^ = 0.8551, *r = *0.925 which is significant at the 0.01 level (*p* (2-tailed) = 0.000); SBL data: y = 79.919x−5.716, R^2^ = 0.9504, *r = *0.975 which is significant at the 0.01 level (*p* (2-tailed)* = *0.000)), B, tail length also shows a strong linear correlation with middle centrum length (EBL data: y = 38.284x−1.3233, R^2^ = 0.6211, *r = *0.788 which is significant at the 0.01 level (*p* (2-tailed) = 0.000); SBL data, y = 48.134x - 2.7588, R^2^ = 0.8856, *r = *0.941 which is significant at the 0.01 level (*p* (2-tailed) = 0.000)), C, tail length shows a weak linear correlation with distal centrum length (EBL data: y = 44.356x−0.9222, R^2^ = 0.4356, *r = *0.660 which is significant at the 0.01 level (*p* (2-tailed) = 0.005); SBL data: y = 54.93x−2.5022, R^2^ = 0.5878, *r = *0.767 which is significant at the 0.01 level (*p* (2-tailed) = 0.001)). Node numbers (1–21, Fig. 1) are marked next to each EBL and SBL data point.
(DOCX)
