# Supplementary material for: Shake a Tail Feather: The Evolution of the Theropod Tail into a Stiff Aerodynamic Surface
Source: PLoS One. 2013 May 15;8(5):e63115. doi: 10.1371/journal.pone.0063115 (PMC3655181; doi:10.1371/journal.pone.0063115)
Supplement: File S1 — Supporting Information Tables S1–12 including a list of the taxa studied as well as the complete dataset. Table S1. List of 31 coelurosaurian theropods (including birds) studied (and 7 outgroup taxa) and their associated femoral lengths in millimetres. Table S2. Abbreviations for the institutions visited as part of data collection and whose specimens have been discussed in the paper but from which data were not collected. Table S3. Complete dataset (all taxa studied and all vertebral parameters). Taxon abbreviations are given in Table S1. The loss of a vertebral feature is indicated by the zero values in bold. Table S4. Average values for the three regions of the tail using the complete dataset. ‘Prox’, ‘mid’, and ‘distal’ represent the proximal, middle and distal tail regions respectively. Taxon abbreviations are given in Table S1. Table S5. Standard deviations for the complete dataset (Table S3). Taxon abbreviations are given in Table S1. Table S6. Vertebral parameter loadings on the first three principal components: A, complete dataset, B, theropod dataset (outgroups excluded), and C, non-avian theropod dataset (no outgroups and Avialae/Aves). Table S7. Size-normalised tail lengths for all of the taxa studied (sum of all centrum lengths and, when applicable, the length of a completely fused pygostyle or the sum of element lengths within a partially ankylosed pygostyle) and the caudal counts of these tails (number of caudal vertebrae excluding the pygostyle). Missing values were filled artificially using linear interpolation and extrapolation. Table S8. Reconstructed nodal values for the vertebral parameters using EBL assumptions (interpolated values in bold font). ‘Prox’, ‘Mid’ and ‘Dist’ represent the proximal, middle and distal tail regions. Node numbers correspond to those in Figure 1. Table S9. Reconstructed nodal values for the vertebral parameters using SBL assumptions (interpolated values in bold font). ‘Prox’, ‘Mid’ and ‘Dist’ represent the proximal, midd [file pone.0063115.s001.docx]

File S1. Supporting Information Tables

Shake a tail feather: the evolution of the theropod tail into a stiff aerodynamic surface

Michael Pittman^1^*, Stephen M. Gatesy^2^, Paul Upchurch^1^, Anjali Goswami^1,3^, John R. Hutchinson^4^

^1^Department of Earth Sciences, University College London, Gower Street, London, WC1E 6BT, U.K.

^2^Department of Ecology and Evolutionary Biology, Brown University, Providence, RI, 02912, U.S.A.

^3^Department of Genetics, Evolution, and Environment, University College London, Wolfson House, Stephenson Way, London, NW1 2HE, U.K.

^4^Structure & Motion Laboratory, Department of Comparative Biomedical Sciences, The Royal Veterinary College, Hawkshead Lane, Hatfield, AL9 7TA, U.K.

*mpittman@hku.hk

Current address: Department of Earth Sciences, The University of Hong Kong, Pokfulam, Hong Kong.

Twelve tables (Tables S1-12) comprise File S1; these are listed as follows:

Table S1. Taxa studied and their femoral length measurements

Table S2. Institutional abbreviations

Table S3. Complete dataset

Table S4. Average values for the three regions of the tail, for all taxa and parameters

Table S5. Standard deviations of complete dataset data

Table S6. Vertebral parameter percentage loadings (PCA results)

Table S7. Tail length and caudal count data, and the source of caudal count estimates

Table S8. Reconstructed nodal values for tail parameters using EBL assumptions

Table S9. Reconstructed nodal values for tail parameters using SBL assumptions

Table S10. Reconstructed nodal values for tail length and caudal count using EBL and SBL assumptions

Table S11. List of functionally informative qualitative phylogenetic tail characters

Table S12. Character matrix for functionally informative qualitative phylogenetic tail characters

Table S1 (below) lists the name and catalogue number of the specimens studied as well as the length of any femurs that are associated with these specimens. The repositories of these specimens are listed in Table S2 (also below).

Table S1. Amniote taxa studied and their femoral length measurements

| Taxon | Abbreviation | Catalogue number | Femoral length in mm (length parallel to the femoral shaft between the top of the femoral head and the base of medial distal condyle) |  |  |
| --- | --- | --- | --- | --- | --- |
| *Coelophysis bauri* | *Coel* | UCMP cast | 186 (right femur with broken femoral head and broken distal end) |  |  |
| *Dilophosaurus wetherilli* | *Dilo* | UCMP V4214/37302 | 554 (left) |  |  |
| *Tyrannosaurus rex* | *Trex* | OUMNH Stan cast | 1321 (from [1]; length parallel to the right femoral shaft between the top of the greater trochanter and the base of the lateral distal condyle) |  |  |
| *Juravenator starki* | *Jura* | JME Sch 200 | 53.30 (right) |  |  |
| *Compsognathus longipes* | *Comp* | BSPG AS I 563 | 77.53 (left) |  |  |
| *Ornitholestes hermanni* | *Ornit* | AMNH 619 | 189 (left femur with broken distal end) |  |  |
| *Shuvuuia deserti* | *Shuv* | MPC 100/1305 | 74.28 (length parallel to the left femoral shaft between the top of the greater trochanter and the base of lateral distal condyle; from specimen MPC 100/99) |  |  |
| *Citipati osmolskae* | *Citi* | MPC 100/978 | 358 (right femur measured to the base of the lateral distal condyle) |  |  |
| *Khaan mckennai* | *Khaa* | MPC 100/1127 | 189 (right) |  |  |
| *Caudipteryx zoui* | *Caud* | IVPP V12430 | 147.48 (right) |  |  |
| *Mei long* | *Mei* | IVPP V12733 | 76.31 (right) |  |  |
| *Sinornithoides youngi* | *Sino* | IVPP V9612 | 119.16 (right femur with unexposed distal tip) |  |  |
| *Sinusonasus magnodens* | *Sinu* | IVPP V11527 | No associated femur. Femoral length from *Sinornithoides*. |  |  |
| *Mahakala osmolskae* | *Maha* | MPC 100/1033 | 78.26 (left) |  |  |
| *Deinonychus antirrhopus* | *Dein* | YPM 5203 | 336 (right; MCZ 4371) |  |  |
| *Velociraptor mongoliensis* | *Velo* | MPC 100/985 | 187 (left) |  |  |
| *Velociraptor mongoliensis* | *Vel_*F | MPC 100/25 | 185 (left) |  |  |
| *Bambiraptor feinbergi* | *Bamb* | AMNH 001 | 115.80 (left) |  |  |
| Undescribed Liaoning dromaeosaurid | *Drom* | IVPP 2008.5 | 41.87 (left femur with broken distal tip) |  |  |
| *Microraptor gui* | *M.gui* | IVPP V13352 | 72.61 (left femur with broken distal tip) |  |  |
| *Microraptor zhaoianus* | *M.zha* | CAGS 20-8-001 | 74.75 (right femur from [2]) |  |  |
| *Epidexipteryx hui* | *Epid* | IVPP V15471A | 45.13 (right femur with broken distal tip) |  |  |
| *Archaeopteryx lithographica* | *Ar.*B | BSPG S6 | 48.77 (left) |  |  |
| *Archaeopteryx lithographica* | *Ar.*J | JM 2257 | 37.08 (left) |  |  |
| *Archaeopteryx lithographica* | *Ar.*H | HMN 1880 | 52.45 (right) |  |  |
| *Jeholornis prima* | *Jeho* | IVPP V13274 | 75 (right femur from [3]) |  |  |
| *Sapeornis chaoyangensis* | *Sape* | IVPP V13276 | 72.10 (left femur measured to the base of the lateral distal condyle ) |  |  |
| *Confuciusornis sanctus* | *Conf* | IVPP V11374 | 45.60 (right) |  |  |
| *Hongshanornis longicresta* | *Hong* | IVPP V14533 | 21.64 (left femur measured to the base of the lateral distal condyle) |  |  |
| *Yixianornis grabaui* | *Yixi* | IVPP V12631 | 40.94 (right femur measured parallel to the femoral shaft between the top of the greater trochanter and the base of the lateral distal condyle) |  |  |
| *Meleagris gallopavo* (Turkey) | *Mele* | NHMUK 1898.5.30.1 | 124.94 (right) |  |  |
| *Pavo muticus* (Peacock) | *Pavo* | NHMUK 1847.12.11.13 | 104.38 (right) |  |  |
| *Struthio camelus* (Ostrich) | *Strut* | NHMUK S/2006.11.1 | 231 (left) |  |  |
| *Columba livia* (Pigeon) | *Colu* | UCL GMZ | 40.95 (right) |  |  |
| *Plateosaurus engelhardti* | *Plat* | MB.R. 4430 (Skel. C) | 673 (right) |  |  |
| *Crocodylus niloticus* (Nile crocodile) | *C.nil* | CMZ | 208 (left femur measured to the base of the lateral distal condyle) |  |  |
| *Crocodylus siamensis* (Siamese crocodile) | *C.sia* | X226 UCL GMZ | 104.80 (right femur measured to the base of the lateral distal condyle) |  |  |
| *Varanus niloticus* (Nile monitor) | *V.nil* | UCL GMZ | 62.94 (left femur measured to the base of the lateral distal condyle) |  |  |
| *Varanus komodoensis* (Komodo dragon) | *V.ko* | CMZ | 169 (right femur measured to the base of the lateral distal condyle) |  |  |
| *Acinonyx jubatus* (Cheetah) | *Acin* | CMZ K.5441 | 222 (right) |  |  |
| *Macropus giganteus* (Kangaroo) | *Macr* | CMZ A12.17/1 | 258 (left) |  |  |

Table S1. List of 31 coelurosaurian theropods (including birds) studied (and 7 outgroup taxa) and their associated femoral lengths in millimetres.

Table S2. Specimen housing institutions

| Abbreviation | Institution | Location |
| --- | --- | --- |
| AMNH | American Museum of Natural History | New York, USA |
| BMNHC | Beijing Museum of Natural History | Beijing, China |
| BSPG | Bayerische Staatssammlung für Paläontologie und Geologie | Munich, Germany |
| BYU | Brigham Young University | Provo, USA |
| CAGS | Chinese Academy of Geological Sciences | Beijing, China |
| D | Dalian Natural History Museum | Dalian, China |
| FMNH | Field Museum of Natural History | Chicago, USA |
| IVPP | Institute of Vertebrate Paleontology and Paleoanthropology | Beijing, China |
| JM/JME | Jura Museum | Eichstätt, Germany |
| MPC | Mongolian Paleontological Centre, Mongolian Academy of Sciences | Ulaanbaatar, Mongolia |
| MB.R/HMN | Museum für Naturkunde, Berlin | Berlin, Germany |
| MCZ | Museum of Comparative Zoology | Cambridge, USA |
| NIGP | Nanjing Institute of Geology and Palaeontology | Nanjing, China |
| NGMC | National Geological Museum of China | Beijing, China |
| OUMNH | Oxford University Museum of Natural History | Oxford, UK |
| TMP | Royal Tyrrell Museum of Palaeontology | Drumheller, Canada |
| STM | Shandong Tianyu Museum of Nature | Pingyi, China |
| NMNH | Smithsonian Institution  National Museum of Natural History | Washington D.C., USA |
| NHMUK | The Natural History Museum | London, UK |
| TNP | Tianjin Museum of Natural History | Tianjin, China |
| CMZ | University Museum of Zoology | Cambridge, UK |
| UCL GMZ | UCL Grant Museum of Zoology and Comparative Anatomy | London, UK |
| UCMP | University of California Museum of Paleontology | Berkeley, USA |
| YPM | Yale Peabody Museum | New Haven, USA |

Table S2. Abbreviations for the institutions visited as part of data collection and whose specimens have been discussed in the paper but from which data were not collected.

Table S3 (below) shows all of the size-normalised vertebral measurement data (all taxa studied and all vertebral parameters). Taxon abbreviations and the femoral lengths used to size-normalise the data can be found in Table S1. Zero values highlighted in bold font represent vertebral features that became absent along the tail (neural spines, transverse processes and chevrons). Caudal numbers highlighted in bold font formed the pygostyle. *Microraptor zhaoianus* data were from [2]. *Sinusonasus magnodens* had no femur preserved so the femur of close relative *Sinornithoides youngi* was used for size-normalisation instead.

Table S3. Complete dataset

| Vertebrae | | | Height normalised with femur length | | | Depth normalised with femur length | Length normalised with femur length | | | Width normalised with femur length |
| --- | --- | --- | --- | --- | --- | --- | --- | --- | --- | --- |
| Taxa | % of tail length | No. | Neural spine | Transverse process | Centrum | Chevron | Neural spine | Transverse process | Cent-rum | Vertebral |
| *Coel* | 1.94 | 1 |  |  | 0.108 |  |  | 0.0897 | 0.136 | 0.0832 |
| *Coel* | 4.18 | 2 |  | 0.178 | 0.103 |  |  | 0.0860 | 0.157 |  |
| *Coel* | 6.38 | 3 | 0.0473 | 0.211 | 0.0917 |  |  | 0.0506 | 0.155 |  |
| *Coel* | 8.56 | 4 |  | 0.215 | 0.0888 |  |  |  | 0.153 |  |
| *Coel* | 10.8 | 5 |  |  | 0.0973 |  |  |  | 0.155 |  |
| *Coel* | 13.1 | 6 |  |  | 0.0969 |  |  |  |  |  |
| *Coel* | 15.5 | 7 | 0.0615 | 0.0705 | 0.0830 |  | 0.0285 |  | 0.170 |  |
| *Coel* | 17.7 | 8 |  | 0.0796 | 0.0867 |  |  |  | 0.157 |  |
| *Coel* | 20.3 | 9 | 0.0630 |  | 0.0830 |  | 0.0442 | 0.0872 | 0.176 |  |
| *Coel* | 22.7 | 10 | 0.0613 | 0.0709 | 0.0825 |  | 0.0266 | 0.0591 | 0.172 |  |
| *Coel* | 25.0 | 11 | 0.0600 | 0.0674 | 0.0770 |  | 0.0197 | 0.0473 | 0.159 |  |
| *Coel* | 27.8 | 12 |  |  | 0.0952 |  |  |  | 0.199 |  |
| *Coel* | 30.0 | 13 |  | 0.0708 | 0.0948 |  |  | 0.0365 | 0.156 |  |
| *Coel* | 32.9 | 14 | 0.0749 | 0.0766 | 0.0872 |  | 0.0365 | 0.0629 | 0.202 |  |
| *Coel* | 35.5 | 15 | 0.0504 | 0.0600 | 0.0727 |  |  | 0.0703 | 0.185 |  |
| *Coel* | 38.8 | 16 |  | 0.0188 |  |  | 0.0192 | 0.0550 | 0.228 |  |
| *Coel* | 41.6 | 17 |  | 0.0340 |  |  | 0.0152 | 0.0616 | 0.195 |  |
| *Coel* | 44.2 | 18 |  | -0.00586 | 0.0774 |  |  | 0.0352 | 0.184 |  |
| *Coel* | 46.2 | 19 |  | **0** | 0.0593 |  |  | **0** | 0.140 |  |
| *Coel* | 48.3 | 20 |  | **0** | 0.0878 |  |  | **0** | 0.150 |  |
| *Coel* | 50.1 | 21 | 0.0187 | **0** | 0.0348 |  | 0.0839 | **0** | 0.125 |  |
| *Coel* | 53.3 | 22 | **0** | **0** | 0.0699 |  | **0** | **0** | 0.224 |  |
| *Coel* | 56.4 | 23 | **0** | **0** | 0.0596 |  | **0** | **0** | 0.218 |  |
| *Coel* | 59.5 | 24 | **0** | **0** | 0.0488 |  | **0** | **0** | 0.217 |  |
| *Coel* | 62.9 | 25 | **0** | **0** | 0.0428 |  | **0** | **0** | 0.239 |  |
| *Coel* | 66.3 | 26 | **0** | **0** | 0.0470 |  | **0** | **0** | 0.235 |  |
| *Coel* | 69.4 | 27 | **0** | **0** | 0.0458 |  | **0** | **0** | 0.217 |  |
| *Coel* | 72.4 | 28 | **0** | **0** | 0.0441 |  | **0** | **0** | 0.215 |  |
| *Coel* | 75.8 | 29 | **0** | **0** | 0.0394 |  | **0** | **0** | 0.237 |  |
| *Coel* | 79.0 | 30 | **0** | **0** | 0.0445 |  | **0** | **0** | 0.223 |  |
| *Coel* | 82.4 | 31 | **0** | **0** |  |  | **0** | **0** | 0.240 |  |
| *Coel* | 85.3 | 32 | **0** | **0** |  |  | **0** | **0** | 0.206 |  |
| *Coel* | 87.7 | 33 | **0** | **0** | 0.0302 |  | **0** | **0** | 0.166 |  |
| *Coel* | 90.7 | 34 | **0** | **0** | 0.0228 |  | **0** | **0** | 0.207 |  |
| *Coel* | 93.3 | 35 | **0** | **0** | 0.0242 |  | **0** | **0** |  |  |
| *Coel* | 95.6 | 36 | **0** | **0** | 0.0335 |  | **0** | **0** | 0.161 |  |
| *Coel* | 97.4 | 37 | **0** | **0** | 0.0257 |  | **0** | **0** | 0.130 |  |
| *Coel* | 98.8 | 38 | **0** | **0** | 0.0283 |  | **0** | **0** | 0.0940 |  |
| *Coel* | 100 | 39 | **0** | **0** | 0.0269 |  | **0** | **0** | 0.0868 |  |
| *Dilo* | 2.15 | 1 |  |  | 0.115 |  |  |  | 0.127 |  |
| *Dilo* | 4.34 | 2 | 0.146 | 0.115 | 0.102 | -0.187 |  | 0.0386 | 0.130 |  |
| *Dilo* | 6.66 | 3 |  |  |  |  |  |  |  |  |
| *Dilo* | 9.11 | 4 |  | 0.164 | 0.0869 | -0.152 |  | 0.0809 | 0.145 |  |
| *Dilo* | 11.5 | 5 |  |  | 0.0787 | -0.170 |  |  | 0.140 |  |
| *Dilo* | 13.9 | 6 |  | 0.0657 | 0.0705 | -0.170 |  |  | 0.142 |  |
| *Dilo* | 16.3 | 7 |  |  | 0.0695 | -0.162 |  | 0.0452 | 0.142 |  |
| *Dilo* | 18.7 | 8 |  |  | 0.0661 |  |  |  | 0.142 |  |
| *Dilo* | 21.0 | 9 |  |  | 0.0642 | -0.163 |  |  | 0.134 |  |
| *Dilo* | 23.3 | 10 |  |  | 0.0601 |  |  |  | 0.139 |  |
| *Dilo* | 25.7 | 11 | 0.0683 | 0.0343 | 0.0607 | -0.151 | 0.0257 |  | 0.142 |  |
| *Dilo* | 28.0 | 12 |  | 0.0342 | 0.0612 |  |  | 0.0219 | 0.137 |  |
| *Dilo* | 30.3 | 13 |  |  | 0.0577 |  |  |  | 0.135 |  |
| *Dilo* | 32.5 | 14 |  |  | 0.0635 |  |  |  |  |  |
| *Dilo* | 34.7 | 15 | 0.0781 | 0.0244 | 0.0567 |  | 0.0147 | 0.0262 | 0.130 |  |
| *Dilo* | 37.1 | 16 |  | 0.0200 | 0.0571 |  |  |  | 0.141 |  |
| *Dilo* | 39.5 | 17 |  |  | 0.0539 |  |  |  | 0.141 |  |
| *Dilo* | 42.0 | 18 |  | **0** | 0.0548 |  |  | **0** | 0.147 | 0.0519 |
| *Dilo* | 44.4 | 19 |  | **0** | 0.0543 |  |  | **0** | 0.145 | 0.0542 |
| *Dilo* | 46.8 | 20 | 0.0321 | **0** | 0.0528 |  | 0.00596 | **0** | 0.141 | 0.0548 |
| *Dilo* | 49.3 | 21 | 0.0396 | **0** | 0.0505 |  | 0.00715 | **0** | 0.146 | 0.0483 |
| *Dilo* | 51.7 | 22 |  | **0** | 0.0544 |  |  | **0** | 0.143 | 0.0496 |
| *Dilo* | 54.2 | 23 | 0.0396 | **0** | 0.0488 |  | 0.00755 | **0** | 0.144 | 0.0491 |
| *Dilo* | 56.5 | 24 |  | **0** | 0.0491 |  |  | **0** | 0.141 | 0.0477 |
| *Dilo* | 58.9 | 25 |  | **0** | 0.0444 |  |  | **0** | 0.140 | 0.0451 |
| *Dilo* | 61.3 | 26 |  | **0** | 0.0467 |  |  | **0** | 0.141 | 0.0513 |
| *Dilo* | 63.7 | 27 | **0** | **0** | 0.0461 |  | **0** | **0** | 0.141 | 0.0427 |
| *Dilo* | 66.0 | 28 | **0** | **0** | 0.0392 |  | **0** | **0** | 0.138 | 0.0392 |
| *Dilo* | 68.3 | 29 | **0** | **0** | 0.0355 |  | **0** | **0** | 0.136 | 0.0377 |
| *Dilo* | 70.6 | 30 | **0** | **0** | 0.0361 |  | **0** | **0** | 0.136 | 0.0386 |
| *Dilo* | 72.8 | 31 | **0** | **0** | 0.0302 |  | **0** | **0** | 0.132 | 0.0347 |
| *Dilo* | 75.0 | 32 | **0** | **0** | 0.0282 |  | **0** | **0** | 0.126 | 0.0306 |
| *Dilo* | 77.1 | 33 | **0** | **0** | 0.0259 |  | **0** | **0** | 0.126 |  |
| *Dilo* | 79.2 | 34 | **0** | **0** | 0.0247 |  | **0** | **0** | 0.125 | 0.0291 |
| *Dilo* | 81.3 | 35 | **0** | **0** |  |  | **0** | **0** |  |  |
| *Dilo* | 83.4 | 36 | **0** | **0** |  |  | **0** | **0** |  |  |
| *Dilo* | 85.5 | 37 | **0** | **0** | 0.0122 |  | **0** | **0** |  | 0.0158 |
| *Dilo* | 87.6 | 38 | **0** | **0** |  |  | **0** | **0** |  |  |
| *Dilo* | 89.7 | 39 | **0** | **0** | 0.0192 |  | **0** | **0** |  | 0.0218 |
| *Dilo* | 91.8 | 40 | **0** | **0** |  |  | **0** | **0** |  |  |
| *Dilo* | 93.8 | 41 | **0** | **0** |  |  | **0** | **0** |  |  |
| *Dilo* | 95.9 | 42 | **0** | **0** |  |  | **0** | **0** |  |  |
| *Dilo* | 98.0 | 42 | **0** | **0** |  |  | **0** | **0** |  |  |
| *Dilo* | 100 | 44 | **0** | **0** |  |  | **0** | **0** |  |  |
| *Trex* | 11.2 | 4 | 0.217 | 0.154 | 0.159 | -0.180 | 0.0871 | 0.0674 | 0.132 | 0.243 |
| *Trex* | 14.0 | 5 | 0.157 | 0.149 | 0.168 | -0.170 | 0.103 | 0.0643 | 0.141 | 0.249 |
| *Trex* | 16.8 | 6 | 0.183 | 0.133 | 0.148 | -0.138 | 0.0863 | 0.0725 | 0.143 | 0.269 |
| *Trex* | 19.7 | 7 | 0.188 | 0.135 | 0.138 | -0.138 | 0.0886 | 0.0765 | 0.142 | 0.245 |
| *Trex* | 22.5 | 8 | 0.182 | 0.105 | 0.129 | -0.129 | 0.0621 | 0.0553 | 0.139 | 0.228 |
| *Trex* | 25.6 | 9 | 0.162 | 0.0916 | 0.121 | -0.102 | 0.0772 | 0.0583 | 0.154 | 0.215 |
| *Trex* | 28.1 | 10 | 0.215 | 0.0844 | 0.117 | -0.114 | 0.0704 | 0.0553 | 0.124 | 0.218 |
| *Trex* | 30.8 | 11 | 0.148 | 0.0943 | 0.126 | -0.103 | 0.0833 | 0.0863 | 0.138 | 0.205 |
| *Trex* | 33.2 | 12 | 0.132 | 0.0742 | 0.118 | -0.111 | 0.0712 | 0.0409 | 0.120 | 0.207 |
| *Trex* | 36.1 | 13 | 0.132 | 0.0454 | 0.106 | -0.0780 | 0.0992 | 0.0409 | 0.142 | 0.185 |
| *Trex* | 39.1 | 14 | 0.0886 | 0.0477 | 0.106 | -0.0795 | 0.0810 | 0.0286 | 0.150 | 0.172 |
| *Trex* | 41.5 | 15 | 0.0939 | 0.0170 | 0.101 |  | 0.0174 | 0.0208 | 0.120 | 0.146 |
| *Trex* | 44.2 | 16 |  | 0.0220 | 0.095 |  | 0.0407 | 0.0150 | 0.138 | 0.127 |
| *Trex* | 47.1 | 17 | 0.0801 | 0.0316 | 0.088 |  | 0.0079 | 0.0118 | 0.145 | 0.0886 |
| *Trex* | 50.1 | 18 | 0.0795 | **0** | 0.089 | -0.0802 | 0.0165 | **0** | 0.145 | 0.102 |
| *Trex* | 52.9 | 19 | 0.0767 | **0** | 0.088 |  | 0.0136 | **0** | 0.144 | 0.0999 |
| *Trex* | 55.6 | 20 | 0.0648 | **0** | 0.0808 |  | 0.0232 | **0** | 0.131 | 0.0939 |
| *Trex* | 58.2 | 21 | 0.0621 | **0** | 0.0843 | -0.0428 | 0.00961 | **0** | 0.129 | 0.0848 |
| *Trex* | 60.6 | 22 | 0.0598 | **0** | 0.0848 | -0.0448 | 0.0112 | **0** | 0.122 | 0.0810 |
| *Trex* | 63.0 | 23 | 0.0514 | **0** | 0.0790 | -0.0475 | 0.0092 | **0** | 0.123 | 0.0780 |
| *Trex* | 65.3 | 24 | 0.0437 | **0** | 0.0749 | -0.0454 | 0.0148 | **0** | 0.112 | 0.0787 |
| *Trex* | 67.4 | 25 |  | **0** |  |  |  | **0** |  |  |
| *Trex* | 69.6 | 26 | 0.0453 | **0** | 0.0637 | -0.0490 | 0.0224 | **0** | 0.108 | 0.0802 |
| *Jura* | 3.01 | 2 |  |  |  |  |  | 0.0435 | 0.116 |  |
| *Jura* | 4.60 | 3 | 0.130 | 0.0770 | 0.0902 | -0.233 | 0.0445 | 0.0570 | 0.120 |  |
| *Jura* | 6.17 | 4 |  |  |  |  |  | 0.0546 | 0.119 |  |
| *Jura* | 7.85 | 5 | 0.151 |  | 0.0711 | -0.141 | 0.0704 | 0.0568 | 0.127 |  |
| *Jura* | 9.53 | 6 |  |  |  |  |  |  | 0.127 |  |
| *Jura* | 11.3 | 7 | 0.123 |  | 0.0792 | -0.164 |  |  | 0.131 |  |
| *Jura* | 12.9 | 8 | 0.110 |  | 0.0886 | -0.0882 |  |  | 0.124 |  |
| *Jura* | 14.7 | 9 |  |  | 0.0872 | -0.144 |  |  | 0.136 |  |
| *Jura* | 16.4 | 10 |  |  | 0.0872 | -0.125 |  |  | 0.126 |  |
| *Jura* | 18.2 | 11 |  |  | 0.0737 | -0.121 |  |  | 0.141 |  |
| *Jura* | 19.9 | 12 |  |  | 0.0889 | -0.122 |  |  | 0.128 |  |
| *Jura* | 21.8 | 13 | **0** |  | 0.105 |  | **0** |  | 0.147 |  |
| *Jura* | 23.6 | 14 | **0** |  | 0.0822 |  | **0** |  |  |  |
| *Jura* | 25.1 | 15 | **0** |  | 0.0591 |  | **0** |  | 0.113 |  |
| *Jura* | 26.9 | 16 | **0** |  | 0.0867 | -0.0869 | **0** |  | 0.138 |  |
| *Jura* | 28.9 | 17 | **0** |  | 0.0779 |  | **0** |  | 0.156 |  |
| *Jura* | 30.8 | 18 | **0** |  | 0.0773 | -0.0739 | **0** |  | 0.140 |  |
| *Jura* | 32.7 | 19 | **0** |  | 0.0685 | -0.0726 | **0** |  | 0.144 |  |
| *Jura* | 34.6 | 20 | **0** |  | 0.0664 | -0.0675 | **0** |  | 0.145 |  |
| *Jura* | 36.5 | 21 | **0** |  | 0.0687 | -0.0653 | **0** |  | 0.139 |  |
| *Jura* | 38.4 | 22 | **0** |  |  |  | **0** |  | 0.146 |  |
| *Jura* | 40.5 | 23 | **0** |  |  |  | **0** |  | 0.159 |  |
| *Jura* | 42.2 | 24 | **0** |  | 0.0630 |  | **0** |  | 0.132 |  |
| *Jura* | 44.2 | 25 | **0** |  | 0.0660 |  | **0** |  | 0.146 |  |
| *Jura* | 46.1 | 26 | **0** |  | 0.0583 |  | **0** |  |  |  |
| *Jura* | 47.9 | 27 | **0** |  | 0.0657 |  | **0** |  | 0.142 |  |
| *Jura* | 49.9 | 28 | **0** |  | 0.0698 |  | **0** |  | 0.147 |  |
| *Jura* | 51.9 | 29 | **0** |  |  |  | **0** |  | 0.153 |  |
| *Jura* | 53.8 | 30 | **0** |  |  |  | **0** |  | 0.147 |  |
| *Jura* | 55.9 | 31 | **0** |  | 0.0512 |  | **0** |  | 0.158 |  |
| *Jura* | 58.0 | 32 | **0** |  |  |  | **0** |  | 0.158 |  |
| *Jura* | 60.1 | 33 | **0** |  |  |  | **0** |  | 0.158 |  |
| *Jura* | 62.1 | 34 | **0** |  | 0.0662 |  | **0** |  | 0.148 |  |
| *Jura* | 64.1 | 35 | **0** |  | 0.0664 |  | **0** |  |  |  |
| *Jura* | 66.2 | 36 | **0** |  | 0.0632 |  | **0** |  | 0.161 |  |
| *Jura* | 68.3 | 37 | **0** |  | 0.0563 |  | **0** |  | 0.156 |  |
| *Jura* | 70.3 | 38 | **0** |  | 0.0612 |  | **0** |  | 0.152 |  |
| *Jura* | 72.3 | 39 | **0** |  | 0.0645 |  | **0** |  | 0.151 |  |
| *Jura* | 74.3 | 40 | **0** |  | 0.0574 |  | **0** |  | 0.149 |  |
| *Jura* | 76.2 | 41 | **0** |  | 0.0570 |  | **0** |  | 0.145 |  |
| *Jura* | 78.1 | 42 | **0** |  | 0.0574 |  | **0** |  | 0.145 |  |
| *Jura* | 80.1 | 43 | **0** |  | 0.0597 |  | **0** |  | 0.154 |  |
| *Comp* | 3.01 | 2 |  |  | 0.0593 | -0.168 |  |  |  |  |
| *Comp* | 4.60 | 3 | 0.160 |  | 0.0609 |  | 0.100 |  | 0.138 |  |
| *Comp* | 6.17 | 4 | 0.155 |  | 0.0663 |  | 0.103 |  | 0.144 |  |
| *Comp* | 7.85 | 5 | 0.153 |  | 0.0693 | -0.182 | 0.0875 |  | 0.149 |  |
| *Comp* | 9.53 | 6 |  |  |  |  | 0.101 |  |  |  |
| *Comp* | 11.3 | 7 |  |  |  | -0.113 | 0.107 |  |  |  |
| *Comp* | 12.9 | 8 |  |  |  |  | 0.0484 |  |  |  |
| *Comp* | 14.7 | 9 | 0.0853 |  | 0.0500 |  | 0.0232 |  | 0.168 |  |
| *Comp* | 16.4 | 10 | 0.0480 |  | 0.0582 |  | 0.0241 |  |  |  |
| *Ornit* | 1.77 | 1 |  | 0.0896 | 0.117 |  |  |  | 0.112 |  |
| *Ornit* | 4.00 | 2 |  |  | 0.103 |  |  |  | 0.142 |  |
| *Ornit* | 6.31 | 3 |  |  | 0.101 |  |  |  | 0.146 |  |
| *Ornit* | 8.65 | 4 |  |  | 0.0953 |  |  |  | 0.148 |  |
| *Ornit* | 11.0 | 5 |  |  | 0.0904 |  |  |  | 0.150 |  |
| *Ornit* | 13.4 | 6 |  |  | 0.0844 |  |  |  | 0.151 |  |
| *Ornit* | 15.9 | 7 |  |  | 0.0803 |  |  |  | 0.160 |  |
| *Ornit* | 18.5 | 8 |  |  |  |  |  |  |  |  |
| *Ornit* | 21.1 | 9 |  |  |  |  |  |  |  |  |
| *Ornit* | 23.7 | 10 |  |  |  |  |  |  |  |  |
| *Ornit* | 26.4 | 11 |  |  |  |  |  |  |  |  |
| *Ornit* | 29.2 | 12 |  |  |  |  |  |  |  |  |
| *Ornit* | 32.0 | 13 |  | 0.0378 | 0.0584 |  |  | 0.0151 | 0.177 |  |
| *Ornit* | 34.9 | 14 |  | **0** | 0.0593 |  |  | **0** | 0.181 | 0.0721 |
| *Ornit* | 37.6 | 15 |  | **0** | 0.0584 |  |  | **0** | 0.172 | 0.0723 |
| *Ornit* | 40.3 | 16 |  | **0** | 0.0591 |  |  | **0** | 0.176 | 0.0740 |
| *Ornit* | 43.1 | 17 | 0.0352 | **0** | 0.0574 | -0.0251 | 0.166 | **0** | 0.175 | 0.0747 |
| *Ornit* | 46.0 | 18 |  | **0** | 0.0556 |  |  | **0** | 0.181 | 0.0724 |
| *Ornit* | 48.9 | 19 |  | **0** | 0.0542 |  |  | **0** | 0.183 | 0.0789 |
| *Ornit* | 51.7 | 20 |  | **0** | 0.0501 |  |  | **0** | 0.178 | 0.0633 |
| *Ornit* | 54.4 | 21 | 0.0281 | **0** | 0.0453 | -0.0227 | 0.103 | **0** | 0.171 | 0.0598 |
| *Ornit* | 57.2 | 22 |  | **0** | 0.0432 |  |  | **0** | 0.179 | 0.0586 |
| *Ornit* | 60.0 | 23 |  | **0** | 0.0392 | -0.0152 |  | **0** | 0.177 | 0.0550 |
| *Ornit* | 63.1 | 24 |  | **0** |  |  |  | **0** | 0.201 | 0.0418 |
| *Ornit* | 66.1 | 25 |  | **0** | 0.0166 |  |  | **0** |  | 0.0503 |
| *Ornit* | 68.8 | 26 |  | **0** | 0.0173 |  |  | **0** | 0.173 | 0.0444 |
| *Ornit* | 71.6 | 27 |  | **0** | 0.0173 |  |  | **0** | 0.178 | 0.0438 |
| *Ornit* | 74.3 | 28 |  | **0** | 0.0139 |  |  | **0** | 0.170 | 0.0337 |
| *Ornit* | 76.7 | 29 |  | **0** | 0.0156 |  |  | **0** | 0.151 | 0.0385 |
| *Ornit* | 79.0 | 30 |  | **0** | 0.0168 |  |  | **0** | 0.145 | 0.0326 |
| *Shuv* | 3.39 | 2 | 0.0444 |  | 0.112 |  | 0.0302 |  |  |  |
| *Shuv* | 5.81 | 3 | 0.0800 | 0.0664 | 0.0859 |  | 0.0303 |  | 0.121 |  |
| *Shuv* | 8.82 | 4 | 0.0459 | 0.0485 | 0.0641 |  | 0.0486 |  | 0.150 |  |
| *Shuv* | 11.3 | 5 | 0.0701 | 0.0720 | 0.0727 | -0.0673 | 0.0403 |  | 0.125 |  |
| *Shuv* | 14.0 | 6 | 0.0753 |  | 0.0765 | -0.0576 | 0.0506 |  | 0.132 |  |
| *Shuv* | 18.4 | 7 |  | 0.0312 | 0.0773 |  |  |  | 0.222 |  |
| *Shuv* | 23.2 | 8 | 0.0407 | 0.0478 | 0.0598 | -0.0561 | 0.0452 |  | 0.236 |  |
| *Shuv* | 26.6 | 9 | 0.0703 | 0.0683 | 0.0584 | -0.0345 | 0.0246 |  | 0.170 |  |
| *Shuv* | 29.9 | 10 | 0.135 | 0.0455 | 0.0617 |  | 0.0180 | 0.0405 | 0.167 |  |
| *Shuv* | 33.3 | 11 | 0.0499 | 0.0451 | 0.0557 |  |  | 0.0432 | 0.170 |  |
| *Shuv* | 36.7 | 12 | 0.0579 | 0.0513 | 0.0555 |  | 0.0363 |  | 0.167 |  |
| *Shuv* | 40.1 | 13 | 0.0580 | 0.0362 | 0.0452 |  | 0.0219 | 0.0292 | 0.169 | 0.0637 |
| *Shuv* | 43.4 | 14 | 0.0431 | **0** | 0.0582 |  | 0.0403 |  | **0** | 0.0681 |
| *Citi* | 3.65 | 1 |  |  |  |  |  | 0.0403 |  | 0.256 |
| *Citi* | 7.29 | 2 |  |  |  | -0.0878 |  | 0.0392 |  |  |
| *Citi* | 10.9 | 3 |  |  |  |  |  | 0.0368 |  | 0.331 |
| *Citi* | 14.6 | 4 |  |  |  |  |  | 0.0294 |  | 0.318 |
| *Citi* | 18.2 | 5 |  |  |  |  |  | 0.0321 |  | 0.325 |
| *Citi* | 21.9 | 6 |  |  |  |  |  |  |  |  |
| *Citi* | 25.5 | 7 |  |  |  |  |  | 0.0324 |  | 0.296 |
| *Citi* | 29.2 | 8 |  |  |  |  |  | 0.0380 |  |  |
| *Citi* | 32.8 | 9 |  |  |  |  |  | 0.0408 |  | 0.263 |
| *Citi* | 36.5 | 10 |  |  |  |  |  |  |  |  |
| *Citi* | 40.1 | 11 | 0.0807 | 0.00595 | 0.0432 | -0.125 | 0.0263 | 0.0537 |  | 0.226 |
| *Citi* | 43.8 | 12 | 0.0662 | 0.00704 | 0.0440 | -0.114 | 0.0291 | 0.0646 |  |  |
| *Citi* | 47.4 | 13 |  | -0.00905 | 0.0440 | -0.104 |  | 0.0653 | 0.0908 | 0.204 |
| *Citi* | 51.1 | 14 |  | 0.00701 | 0.0428 | -0.0853 |  | 0.0615 |  | 0.189 |
| *Citi* | 54.4 | 15 | 0.0477 | 0.00293 | 0.0432 | -0.0762 | 0.0331 | 0.0584 |  | 0.178 |
| *Citi* | 57.8 | 16 |  |  |  | -0.0750 |  | 0.0615 | 0.0837 | 0.158 |
| *Citi* | 61.1 | 17 | 0.0389 | -0.00682 | 0.0368 | -0.0493 | 0.0358 | 0.0608 | 0.0836 | 0.159 |
| *Citi* | 64.5 | 18 |  |  |  | -0.0622 | 0.0285 | 0.0639 |  | 0.147 |
| *Citi* | 67.7 | 19 |  |  |  | -0.0482 | 0.0294 | 0.0563 |  | 0.129 |
| *Citi* | 70.9 | 20 | 0.0287 | -0.00528 | 0.0283 | -0.0272 | 0.0401 | 0.0619 |  | 0.120 |
| *Citi* | 74.1 | 21 | 0.0243 | -0.00307 | 0.0283 | -0.0166 | 0.0408 | 0.0614 | 0.0787 | 0.0981 |
| *Citi* | 77.1 | 22 | 0.0231 | 0.00352 | 0.0315 | -0.0280 | 0.0339 | 0.0498 | 0.0762 | 0.0845 |
| *Citi* | 80.0 | 23 | 0.0219 | 0.00402 | 0.0235 | -0.0181 | 0.0456 | 0.0494 | 0.0729 | 0.0704 |
| *Citi* | 82.7 | 24 | 0.0174 | 0.0025 | 0.0258 | -0.0193 | 0.0509 | 0.0436 | 0.0672 | 0.0575 |
| *Citi* | 85.4 | 25 | 0.0167 | 0.00416 | 0.0241 | -0.00880 | 0.0510 | 0.0367 | 0.0671 | 0.0491 |
| *Citi* | 88.0 | 26 | 0.0164 | -0.00056 | 0.0230 | -0.0137 | 0.0486 | 0.0313 | 0.0631 | 0.0423 |
| *Citi* | 90.4 | 27 | 0.0131 | 0.0027 | 0.0210 | -0.0100 | 0.0362 | 0.0225 | 0.0597 | 0.0343 |
| *Citi* | 92.6 | 28 | 0.00662 | 0.00098 | 0.0239 | -0.0107 | 0.0209 | 0.0133 | 0.0563 | 0.0268 |
| *Citi* | 94.7 | 29 | **0** | 0.0018 | 0.0196 | -0.00570 | **0** | 0.00654 | 0.0528 | 0.0169 |
| *Citi* | 96.8 | **30** | **0** | **0** | 0.0131 | -0.0102 | **0** | **0** | 0.0519 | 0.0122 |
| *Citi* | 98.4 | **31** | **0** | **0** | 0.00888 | **0** | **0** | **0** | 0.0382 | 0.00785 |
| *Citi* | 100 | **32** | **0** | **0** | 0.00682 | **0** | **0** | **0** | 0.0410 | 0.00444 |
| *Khaa* | 4.38 | 1 | 0.125 | 0.0223 | 0.0737 |  | 0.0277 | 0.0183 | 0.0820 |  |
| *Khaa* | 8.71 | 2 |  |  |  |  | 0.0301 | 0.0292 |  |  |
| *Khaa* | 13.0 | 3 |  |  |  |  | 0.0380 | 0.0387 |  | 0.351 |
| *Khaa* | 17.2 | 4 |  |  |  |  | 0.0333 | 0.0397 |  | 0.308 |
| *Khaa* | 21.4 | 5 |  |  |  |  | 0.0194 | 0.0401 |  | 0.305 |
| *Khaa* | 25.5 | 6 |  |  |  |  | 0.0124 | 0.0410 |  | 0.276 |
| *Khaa* | 29.6 | 7 |  |  |  |  |  | 0.0405 |  | 0.258 |
| *Khaa* | 33.6 | 8 |  |  |  |  |  | 0.0466 |  | 0.270 |
| *Khaa* | 37.6 | 9 |  |  |  |  |  | 0.0510 |  |  |
| *Khaa* | 41.5 | 10 |  |  |  |  | 0.0131 | 0.0370 |  | 0.233 |
| *Khaa* | 45.3 | 11 |  |  |  |  |  | 0.0392 |  | 0.230 |
| *Khaa* | 49.1 | 12 |  |  |  |  |  | 0.0387 |  |  |
| *Khaa* | 52.9 | 13 |  |  |  |  | 0.0214 |  |  |  |
| *Khaa* | 56.6 | 14 |  |  |  |  |  |  |  |  |
| *Khaa* | 60.3 | 15 | 0.0214 | -0.00746 | 0.0212 |  | 0.0262 | 0.0476 |  |  |
| *Khaa* | 63.9 | 16 | 0.0275 | -0.0107 | 0.0163 |  | 0.0329 | 0.0553 |  |  |
| *Khaa* | 67.4 | 17 | 0.0179 | -0.00931 | 0.0178 |  | 0.0349 | 0.0611 |  |  |
| *Khaa* | 70.9 | 18 | 0.0250 | -0.0103 | 0.0202 |  | 0.0343 | 0.0476 |  |  |
| *Khaa* | 74.3 | 19 | 0.0247 | -0.0187 | 0.0182 |  | 0.0195 | 0.0553 |  |  |
| *Khaa* | 77.7 | 20 | 0.0125 | -0.0118 | 0.0189 |  | 0.0153 | 0.0444 |  |  |
| *Khaa* | 81.1 | 21 |  |  |  |  |  | 0.0439 | 0.0625 | 0.0615 |
| *Khaa* | 84.2 | 22 | 0.0359 | -0.0191 | 0.0221 |  | 0.0081 | 0.0118 | 0.0590 |  |
| *Khaa* | 87.0 | 23 | 0.0274 | -0.0122 | 0.0194 |  | 0.0119 | 0.0040 | 0.0522 |  |
| *Khaa* | 90.3 | 24 | 0.0300 | -0.0029 | 0.0202 |  | 0.0150 | 0.0115 | 0.0622 |  |
| *Khaa* | 93.6 | 25 |  |  |  |  |  |  |  |  |
| *Khaa* | 96.8 | 26 | 0.0117 | **0** | 0.0225 |  | 0.0124 | **0** |  |  |
| *Khaa* | 100 | 27 | 0.00571 | **0** | 0.0260 |  | 0.00836 | **0** |  |  |
| *Caud* | 3.68 | 1 | 0.0673 |  | 0.0614 | -0.225 | 0.0315 |  | 0.0571 |  |
| *Caud* | 7.25 | 2 | 0.0743 |  | 0.0747 | -0.186 | 0.0368 |  | 0.0556 |  |
| *Caud* | 13.8 | 3 |  |  | 0.0570 |  |  |  | 0.102 |  |
| *Caud* | 20.5 | 4 |  |  |  | -0.183 |  |  |  |  |
| *Caud* | 27.2 | 5 | 0.0420 |  | 0.0419 | -0.133 | 0.0153 |  | 0.104 |  |
| *Caud* | 33.0 | 6 |  |  |  | -0.117 |  |  |  |  |
| *Caud* | 38.0 | 7 |  |  |  | -0.150 |  | 0.0255 | 0.0782 |  |
| *Caud* | 43.2 | 8 |  |  |  |  |  | 0.0189 | 0.0806 |  |
| *Caud* | 47.8 | 9 |  |  |  | -0.0831 |  |  | 0.0712 |  |
| *Caud* | 52.7 | 10 |  |  |  | -0.0884 |  | 0.0228 | 0.0763 |  |
| *Caud* | 56.7 | 11 | 0.0104 |  | 0.0628 | -0.0395 | 0.0148 |  | 0.0624 |  |
| *Caud* | 61.2 | 12 | 0.00800 |  | 0.0683 | -0.0281 | 0.0058 |  | 0.0688 |  |
| *Caud* | 65.9 | 13 | -0.000475 |  | 0.0663 |  | 0.0143 |  | 0.0729 |  |
| *Caud* | 68.8 | 14 | **0** |  | 0.0438 |  | **0** |  | 0.0455 |  |
| *Caud* | 73.0 | 15 | **0** |  |  |  | **0** |  | 0.0660 |  |
| *Caud* | 76.9 | 16 | **0** | **0** |  |  | **0** | **0** | 0.0601 | 0.0366 |
| *Caud* | 80.8 | 17 | **0** | **0** |  |  | **0** | **0** | 0.0602 | 0.0336 |
| *Caud* | 85.0 | 18 | **0** | **0** |  |  | **0** | **0** | 0.0662 | 0.0349 |
| *Caud* | 88.2 | 19 | **0** | **0** | 0.0323 |  | **0** | **0** | 0.0491 |  |
| *Caud* | 91.8 | 20 | **0** | **0** |  |  | **0** | **0** | 0.0557 |  |
| *Caud* | 94.9 | 21 | **0** | **0** |  | -0.0120 | **0** | **0** | 0.0483 |  |
| *Caud* | 97.8 | 22 | **0** | **0** |  |  | **0** | **0** | 0.0445 | 0.0138 |
| *Caud* | 99.2 | 23 | **0** | **0** | 0.0152 |  | **0** | **0** | 0.0229 |  |
| *Caud* | 100 | 24 | **0** | **0** | 0.0057 |  | **0** | **0** | 0.0121 |  |
| *Mei* | 5.26 | 3 | 0.0338 | 0.0203 | 0.0406 |  |  |  | 0.0571 |  |
| *Mei* | 7.02 | 4 |  |  |  |  | 0.0334 |  |  |  |
| *Mei* | 8.80 | 5 |  |  | 0.0375 | -0.0596 |  |  | 0.0579 |  |
| *Mei* | 10.5 | 6 | 0.0305 | 0.0172 | 0.0333 | -0.0527 |  | 0.0216 | 0.0558 |  |
| *Mei* | 12.5 | 7 | 0.0254 | 0.0376 | 0.0346 | -0.0406 | 0.0219 | 0.0219 | 0.0660 |  |
| *Mei* | 14.7 | 8 | 0.0259 | 0.0489 | 0.0417 |  |  | 0.0208 | 0.0709 |  |
| *Mei* | 17.6 | 9 | 0.0262 |  | 0.0322 | -0.0549 | 0.0798 | 0.0239 | 0.0944 | 0.113 |
| *Mei* | 21.9 | 10 | 0.0291 | 0.0079 | 0.0430 | -0.0256 | 0.101 | 0.0080 | 0.1411 |  |
| *Mei* | 27.5 | 11 | **0** | **0** | 0.0377 | -0.0224 | **0** | **0** | 0.183 | 0.0373 |
| *Mei* | 33.2 | 12 | **0** | **0** | 0.0364 | -0.0199 | **0** | **0** | 0.184 | 0.0451 |
| *Mei* | 38.9 | 13 | **0** | **0** | 0.0391 | -0.0316 | **0** | **0** | 0.185 | 0.0493 |
| *Mei* | 44.4 | 14 | **0** | **0** | 0.0406 | -0.0211 | **0** | **0** | 0.181 | 0.0477 |
| *Mei* | 50.3 | 15 | **0** | **0** | 0.0338 | -0.0186 | **0** | **0** | 0.192 | 0.0497 |
| *Mei* | 56.1 | 16 | **0** | **0** | 0.0341 | -0.0338 | **0** | **0** | 0.188 | 0.0354 |
| *Mei* | 61.7 | 17 | **0** | **0** | 0.0278 | -0.0353 | **0** | **0** | 0.182 | 0.0432 |
| *Mei* | 66.8 | 18 | **0** | **0** | 0.0338 | -0.0267 | **0** | **0** | 0.166 | 0.0443 |
| *Mei* | 72.1 | 19 | **0** | **0** | 0.024 | -0.0307 | **0** | **0** | 0.173 | 0.0393 |
| *Mei* | 77.2 | 20 | **0** | **0** | 0.0299 | -0.0181 | **0** | **0** | 0.169 | 0.0200 |
| *Mei* | 82.3 | 21 | **0** | **0** | 0.0262 | -0.0235 | **0** | **0** | 0.164 |  |
| *Mei* | 87.8 | 22 | **0** | **0** | 0.026 | -0.0245 | **0** | **0** | 0.180 | 0.0191 |
| *Mei* | 92.7 | 23 | **0** | **0** | 0.018 |  | **0** | **0** |  | 0.0228 |
| *Mei* | 97.0 | 24 | **0** | **0** | 0.018 |  | **0** | **0** |  | 0.0200 |
| *Mei* | 100 | 25 | **0** | **0** | 0.018 |  | **0** | **0** |  |  |
| *Sino* | 2.28 | 1 |  |  |  |  |  | 0.0297 | 0.0976 |  |
| *Sino* | 4.51 | 2 |  |  |  |  |  | 0.0379 | 0.0959 |  |
| *Sino* | 6.75 | 3 |  |  |  |  |  | 0.0377 |  |  |
| *Sino* | 8.99 | 4 |  |  |  |  |  | 0.0363 |  |  |
| *Sino* | 11.3 | 5 |  |  |  |  |  | 0.0403 |  |  |
| *Sino* | 13.6 | 6 |  |  |  |  |  | 0.0411 |  |  |
| *Sino* | 15.9 | 7 |  | 0.0300 | 0.0307 |  |  | 0.0311 | 0.0992 | 0.174 |
| *Sino* | 18.0 | 8 |  | 0.0293 | 0.0339 | -0.0625 |  | 0.0349 | 0.0878 |  |
| *Sino* | 20.7 | 9 |  | 0.0216 | 0.0334 | -0.0301 |  | 0.0265 | 0.115 |  |
| *Sino* | 24.6 | 10 | **0** | **0** | 0.0389 | -0.0185 | **0** | **0** | 0.169 |  |
| *Sino* | 28.8 | 11 | **0** | **0** | 0.0426 | -0.0165 | **0** | **0** | 0.181 |  |
| *Sino* | 33.0 | 12 | **0** | **0** | 0.0334 |  | **0** | **0** | 0.178 | 0.0385 |
| *Sino* | 37.5 | 13 | **0** | **0** | 0.0428 | -0.0145 | **0** | **0** | 0.195 |  |
| *Sino* | 42.1 | 14 | **0** | **0** | 0.0393 | -0.0137 | **0** | **0** | 0.195 |  |
| *Sino* | 46.3 | 15 | **0** | **0** | 0.0378 | -0.0165 | **0** | **0** | 0.183 | 0.0424 |
| *Sino* | 50.7 | 16 | **0** | **0** | 0.0349 | -0.0144 | **0** | **0** | 0.186 |  |
| *Sino* | 55.0 | 17 | **0** | **0** | 0.0458 | -0.0153 | **0** | **0** | 0.187 |  |
| *Sino* | 59.4 | 18 | **0** | **0** | 0.0337 | -0.00923 | **0** | **0** | 0.185 |  |
| *Sino* | 63.8 | 19 | **0** | **0** | 0.0304 | -0.00898 | **0** | **0** | 0.191 |  |
| *Sino* | 68.0 | 20 | **0** | **0** | 0.0242 | -0.0210 | **0** | **0** | 0.177 |  |
| *Sino* | 71.9 | 21 | **0** | **0** | 0.0331 | -0.0193 | **0** | **0** | 0.170 |  |
| *Sino* | 75.8 | 22 | **0** | **0** | 0.0248 | -0.0127 | **0** | **0** | 0.165 |  |
| *Sino* | 79.6 | 23 | **0** | **0** | 0.0317 | -0.0104 | **0** | **0** | 0.164 |  |
| *Sino* | 83.3 | 24 | **0** | **0** | 0.0247 | -0.0113 | **0** | **0** | 0.159 |  |
| *Sino* | 86.9 | 25 | **0** | **0** | 0.0208 | -0.00906 | **0** | **0** | 0.153 | 0.0211 |
| *Sino* | 90.0 | 26 | **0** | **0** | 0.0181 | -0.00604 | **0** | **0** | 0.135 | 0.0197 |
| *Sino* | 92.7 | 27 | **0** | **0** | 0.0210 |  | **0** | **0** |  | 0.0164 |
| *Sinu* | 2.47 | 1 |  |  |  |  |  |  | 0.110 |  |
| *Sinu* | 4.59 | 2 |  | 0.0558 | 0.0837 | -0.206 |  | 0.0523 | 0.0938 |  |
| *Sinu* | 6.89 | 3 |  | 0.0793 | 0.0676 |  |  | 0.0479 | 0.102 |  |
| *Sinu* | 9.09 | 4 |  |  |  |  |  |  |  |  |
| *Sinu* | 11.2 | 5 |  | 0.121 | 0.0742 |  |  | 0.0586 | 0.0931 |  |
| *Sinu* | 13.4 | 6 |  |  |  |  |  |  | 0.0982 |  |
| *Sinu* | 15.7 | 7 | **0** |  |  |  | **0** |  |  |  |
| *Sinu* | 18.1 | 8 | **0** | **0** | 0.0310 | -0.0847 | **0** | **0** | 0.105 | 0.213 |
| *Sinu* | 20.5 | 9 | **0** | **0** | 0.0293 | -0.0603 | **0** | **0** | 0.109 | 0.143 |
| *Sinu* | 23.1 | 10 | **0** | **0** | 0.0408 | -0.0643 | **0** | **0** | 0.116 |  |
| *Sinu* | 26.6 | 11 | **0** | **0** | 0.0415 | -0.0551 | **0** | **0** | 0.156 | 0.0457 |
| *Sinu* | 30.5 | 12 | **0** | **0** | 0.0429 | -0.0429 | **0** | **0** | 0.170 | 0.0469 |
| *Sinu* | 34.6 | 13 | **0** | **0** | 0.0419 | -0.0240 | **0** | **0** |  | 0.0476 |
| *Sinu* | 39.0 | 14 | **0** | **0** | 0.0446 | -0.0241 | **0** | **0** | 0.196 | 0.0490 |
| *Sinu* | 43.6 | 15 | **0** | **0** | 0.0381 | -0.0248 | **0** | **0** | 0.202 | 0.0490 |
| *Sinu* | 47.8 | 16 | **0** | **0** | 0.0392 | -0.0195 | **0** | **0** | 0.188 | 0.0514 |
| *Sinu* | 52.0 | 17 | **0** | **0** | 0.0427 | -0.0179 | **0** | **0** | 0.188 | 0.0459 |
| *Sinu* | 56.5 | 18 | **0** | **0** | 0.0478 | -0.0242 | **0** | **0** | 0.199 | 0.0494 |
| *Sinu* | 60.7 | 19 | **0** | **0** | 0.0446 |  | **0** | **0** | 0.185 | 0.0374 |
| *Sinu* | 64.8 | 20 | **0** | **0** | 0.0503 | -0.0298 | **0** | **0** | 0.182 | 0.0271 |
| *Sinu* | 69.1 | 21 | **0** | **0** | 0.0642 | -0.0192 | **0** | **0** | 0.193 | 0.0272 |
| *Sinu* | 73.2 | 22 | **0** | **0** | 0.0395 | -0.0196 | **0** | **0** | 0.183 | 0.0315 |
| *Sinu* | 77.2 | 23 | **0** | **0** | 0.0342 |  | **0** | **0** | 0.176 |  |
| *Sinu* | 81.3 | 24 | **0** | **0** | 0.0261 | -0.0184 | **0** | **0** | 0.180 | 0.0290 |
| *Sinu* | 85.0 | 25 | **0** | **0** | 0.0325 | -0.0142 | **0** | **0** | 0.168 | 0.0238 |
| *Sinu* | 88.5 | 26 | **0** | **0** | 0.0249 | -0.0276 | **0** | **0** | 0.152 | 0.0146 |
| *Sinu* | 91.9 | 27 | **0** | **0** | 0.0290 | -0.0252 | **0** | **0** | 0.153 | 0.0192 |
| *Sinu* | 95.1 | 28 | **0** | **0** | 0.0251 | -0.0138 | **0** | **0** | 0.142 | 0.0106 |
| *Sinu* | 97.8 | 29 | **0** | **0** | 0.0186 | -0.0108 | **0** | **0** | 0.120 | 0.0179 |
| *Maha* | 2.18 | 1 |  |  | 0.0588 |  |  |  | 0.134 |  |
| *Maha* | 4.43 | 2 |  |  |  |  |  |  |  |  |
| *Maha* | 6.77 | 3 |  | 0.0585 | 0.0552 |  | 0.0438 | 0.0357 | 0.144 |  |
| *Maha* | 8.91 | 4 |  | 0.0593 | 0.0463 |  | 0.0394 | 0.0424 | 0.132 |  |
| *Maha* | 11.3 | 5 | 0.0512 |  | 0.0547 |  | 0.0489 |  | 0.147 |  |
| *Maha* | 13.5 | 6 | 0.0411 | 0.0498 | 0.0511 |  | 0.0337 | 0.0335 | 0.137 |  |
| *Maha* | 16.0 | 7 | 0.0371 | 0.0390 | 0.0383 |  | 0.0316 | 0.0354 | 0.149 |  |
| *Maha* | 18.2 | 8 | 0.0519 | 0.0192 | 0.0470 |  | 0.0436 |  | 0.140 |  |
| *Maha* | 20.6 | 9 | 0.0461 | 0.0392 | 0.0478 |  | 0.0414 |  | 0.147 |  |
| *Maha* | 22.9 | 10 | 0.0415 | 0.0178 | 0.0496 | -0.0773 | 0.0322 | 0.0258 | 0.140 |  |
| *Maha* | 25.4 | 11 | 0.0428 | 0.0170 | 0.0463 |  | 0.0355 | 0.0325 | 0.154 |  |
| *Maha* | 28.1 | 12 | 0.0436 | 0.012 | 0.0432 |  | 0.0716 | 0.0129 | 0.162 | 0.0781 |
| *Maha* | 30.6 | 13 | 0.0410 | 0.0087 | 0.0365 |  |  |  | 0.155 | 0.0593 |
| *Maha* | 33.2 | 14 | 0.0363 | 0.0129 | 0.0480 | -0.0557 |  | 0.0286 | 0.163 | 0.0556 |
| *Maha* | 35.7 | 15 | 0.0273 | 0.0129 | 0.0422 |  |  | 0.0363 | 0.152 | 0.0440 |
| *Maha* | 38.2 | 16 | 0.0184 | **0** | 0.0468 | -0.0307 | 0.101 | **0** | 0.154 | 0.0273 |
| *Maha* | 40.8 | 17 | 0.0239 | **0** | 0.0429 |  | 0.103 | **0** | 0.158 | 0.0371 |
| *Maha* | 43.3 | 18 | 0.0162 | **0** | 0.0399 |  |  | **0** |  | 0.0392 |
| *Dein* | 18.8 | 7 |  | 0.0236 | 0.0704 |  |  |  | 0.128 | 0.0691 |
| *Dein* | 21.7 | 8 |  | 0.0378 | 0.0656 | -0.0618 |  |  | 0.136 | 0.0550 |
| *Dein* | 24.8 | 9 | 0.0496 |  | 0.0645 |  | 0.00923 |  | 0.148 | 0.0577 |
| *Dein* | 27.9 | 10 |  |  | 0.0714 |  |  |  |  | 0.0648 |
| *Dein* | 31.2 | 11 | 0.0363 | 0.0161 | 0.0661 | -0.0538 | 0.0912 | 0.0512 | 0.156 | 0.0618 |
| *Dein* | 34.5 | 12 | 0.0539 | **0** | 0.0457 |  | 0.136 | **0** | 0.156 | 0.0545 |
| *Dein* | 37.6 | 13 | 0.0481 | **0** | 0.0483 |  | 0.125 | **0** | 0.149 | 0.0470 |
| *Dein* | 40.7 | 14 |  | **0** | 0.0404 |  |  | **0** | 0.149 | 0.04622 |
| *Dein* | 43.8 | 15 | 0.0295 | **0** | 0.0529 | -0.0323 |  | **0** |  | 0.0510 |
| *Dein* | 46.8 | 16 | 0.0227 | **0** | 0.0545 |  |  | **0** | 0.146 | 0.0481 |
| *Dein* | 50.0 | 17 | 0.0376 | **0** | 0.0427 |  |  | **0** | 0.151 | 0.0470 |
| *Dein* | 52.9 | 18 | 0.0382 | **0** | 0.0406 | -0.0280 |  | **0** | 0.141 | 0.0479 |
| *Dein* | 56.0 | 19 | 0.0239 | **0** | 0.0506 | -0.0351 |  | **0** | 0.147 | 0.0449 |
| *Dein* | 59.0 | 20 | 0.0263 | **0** | 0.0438 | -0.0257 |  | **0** | 0.143 | 0.0405 |
| *Dein* | 61.9 | 21 | 0.0309 | **0** | 0.0377 | -0.0282 |  | **0** | 0.141 | 0.0419 |
| *Dein* | 64.9 | 22 | **0** | **0** | 0.0402 | -0.0248 | **0** | **0** | 0.142 | 0.0405 |
| *Dein* | 67.6 | 23 | **0** | **0** | 0.0293 | -0.0279 | **0** | **0** | 0.131 | 0.0362 |
| *Dein* | 70.1 | 24 | **0** | **0** | 0.0282 | -0.0528 | **0** | **0** | 0.118 | 0.0339 |
| *Dein* | 72.7 | 25 | **0** | **0** | 0.0301 |  | **0** | **0** | 0.124 | 0.0326 |
| *Dein* | 75.3 | 26 | **0** | **0** | 0.0229 |  | **0** | **0** | 0.123 | 0.0318 |
| *Dein* | 77.6 | 27 | **0** | **0** | 0.0196 |  | **0** | **0** |  | 0.0265 |
| *Dein* | 79.9 | 28 | **0** | **0** | 0.0212 | -0.0259 | **0** | **0** | 0.106 |  |
| *Dein* | 82.2 | 29 | **0** | **0** | 0.0193 |  | **0** | **0** | 0.112 |  |
| *Dein* | 84.1 | 30 | **0** | **0** | 0.0214 |  | **0** | **0** | 0.0895 |  |
| *Dein* | 86.0 | 31 | **0** | **0** | 0.0218 | -0.0231 | **0** | **0** | 0.0921 |  |
| *Dein* | 88.0 | 32 | **0** | **0** | 0.0174 |  | **0** | **0** | 0.0938 |  |
| *Dein* | 89.9 | 33 | **0** | **0** | 0.0161 |  | **0** | **0** | 0.0938 |  |
| *Dein* | 91.4 | 34 | **0** | **0** | 0.0133 |  | **0** | **0** | 0.0707 |  |
| *Dein* | 92.9 | 35 | **0** | **0** | 0.0119 |  | **0** | **0** | 0.0701 | 0.0116 |
| *Dein* | 94.3 | 36 | **0** | **0** | 0.0089 |  | **0** | **0** |  | 0.00949 |
| *Velo* | 1.93 | 1 |  |  |  |  |  | 0.0242 | 0.0890 | 0.238 |
| *Velo* | 3.96 | 2 | 0.134 | 0.0028 | 0.0901 |  |  | 0.0256 |  |  |
| *Velo* | 6.11 | 3 | 0.116 | 0.0244 | 0.0903 |  | 0.0507 |  |  | 0.299 |
| *Velo* | 8.85 | 4 | 0.114 | 0.0204 | 0.0830 |  | 0.0387 | 0.0493 | 0.127 | 0.310 |
| *Velo* | 11.8 | 5 |  | 0.0339 | 0.0803 | -0.0486 |  | 0.0439 | 0.134 | 0.300 |
| *Velo* | 14.1 | 6 |  |  |  |  |  |  |  |  |
| *Velo* | 16.0 | 7 |  | 0.0252 | 0.0735 | -0.0798 | 0.0390 | 0.0423 | 0.0858 | 0.269 |
| *Velo* | 19.2 | 8 | 0.0943 | 0.0168 | 0.0711 | -0.0735 |  | 0.00615 | 0.146 |  |
| *Velo* | 21.7 | 9 | 0.0674 | 0.0109 | 0.0753 | -0.0880 | 0.0205 | 0.0045 | 0.118 |  |
| *Velo* | 24.8 | 10 | 0.0664 | 0.0027 | 0.0651 | -0.0782 | 0.0288 | 0.0433 | 0.144 |  |
| *Velo* | 27.1 | 11 | 0.0597 | 0.00834 | 0.0528 | -0.0920 | 0.0215 | 0.0465 | 0.106 |  |
| *Velo* | 30.2 | 12 | 0.0450 | **0** | 0.0560 | -0.0758 | 0.0155 | **0** | 0.144 |  |
| *Velo* | 32.9 | 13 | 0.0405 | **0** | 0.0694 | -0.0560 | 0.0299 | **0** | 0.124 | 0.0732 |
| *Velo* | 35.6 | 14 | 0.0237 | **0** | 0.0639 | -0.0580 | 0.0359 | **0** | 0.123 |  |
| *Velo* | 38.2 | 15 |  | **0** |  |  |  | **0** |  |  |
| *Velo* | 41.3 | 16 |  | **0** |  |  |  | **0** |  |  |
| *Velo* | 44.4 | 17 | **0** | **0** | 0.0567 | -0.0498 | **0** | **0** | 0.141 |  |
| *Velo* | 47.4 | 18 | **0** | **0** |  |  | **0** | **0** |  |  |
| *Velo* | 49.8 | 19 | **0** | **0** |  |  | **0** | **0** |  |  |
| *Velo* | 52.2 | 20 | **0** | **0** |  |  | **0** | **0** | 0.110 | 0.0529 |
| *Velo* | 54.5 | 21 | **0** | **0** | 0.0487 | -0.0596 | **0** | **0** | 0.106 | 0.0632 |
| *Velo* | 56.9 | 22 | **0** | **0** | 0.0471 | -0.0420 | **0** | **0** | 0.111 | 0.0451 |
| *Velo* | 59.2 | 23 | **0** | **0** | 0.0364 |  | **0** | **0** | 0.109 | 0.0465 |
| *Velo* | 61.7 | 24 | **0** | **0** |  |  | **0** | **0** | 0.117 | 0.0443 |
| *Velo* | 64.1 | 25 | **0** | **0** | 0.0372 | -0.0234 | **0** | **0** | 0.111 | 0.0397 |
| *Velo* | 66.5 | 26 | **0** | **0** | 0.0410 |  | **0** | **0** |  | 0.0380 |
| *Vel*_F | 2.17 | 1 |  |  | 0.0667 | -0.257 |  |  | 0.107 |  |
| *Vel*_F | 4.56 | 2 |  |  | 0.0715 | -0.218 |  |  | 0.118 |  |
| *Vel*_F | 7.32 | 3 | 0.173 |  | 0.0757 |  |  | 0.0483 | 0.136 |  |
| *Vel*_F | 10.6 | 4 |  |  |  | -0.181 |  |  | 0.159 |  |
| *Vel*_F | 13.5 | 5 |  |  |  | -0.178 |  | 0.0689 | 0.143 |  |
| *Vel*_F | 17.2 | 6 |  |  | 0.0697 |  |  |  | 0.183 |  |
| *Vel*_F | 20.9 | 7 |  |  | 0.0794 |  |  |  | 0.184 |  |
| *Vel*_F | 24.3 | 8 |  |  |  |  |  |  | 0.169 |  |
| *Vel*_F | 27.8 | 9 |  |  |  | -0.104 |  |  | 0.171 |  |
| *Vel*_F | 30.7 | 10 |  |  |  |  |  |  | 0.143 |  |
| *Vel*_F | 33.7 | 11 |  |  |  |  |  |  | 0.147 |  |
| *Vel*_F | 37.3 | 12 |  | **0** |  |  |  | **0** | 0.178 |  |
| *Vel*_F | 41.1 | 13 |  | **0** |  | -0.0718 |  | **0** | 0.186 |  |
| *Vel*_F | 44.8 | 14 |  | **0** |  | -0.0624 |  | **0** | 0.186 |  |
| *Vel*_F | 48.5 | 15 |  | **0** |  | -0.0561 |  | **0** | 0.182 |  |
| *Vel*_F | 52.1 | 16 |  | **0** |  |  |  | **0** | 0.175 |  |
| *Vel*_F | 55.7 | 17 |  | **0** |  | -0.0500 |  | **0** | 0.177 |  |
| *Vel*_F | 59.2 | 18 |  | **0** |  |  |  | **0** | 0.174 |  |
| *Vel*_F | 62.5 | 19 |  | **0** |  |  |  | **0** | 0.164 |  |
| *Vel*_F | 66.0 | 20 |  | **0** |  |  |  | **0** | 0.169 |  |
| *Vel*_F | 69.0 | 21 |  | **0** |  |  |  | **0** | 0.149 |  |
| *Vel*_F | 72.8 | 22 |  | **0** |  |  |  | **0** | 0.189 |  |
| *Vel*_F | 76.2 | 23 |  | **0** |  |  |  | **0** | 0.170 |  |
| *Vel*_F | 80.0 | 24 |  | **0** |  |  |  | **0** | 0.183 |  |
| *Vel*_F | 83.2 | 25 |  | **0** |  |  |  | **0** | 0.162 |  |
| *Vel*_F | 86.0 | 26 |  | **0** |  | -0.0303 |  | **0** | 0.138 |  |
| *Vel*_F | 88.2 | 27 |  | **0** |  |  |  | **0** | 0.106 |  |
| *Vel*_F | 90.1 | 28 |  | **0** |  |  |  | **0** | 0.0950 |  |
| *Vel*_F | 92.7 | 29 |  | **0** |  |  |  | **0** | 0.127 |  |
| *Vel*_F | 93.4 | 30 |  | **0** |  |  |  | **0** | 0.0328 |  |
| *Bamb* | 0.97 | 1 | 0.132 | 0.0066 | 0.0494 |  | 0.0333 | 0.0338 | 0.0746 |  |
| *Bamb* | 2.21 | 2 | 0.120 | -0.0270 | 0.0377 |  | 0.0288 | 0.0457 | 0.0951 |  |
| *Bamb* | 3.64 | 3 | 0.123 | -0.0269 | 0.0568 |  | 0.0368 | 0.0573 | 0.110 |  |
| *Bamb* | 5.22 | 4 |  | 0.00898 | 0.0573 |  |  | 0.0543 | 0.122 |  |
| *Bamb* | 7.00 | 5 |  | 0.0202 | 0.0568 | -0.0465 |  | 0.0522 | 0.137 |  |
| *Bamb* | 8.85 | 6 | 0.0801 | 0.0105 | 0.0501 | -0.0707 | 0.0371 |  | 0.142 |  |
| *Bamb* | 10.7 | 7 |  | 0.0337 | 0.0466 |  |  | 0.0606 | 0.146 |  |
| *Bamb* | 12.9 | 8 |  | 0.0326 | 0.0513 | -0.0488 |  |  | 0.165 | 0.0594 |
| *Bamb* | 15.4 | 9 |  | 0.0249 | 0.0577 | -0.0327 |  | 0.0155 | 0.192 | 0.0492 |
| *Bamb* | 17.9 | 10 |  | 0.0210 | 0.0509 |  |  | 0.0619 | 0.189 | 0.0635 |
| *Bamb* | 20.3 | 11 |  | 0.0069 | 0.0427 | -0.0188 |  | 0.0985 | 0.188 |  |
| *Bamb* | 22.8 | 12 |  | 0.0169 | 0.0508 | -0.0125 |  | 0.166 | 0.195 |  |
| *Bamb* | 25.6 | 13 |  | **0** | 0.0491 |  |  | **0** | 0.212 |  |
| *Bamb* | 28.4 | 14 |  | **0** |  |  |  | **0** |  |  |
| *Bamb* | 31.1 | 15 |  | **0** |  |  |  | **0** |  |  |
| *Bamb* | 33.9 | 16 |  | **0** | 0.0466 |  |  | **0** |  |  |
| Drom | 10.7 | 2 |  |  |  |  |  | 0.0573 |  |  |
| Drom | 16.1 | 3 |  |  |  | -0.0774 |  | 0.0702 |  |  |
| Drom | 21.3 | 4 |  |  |  |  |  |  |  |  |
| Drom | 26.6 | 5 |  |  |  |  |  |  |  |  |
| Drom | 31.8 | 6 |  |  |  |  |  |  |  |  |
| Drom | 37.0 | 7 |  |  | 0.0922 |  |  |  | 0.568 |  |
| Drom | 42.1 | 8 |  |  | 0.0912 |  |  |  | 0.564 |  |
| Drom | 46.9 | 9 | **0** |  | 0.0822 |  | **0** |  | 0.532 |  |
| Drom | 51.4 | 10 | **0** |  | 0.0831 |  | **0** |  | 0.488 |  |
| Drom | 56.1 | 11 | **0** |  | 0.0917 |  | **0** |  | 0.520 |  |
| Drom | 60.8 | 12 | **0** |  | 0.0912 |  | **0** |  | 0.514 |  |
| Drom | 66.0 | 13 | **0** |  | 0.0936 |  | **0** |  | 0.576 |  |
| Drom | 70.9 | 14 | **0** |  |  |  | **0** |  | 0.543 |  |
| Drom | 75.4 | 15 | **0** |  |  |  | **0** |  | 0.492 |  |
| Drom | 79.5 | 16 | **0** |  |  |  | **0** |  |  |  |
| Drom | 83.7 | 17 | **0** |  |  |  | **0** |  |  |  |
| Drom | 86.7 | 18 | **0** |  |  |  | **0** |  |  |  |
| Drom | 89.2 | 19 | **0** |  |  |  | **0** |  |  |  |
| Drom | 91.8 | 20 | **0** |  |  |  | **0** |  |  |  |
| Drom | 94.5 | 21 | **0** |  |  |  | **0** |  |  |  |
| Drom | 96.7 | 22 | **0** |  |  |  | **0** |  |  |  |
| Drom | 98.6 | 23 | **0** |  |  |  | **0** |  |  |  |
| Drom | 100 | 24 | **0** |  | 0.0502 |  | **0** |  | 0.160 |  |
| *M.gui* | 5.63 | 1 |  |  | 0.0621 |  |  |  | 0.351 |  |
| *M.gui* | 10.4 | 2 |  |  |  |  |  |  | 0.295 |  |
| *M.gui* | 15.6 | 3 |  |  |  |  |  |  | 0.327 |  |
| *M.gui* | 20.4 | 4 |  |  | 0.0661 |  |  |  | 0.300 |  |
| *M.gui* | 25.4 | 5 |  |  |  |  |  |  | 0.312 |  |
| *M.gui* | 30.5 | 6 |  |  | 0.0668 |  |  |  | 0.321 |  |
| *M.gui* | 35.2 | 7 |  |  | 0.0625 |  |  |  | 0.289 |  |
| *M.gui* | 40.1 | 8 |  |  | 0.0640 |  |  |  | 0.306 |  |
| *M.gui* | 45.2 | 9 |  |  | 0.0500 |  |  |  | 0.317 |  |
| *M.gui* | 50.3 | 10 |  |  |  |  |  |  |  |  |
| *M.gui* | 55.6 | 11 |  |  | 0.0712 |  |  |  | 0.331 |  |
| *M.gui* | 60.3 | 12 |  |  | 0.0561 |  |  |  | 0.293 |  |
| *M.gui* | 64.7 | 13 |  |  |  |  |  |  | 0.276 |  |
| *M.gui* | 69.3 | 14 |  |  |  |  |  |  | 0.289 |  |
| *M.zha* | 1.77 | 1 |  |  |  |  |  |  | 0.0828 |  |
| *M.zha* | 3.92 | 2 |  |  |  |  |  |  | 0.101 |  |
| *M.zha* | 6.38 | 3 |  |  |  |  |  |  | 0.115 |  |
| *M.zha* | 10.1 | 4 |  |  |  |  |  |  | 0.176 |  |
| *M.zha* | 14.9 | 5 |  |  |  |  |  |  | 0.221 |  |
| *M.zha* | 20.4 | 6 |  |  |  |  |  |  | 0.260 |  |
| *M.zha* | 25.7 | 7 |  | **0** |  |  |  | **0** | 0.247 |  |
| *M.zha* | 30.8 | 8 |  | **0** |  |  |  | **0** | 0.240 |  |
| *M.zha* | 35.8 | 9 |  | **0** |  |  |  | **0** | 0.230 |  |
| *M.zha* | 40.7 | 10 |  | **0** |  |  |  | **0** | 0.231 |  |
| *M.zha* | 45.5 | 11 |  | **0** |  |  |  | **0** | 0.225 |  |
| *M.zha* | 50.5 | 12 |  | **0** |  |  |  | **0** | 0.231 |  |
| *M.zha* | 55.4 | 13 |  | **0** |  |  |  | **0** | 0.230 |  |
| *M.zha* | 60.0 | 14 |  | **0** |  |  |  | **0** | 0.218 |  |
| *M.zha* | 64.6 | 15 |  | **0** |  |  |  | **0** | 0.213 |  |
| *M.zha* | 69.2 | 16 |  | **0** |  |  |  | **0** | 0.219 |  |
| *M.zha* | 73.7 | 17 |  | **0** |  |  |  | **0** | 0.207 |  |
| *M.zha* | 77.7 | 18 |  | **0** |  |  |  | **0** | 0.188 |  |
| *M.zha* | 81.7 | 19 |  | **0** |  |  |  | **0** | 0.185 |  |
| *M.zha* | 85.3 | 20 |  | **0** |  |  |  | **0** | 0.168 |  |
| *M.zha* | 88.5 | 21 |  | **0** |  |  |  | **0** | 0.150 |  |
| *M.zha* | 91.5 | 22 |  | **0** |  |  |  | **0** | 0.142 |  |
| *M.zha* | 94.1 | 23 |  | **0** |  |  |  | **0** | 0.121 |  |
| *M.zha* | 96.2 | 24 |  | **0** |  |  |  | **0** | 0.100 |  |
| *M.zha* | 98.4 | 25 |  | **0** |  |  |  | **0** | 0.102 |  |
| *M.zha* | 100 | 26 |  | **0** |  |  |  | **0** | 0.0746 |  |
| *Epid* | 6.88 | 1 |  |  |  |  |  |  | 0.0948 |  |
| *Epid* | 11.4 | 2 |  |  |  |  |  |  | 0.0623 |  |
| *Epid* | 16.9 | 3 |  |  |  |  |  | 0.0288 | 0.0756 | 0.250 |
| *Epid* | 22.9 | 4 |  |  |  |  |  | 0.0476 |  |  |
| *Epid* | 28.1 | 5 |  |  |  |  |  |  | 0.0707 |  |
| *Epid* | 34.8 | 6 |  |  |  |  |  |  |  |  |
| *Epid* | 40.7 | 7 |  |  |  |  |  |  | 0.0818 |  |
| *Epid* | 49.0 | 8 |  |  |  |  |  |  | 0.115 |  |
| *Epid* | 58.0 | 9 |  |  |  |  |  |  | 0.124 |  |
| *Epid* | 67.4 | 10 |  |  |  |  |  |  | 0.129 |  |
| *Epid* | 75.7 | 11 |  |  |  |  |  |  | 0.115 |  |
| *Epid* | 83.4 | 12 |  |  |  |  |  |  | 0.107 |  |
| *Epid* | 89.3 | 13 |  |  |  |  |  |  | 0.0807 |  |
| *Epid* | 93.4 | 14 |  |  |  |  |  |  | 0.0572 |  |
| *Epid* | 96.9 | 15 |  |  |  |  |  |  | 0.0476 |  |
| *Epid* | 100 | 16 |  |  |  |  |  |  | 0.0428 |  |
| *Ar.*B | 1.84 | 1 |  |  |  |  | 0.017 | 0.0275 | 0.0607 |  |
| *Ar.*B | 3.95 | 2 |  |  |  |  | 0.020 |  | 0.0699 |  |
| *Ar.*B | 6.37 | 3 |  |  |  |  | 0.018 |  | 0.0800 |  |
| *Ar.*B | 9.42 | 4 | 0.0320 |  | 0.0468 | -0.115 |  |  | 0.101 |  |
| *Ar.*B | 12.9 | 5 | 0.0279 | 0.018 | 0.0506 |  |  |  | 0.114 |  |
| *Ar.*B | 17.6 | 6 |  |  | 0.0502 | -0.0217 |  |  | 0.155 |  |
| *Ar.*B | 23.2 | 7 |  | **0** | 0.0338 | -0.0193 |  | **0** | 0.185 |  |
| *Ar.*B | 29.6 | 8 |  | **0** |  |  |  | **0** | 0.212 |  |
| *Ar.*B | 36.9 | 9 |  | **0** |  |  |  | **0** | 0.212 |  |
| *Ar.*B | 42.4 | 10 |  | **0** | 0.0281 | -0.0223 |  | **0** | 0.214 |  |
| *Ar.*B | 49.2 | 11 |  | **0** | 0.0303 |  |  | **0** | 0.223 |  |
| *Ar.*B | 55.8 | 12 |  | **0** | 0.0297 |  |  | **0** | 0.219 |  |
| *Ar.*B | 62.1 | 13 |  | **0** |  |  |  | **0** | 0.209 |  |
| *Ar.*B | 68.6 | 14 |  | **0** | 0.0351 |  |  | **0** | 0.212 |  |
| *Ar.*B | 74.5 | 15 |  | **0** |  |  |  | **0** | 0.197 |  |
| *Ar.*B | 80.1 | 16 |  | **0** |  |  |  | **0** | 0.184 |  |
| *Ar.*J | 1.84 | 1 |  |  |  |  |  | 0.0321 | 0.0685 |  |
| *Ar.*J | 4.14 | 2 |  |  |  |  |  | 0.0402 | 0.0855 |  |
| *Ar.*J | 6.43 | 3 |  |  |  |  |  | 0.0483 | 0.0850 |  |
| *Ar.*J | 9.11 | 4 |  |  |  |  |  | 0.0515 |  |  |
| *Ar.*J | 12.2 | 5 | **0** |  | 0.025 | -0.0841 | **0** |  | 0.114 |  |
| *Ar.*J | 16.0 | 6 | **0** |  | 0.0472 | -0.0659 | **0** |  | 0.142 |  |
| *Ar.*J | 20.7 | 7 | **0** | 0.010 | 0.0456 | -0.0614 | **0** |  | 0.174 |  |
| *Ar.*J | 25.8 | 8 | **0** | 0.012 | 0.0485 | -0.0520 | **0** |  | 0.191 |  |
| *Ar.*J | 31.5 | 9 | **0** | **0** | 0.0512 | -0.0423 | **0** | **0** | 0.209 |  |
| *Ar.*J | 37.6 | 10 | **0** | **0** | 0.0534 | -0.0396 | **0** | **0** | 0.227 |  |
| *Ar.*J | 43.7 | 11 | **0** | **0** | 0.0426 | -0.0332 | **0** | **0** | 0.227 |  |
| *Ar.*J | 49.9 | 12 | **0** | **0** | 0.0418 | -0.0330 | **0** | **0** | 0.232 |  |
| *Ar.*J | 55.9 | 13 | **0** | **0** | 0.0394 | -0.0334 | **0** | **0** | 0.223 |  |
| *Ar.*J | 61.8 | 14 | **0** | **0** | 0.0405 | -0.022 | **0** | **0** | 0.220 |  |
| *Ar.*J | 67.8 | 15 | **0** | **0** | 0.0351 | -0.0305 | **0** | **0** | 0.221 |  |
| *Ar.*J | 73.4 | 16 | **0** | **0** | 0.0364 | -0.020 | **0** | **0** | 0.210 |  |
| *Ar.*J | 78.7 | 17 | **0** | **0** | 0.0442 |  | **0** | **0** | 0.197 |  |
| *Ar.*J | 83.7 | 18 | **0** | **0** | 0.0337 |  | **0** | **0** | 0.183 |  |
| *Ar.*J | 88.7 | 19 | **0** | **0** | 0.0351 |  | **0** | **0** | 0.189 |  |
| *Ar.*J | 93.1 | 20 | **0** | **0** |  |  | **0** | **0** | 0.162 | 0.022 |
| *Ar.*J | 96.3 | 21 | **0** | **0** |  |  | **0** | **0** | 0.120 | 0.019 |
| *Ar.*J | 98.6 | 22 | **0** | **0** |  |  | **0** | **0** | 0.0855 | 0.017 |
| *Ar.*J | 100 | 23 | **0** | **0** |  |  | **0** | **0** |  |  |
| *Ar.*H | 2.28 | 1 |  |  |  | -0.0892 |  | 0.0254 | 0.0761 |  |
| *Ar.*H | 4.86 | 2 |  |  |  | -0.0726 |  |  | 0.0864 |  |
| *Ar.*H | 7.68 | 3 |  |  |  | -0.0942 |  |  | 0.0942 |  |
| *Ar.*H | 11.4 | 4 |  |  |  |  |  | 0.0374 | 0.125 |  |
| *Ar.*H | 15.0 | 5 |  |  |  |  |  | 0.0440 | 0.119 |  |
| *Ar.*H | 19.4 | 6 | **0** | 0.00114 | 0.0496 | -0.0309 | **0** |  | 0.146 |  |
| *Ar.*H | 24.3 | 7 | **0** | 0.0036 | 0.0305 |  | **0** |  | 0.166 |  |
| *Ar.*H | 30.3 | 8 | **0** | 0.0082 | 0.0511 |  | **0** | 0.011 | 0.198 |  |
| *Ar.*H | 36.3 | 9 | **0** | **0** | 0.0425 | -0.016 | **0** | **0** | 0.201 |  |
| *Ar.*H | 42.3 | 10 | **0** | **0** | 0.0406 |  | **0** | **0** | 0.202 |  |
| *Ar.*H | 48.2 | 11 | **0** | **0** | 0.0406 |  | **0** | **0** | 0.198 |  |
| *Ar.*H | 55.9 | 12 | **0** | **0** | 0.0391 |  | **0** | **0** | 0.255 |  |
| *Ar.*H | 61.0 | 13 | **0** | **0** |  |  | **0** | **0** |  |  |
| *Ar.*H | 66.5 | 14 | **0** | **0** |  |  | **0** | **0** | 0.185 |  |
| *Ar.*H | 72.2 | 15 | **0** | **0** |  |  | **0** | **0** | 0.189 |  |
| *Ar.*H | 78.1 | 16 | **0** | **0** |  |  | **0** | **0** | 0.199 |  |
| *Ar.*H | 83.2 | 17 | **0** | **0** |  |  | **0** | **0** | 0.170 |  |
| *Ar.*H | 88.2 | 18 | **0** | **0** |  |  | **0** | **0** |  |  |
| *Ar.*H | 92.4 | 19 | **0** | **0** |  |  | **0** | **0** |  |  |
| *Ar.*H | 96.2 | 20 | **0** | **0** |  |  | **0** | **0** | 0.124 |  |
| *Ar.*H | 100 | 21 | **0** | **0** |  |  | **0** | **0** | 1.28 |  |
| *Jeho* | 2.22 | 1 |  |  |  |  |  | 0.0591 |  |  |
| *Jeho* | 5.25 | 2 |  |  |  |  | 0.0308 | 0.0308 | 0.152 | 0.0605 |
| *Jeho* | 9.48 | 3 | 0.0956 | 0.0005 | 0.0675 | -0.0497 |  | 0.0168 | 0.212 |  |
| *Jeho* | 14.8 | 4 |  | **0** | 0.0661 | -0.0329 |  | **0** | 0.266 |  |
| *Jeho* | 20.2 | 5 |  | **0** | 0.0604 |  |  | **0** | 0.271 |  |
| *Jeho* | 25.7 | 6 | **0** | **0** | 0.0600 |  | **0** | **0** | 0.275 |  |
| *Jeho* | 31.2 | 7 | **0** | **0** | 0.0615 | -0.0181 | **0** | **0** | 0.279 |  |
| *Jeho* | 36.6 | 8 | **0** | **0** | 0.0573 | -0.0244 | **0** | **0** | 0.266 |  |
| *Jeho* | 41.9 | 9 | **0** | **0** | 0.0511 | -0.0267 | **0** | **0** | 0.267 |  |
| *Jeho* | 47.0 | 10 | **0** | **0** | 0.0444 | -0.0219 | **0** | **0** | 0.259 |  |
| *Jeho* | 52.0 | 11 | **0** | **0** | 0.0467 | -0.0148 | **0** | **0** | 0.249 |  |
| *Jeho* | 57.2 | 12 | **0** | **0** | 0.0447 | -0.0163 | **0** | **0** | 0.259 |  |
| *Jeho* | 62.2 | 13 | **0** | **0** | 0.0365 | -0.0109 | **0** | **0** | 0.250 |  |
| *Jeho* | 67.0 | 14 | **0** | **0** |  |  | **0** | **0** |  |  |
| *Jeho* | 71.6 | 15 | **0** | **0** | 0.0363 | -0.0192 | **0** | **0** |  |  |
| *Jeho* | 76.2 | 16 | **0** | **0** | 0.0288 | -0.0069 | **0** | **0** | 0.221 |  |
| *Jeho* | 80.6 | 17 | **0** | **0** | 0.0360 | -0.0125 | **0** | **0** | 0.210 |  |
| *Jeho* | 84.7 | 18 | **0** | **0** | 0.0331 |  | **0** | **0** | 0.219 |  |
| *Jeho* | 89.1 | 19 | **0** | **0** | 0.0279 |  | **0** | **0** | 0.196 |  |
| *Jeho* | 93.0 | 20 | **0** | **0** | 0.0184 |  | **0** | **0** | 0.177 |  |
| *Jeho* | 96.6 | 21 | **0** | **0** | 0.0159 |  | **0** | **0** | 0.173 |  |
| *Jeho* | 100 | 22 | **0** | **0** | 0.012 |  | **0** | **0** | 0.152 | 0.012 |
| *Sape* | 9.55 | 1 |  |  |  |  | 0.013 | 0.0198 | 0.0674 |  |
| *Sape* | 18.6 | 2 |  |  |  |  |  | 0.0064 | 0.0637 |  |
| *Sape* | 23.9 | 3 |  |  |  |  |  | 0.0157 |  | 0.136 |
| *Sape* | 32.0 | 4 |  |  |  |  |  | 0.0223 | 0.0570 | 0.137 |
| *Sape* | 40.9 | 5 |  |  |  |  |  | 0.0233 | 0.0631 | 0.127 |
| *Sape* | 50.5 | 6 |  |  |  | -0.0416 |  | 0.0180 | 0.0675 | 0.124 |
| *Sape* | 58.6 | 7 |  |  |  |  |  | 0.0211 |  |  |
| *Sape* | 62.7 | 8 |  |  |  |  |  | 0.0146 |  | 0.120 |
| *Sape* | 67.9 | 9 |  |  |  |  |  | 0.0284 | 0.0366 | 0.142 |
| *Sape* | 72.9 | **10** |  |  |  |  |  | 0.0354 |  | 0.128 |
| *Sape* | 80.6 | **11** |  |  |  |  |  | 0.0160 | 0.0544 |  |
| *Sape* | 87.7 | **12** |  |  |  |  |  |  | 0.0505 |  |
| *Sape* | 94.0 | **13** |  |  |  |  |  |  | 0.0445 |  |
| *Sape* | 97.1 | **14** |  |  |  |  |  |  | 0.0219 |  |
| *Sape* | 100 | **15** |  |  |  |  |  |  | 0.0202 |  |
| *Conf* | 7.18 | 1 |  |  |  |  |  |  | 0.0656 |  |
| *Conf* | 15.4 | 2 |  |  |  |  |  | 0.016 | 0.0748 |  |
| *Conf* | 23.4 | 3 |  |  |  |  |  |  | 0.0732 |  |
| *Conf* | 29.9 | 4 |  |  |  |  |  |  | 0.0594 |  |
| *Conf* | 100 | **5** |  |  |  |  |  |  | 0.641 |  |
| *Hong* | 20.7 | 1 | 0.019 |  | 0.0582 | -0.033 | 0.014 |  | 0.0499 |  |
| *Hong* | 44.2 | 2 | 0.028 |  | 0.0568 |  | 0.0083 |  | 0.0568 |  |
| *Hong* | 57 | 3 |  |  | 0.037 |  |  |  | 0.030 |  |
| *Hong* | 100 | **4** |  |  |  |  |  |  | 0.105 |  |
| *Yixi* | 10.6 | 1 |  | 0.021 | 0.0650 |  |  | 0.019 | 0.0633 |  |
| *Yixi* | 23.4 | 2 | 0.0723 | 0.0299 | 0.0706 |  | 0.022 | 0.0274 | 0.0765 |  |
| *Yixi* | 32.1 | 3 |  |  |  |  |  |  |  |  |
| *Yixi* | 41.4 | 4 |  |  | 0.0628 |  |  |  |  |  |
| *Yixi* | 46.9 | 5 |  |  |  |  |  |  |  |  |
| *Yixi* | 56.4 | 6 |  |  | 0.0616 | -0.0308 |  |  |  |  |
| *Yixi* | 63.3 | 7 | 0.0447 |  | 0.0420 | -0.0532 | 0.022 |  | 0.0413 |  |
| *Yixi* | 71.6 | 8 | 0.0347 |  | 0.0457 | -0.0542 | 0.019 |  | 0.0498 |  |
| *Yixi* | 78.0 | **9** |  |  | 0.0276 | -0.0293 |  |  | 0.0383 |  |
| *Yixi* | 86.0 | **10** | 0.0398 |  | 0.0261 | -0.0081 |  |  | 0.0474 |  |
| *Yixi* | 93.4 | **11** | 0.0398 |  | 0.0271 | -0.016 | 0.021 |  | 0.0447 |  |
| *Yixi* | 97.8 | **12** | 0.018 |  | 0.0369 |  | 0.012 |  | 0.0259 |  |
| *Yixi* | 99 | **13** | 0.018 |  | 0.0337 |  | 0.0059 |  | 0.010 |  |
| *Yixi* | 100 | **14** | 0.015 |  | 0.0247 |  | 0.0056 |  | 0.0032 |  |
| *Mele* | 16.6 | 1 | 0.0532 | 0.0013 | 0.0371 | **0** | 0.0472 | 0.0812 | 0.0912 | 0.272 |
| *Mele* | 28.2 | 2 | 0.0620 | -0.0486 | 0.0527 | **0** | 0.0206 | 0.0541 | 0.0637 | 0.290 |
| *Mele* | 40.4 | 3 | 0.0620 | -0.0326 | 0.0491 | **0** | 0.0236 | 0.0621 | 0.0670 | 0.275 |
| *Mele* | 50.0 | 4 | 0.0620 | -0.0848 | 0.0465 | **0** | 0.0147 | 0.0434 | 0.0527 | 0.269 |
| *Mele* | 59.4 | 5 | 0.0620 | -0.0489 | 0.0419 | **0** | 0.0150 | 0.0531 | 0.0517 | 0.217 |
| *Mele* | 100 | **6** |  |  |  |  |  |  | 0.223 |  |
| *Pavo* | 12.1 | 1 | 0.0651 | -0.0321 | 0.0542 | **0** | 0.0261 | 0.0875 | 0.0751 | 0.309 |
| *Pavo* | 25.1 | 2 | 0.0744 | -0.0195 | 0.0560 | **0** | 0.0332 | 0.0876 | 0.0802 | 0.343 |
| *Pavo* | 38.4 | 3 | 0.0726 | -0.0490 | 0.0572 | **0** | 0.0401 | 0.0948 | 0.0823 | 0.391 |
| *Pavo* | 49.6 | 4 | 0.0722 | -0.0441 | 0.0522 | **0** | 0.0339 | 0.0908 | 0.0696 | 0.352 |
| *Pavo* | 100 | **5** |  |  |  |  |  |  | 0.312 |  |
| *Strut* | 12.4 | 1 | 0.105 | 0.0573 | 0.0685 | **0** | 0.0501 | 0.0375 | 0.0985 | 0.137 |
| *Strut* | 22.6 | 2 | 0.0874 | 0.0460 | 0.0718 | **0** | 0.0298 | 0.0214 | 0.0808 | 0.142 |
| *Strut* | 32.2 | 3 | 0.0769 | 0.0345 | 0.0631 | **0** | 0.0359 | 0.0298 | 0.0759 | 0.142 |
| *Strut* | 41.6 | 4 | 0.0714 | 0.0170 | 0.0599 | **0** | 0.0301 | 0.0255 | 0.0746 | 0.147 |
| *Strut* | 50.5 | 5 | 0.0636 | 0.00916 | 0.0563 | **0** | 0.0289 | 0.0237 | 0.0706 | 0.139 |
| *Strut* | 59.4 | 6 | 0.0505 | 0.0167 | 0.0591 | **0** | 0.0234 | 0.0241 | 0.0713 | 0.133 |
| *Strut* | 67.2 | 7 | 0.0501 | 0.0137 | 0.0522 | **0** | 0.0191 | 0.0207 | 0.0613 | 0.125 |
| *Strut* | 74.5 | 8 | 0.0456 | 0.00987 | 0.0497 | **0** | 0.0199 | 0.0252 | 0.0584 | 0.111 |
| *Strut* | 81.3 | 9 | 0.0519 | 0.00506 | 0.0451 | **0** | 0.0172 | 0.0129 | 0.0539 | 0.0783 |
| *Strut* | 100 | **10** |  |  |  |  |  |  | 0.148 |  |
| *Colu* | 13.0 | 1 | 0.0850 | -0.0474 | 0.0464 | **0** | 0.022 | 0.0669 | 0.0779 | 0.347 |
| *Colu* | 26.8 | 2 | 0.0984 | -0.0656 | 0.0476 | **0** | 0.0320 | 0.0420 | 0.0833 | 0.325 |
| *Colu* | 40.3 | 3 | 0.0930 | -0.0912 | 0.0369 | **0** | 0.0400 | 0.0618 | 0.0811 | 0.375 |
| *Colu* | 53.7 | 4 | 0.0728 | -0.133 | 0.0352 | **0** | 0.0330 | 0.0349 | 0.0803 |  |
| *Colu* | 68.2 | 5 | 0.115 | -0.101 | 0.0396 | **0** | 0.0374 | 0.0413 | 0.0869 |  |
| *Colu* | 78.0 | 6 | 0.0799 |  | 0.0481 | **0** | 0.0352 |  | 0.0589 |  |
| *Colu* | 100 | **7** |  |  |  |  |  |  | 0.132 |  |
| *Plat* | 2.79 | 1 |  | 0.10 | 0.147 |  |  | 0.0685 | 0.111 |  |
| *Plat* | 5.5 | 2 |  |  | 0.134 | -0.306 |  |  | 0.11 |  |
| *Plat* | 8.0 | 3 |  |  | 0.12 | -0.358 |  |  | 0.10 |  |
| *Plat* | 11 | 4 | 0.218 |  | 0.13 | -0.330 | 0.043 |  | 0.12 |  |
| *Plat* | 14 | 5 | 0.202 |  | 0.13 | -0.339 | 0.0462 |  | 0.13 |  |
| *Plat* | 17 | 6 | 0.166 |  | 0.11 | -0.239 | 0.0524 |  | 0.13 |  |
| *Plat* | 20 | 7 | 0.158 |  | 0.0901 |  |  | 0.0589 | 0.12 |  |
| *Plat* | 23 | 8 |  | 0.0890 | 0.0910 |  |  | 0.0411 | 0.12 |  |
| *Plat* | 26 | 9 |  | 0.0953 | 0.089 | -0.241 |  |  | 0.11 |  |
| *Plat* | 29 | 10 | 0.125 | 0.0857 | 0.0883 |  |  | 0.031 | 0.11 |  |
| *Plat* | 31 | 11 |  | 0.0635 | 0.0795 | -0.260 |  | 0.052 | 0.10 |  |
| *Plat* | 34.0 | 12 | 0.104 | 0.0246 | 0.0860 |  | 0.040 | 0.053 | 0.113 | 0.236 |
| *Plat* | 36.9 | 13 | 0.121 |  | 0.0704 | -0.241 | 0.0399 |  | 0.113 |  |
| *Plat* | 39.5 | 14 |  |  | 0.067 | -0.214 |  |  | 0.107 |  |
| *Plat* | 42.1 | 15 |  |  | 0.0739 | -0.183 |  |  | 0.101 |  |
| *Plat* | 44.6 | 16 | 0.102 | 0.0401 | 0.0719 | -0.184 | 0.0291 | 0.0165 | 0.0999 |  |
| *Plat* | 47.1 | 17 | 0.103 | 0.00958 | 0.0638 |  |  | 0.0227 | 0.101 |  |
| *Plat* | 49.6 | 18 | 0.103 |  | 0.0570 | -0.203 | 0.0233 |  | 0.0977 |  |
| *Plat* | 51.8 | 19 |  | 0.0129 | 0.0539 | -0.177 |  | 0.0195 | 0.0897 |  |
| *Plat* | 53.8 | 20 | 0.0870 | 0.00314 | 0.0568 | -0.174 | 0.0207 | 0.0132 | 0.0787 | 0.156 |
| *Plat* | 55.8 | 21 | 0.0832 | 0.0013 | 0.0492 | -0.151 | 0.0229 | 0.0107 | 0.0786 |  |
| *Plat* | 57.8 | 22 | 0.0807 | -0.0001 | 0.0485 | -0.140 | 0.0107 | 0.0103 | 0.0790 | 0.135 |
| *Plat* | 59.7 | 23 | 0.0660 | 0.00720 | 0.0511 | -0.135 | 0.0148 | 0.0154 | 0.0759 | 0.124 |
| *Plat* | 61.7 | 24 | 0.0773 | 0.00479 | 0.0520 | -0.127 | 0.0220 | 0.00944 | 0.0809 | 0.110 |
| *Plat* | 63.8 | 25 | 0.0723 | 0.00097 | 0.0448 | -0.116 | 0.0159 | 0.0100 | 0.0813 | 0.0943 |
| *Plat* | 65.7 | 26 |  | -0.00299 | 0.0424 | -0.104 |  | 0.0119 | 0.0764 | 0.0682 |
| *Plat* | 67.4 | 27 |  |  |  | -0.119 |  | 0.00935 | 0.0704 | 0.0747 |
| *Plat* | 69.0 | 28 |  |  |  | -0.106 |  | 0.00713 | 0.0616 |  |
| *Plat* | 70.3 | 29 |  | 0.00906 | 0.0303 | -0.102 |  | 0.00449 | 0.0527 |  |
| *Plat* | 71.9 | 30 |  |  | 0.0377 |  |  |  | 0.0628 |  |
| *Plat* | 73.6 | 31 |  |  |  | -0.0805 |  | 0.00587 | 0.0681 | 0.0337 |
| *Plat* | 75.3 | 32 |  | 0.0110 | 0.0385 |  |  | 0.00634 | 0.0675 | 0.0333 |
| *Plat* | 77.0 | 33 |  | **0** | 0.0348 | -0.0648 |  | **0** | 0.0666 | 0.0281 |
| *Plat* | 78.7 | 34 |  | **0** | 0.0306 | -0.0519 |  | **0** | 0.0672 | 0.0304 |
| *Plat* | 80.2 | 35 |  | **0** | 0.0278 | -0.0543 |  | **0** | 0.0636 | 0.022 |
| *Plat* | 81.8 | 36 |  | **0** | 0.0256 | -0.0493 |  | **0** | 0.0637 | 0.0265 |
| *Plat* | 83.4 | 37 |  | **0** | 0.0236 | -0.0406 |  | **0** | 0.0600 | 0.0212 |
| *Plat* | 84.8 | 38 | **0** | **0** | 0.0217 |  | **0** | **0** | 0.0570 | 0.0209 |
| *Plat* | 86.2 | 39 | **0** | **0** | 0.0193 | -0.0468 | **0** | **0** | 0.0552 | 0.0196 |
| *Plat* | 87.6 | 40 | **0** | **0** | 0.0222 |  | **0** | **0** | 0.0553 | 0.0182 |
| *Plat* | 89 | 41 | **0** | **0** | 0.0194 |  | **0** | **0** | 0.051 | 0.0184 |
| *Plat* | 90.1 | 42 | **0** | **0** | 0.0204 |  | **0** | **0** | 0.0489 | 0.0214 |
| *Plat* | 91.1 | 43 | **0** | **0** | 0.0165 | -0.0416 | **0** | **0** |  | 0.0188 |
| *Plat* | 92.4 | 44 | **0** | **0** |  |  | **0** | **0** | 0.0507 |  |
| *C.nil* | 2.18 | 1 | 0.270 | 0.0386 | 0.113 | **0** | 0.0720 | 0.0360 | 0.150 | 0.546 |
| *C.nil* | 4.76 | 2 | 0.270 | 0.0517 | 0.129 | **0** | 0.0774 | 0.0260 | 0.177 | 0.559 |
| *C.nil* | 7.41 | 3 | 0.278 | 0.0350 | 0.120 | -0.252 | 0.0785 | 0.0237 | 0.182 | 0.540 |
| *C.nil* | 10.1 | 4 | 0.279 | 0.0256 | 0.113 | -0.297 | 0.0738 | 0.0206 | 0.187 | 0.508 |
| *C.nil* | 13.0 | 5 | 0.279 | 0.0381 | 0.112 | -0.249 | 0.0731 | 0.0235 | 0.195 | 0.473 |
| *C.nil* | 15.8 | 6 | 0.282 | 0.0314 | 0.109 | -0.174 | 0.0668 | 0.0141 | 0.197 | 0.432 |
| *C.nil* | 18.7 | 7 | 0.270 | 0.0045 | 0.105 | -0.195 | 0.0701 | 0.0566 | 0.200 | 0.376 |
| *C.nil* | 21.7 | 8 |  | 0.0135 | 0.0997 | -0.200 |  | 0.0487 | 0.202 | 0.352 |
| *C.nil* | 24.6 | 9 | 0.278 | 0.0240 | 0.0965 | -0.206 | 0.0545 | 0.0454 | 0.199 | 0.333 |
| *C.nil* | 27.5 | 10 | 0.272 | 0.0388 | 0.0943 | -0.198 | 0.0503 | 0.0448 | 0.199 | 0.306 |
| *C.nil* | 30.4 | 11 | 0.268 | 0.0544 | 0.0907 | -0.163 | 0.0403 | 0.0350 | 0.202 | 0.297 |
| *C.nil* | 33.4 | 12 | 0.268 | 0.0574 | 0.0850 | -0.164 | 0.0318 | 0.0331 | 0.205 | 0.282 |
| *C.nil* | 36.4 | 13 | 0.240 | 0.0658 | 0.0852 | -0.132 | 0.0312 | 0.0273 | 0.202 | 0.262 |
| *C.nil* | 39.3 | 14 | 0.240 | 0.0374 | 0.0837 | -0.136 | 0.0478 | 0.0210 | 0.200 | 0.241 |
| *C.nil* | 42.2 | 15 | 0.272 | 0.0572 | 0.0828 | -0.136 | 0.0374 | 0.0202 | 0.199 | 0.204 |
| *C.nil* | 45.0 | 16 | 0.282 | **0** | 0.0851 | -0.121 | 0.0303 | **0** | 0.197 | 0.0974 |
| *C.nil* | 47.9 | 17 |  | **0** | 0.0843 | -0.120 |  | **0** | 0.196 | 0.0920 |
| *C.nil* | 50.9 | 18 |  | **0** | 0.0795 | -0.117 |  | **0** | 0.207 | 0.0883 |
| *C.nil* | 53.9 | 19 | 0.259 | **0** | 0.0758 | -0.108 | 0.0278 | **0** | 0.205 | 0.0901 |
| *C.nil* | 56.9 | 20 |  | **0** | 0.0716 | -0.104 |  | **0** | 0.207 | 0.0841 |
| *C.nil* | 59.8 | 21 |  | **0** | 0.0649 | -0.100 |  | **0** | 0.201 | 0.0828 |
| *C.nil* | 62.8 | 22 | 0.192 | **0** | 0.0644 | -0.0878 | 0.0264 | **0** | 0.201 | 0.0783 |
| *C.nil* | 65.6 | 23 | 0.187 | **0** | 0.0642 | -0.0735 | 0.0178 | **0** | 0.197 | 0.0714 |
| *C.nil* | 68.5 | 24 |  | **0** | 0.0589 |  |  | **0** | 0.195 | 0.0690 |
| *C.nil* | 71.3 | 25 | 0.140 | **0** | 0.0596 | -0.0748 | 0.0206 | **0** | 0.195 | 0.0599 |
| *C.nil* | 74.1 | 26 |  | **0** | 0.0529 | -0.0479 |  | **0** | 0.193 | 0.0571 |
| *C.nil* | 76.9 | 27 |  | **0** | 0.0467 | -0.0412 |  | **0** | 0.190 | 0.0536 |
| *C.nil* | 79.5 | 28 |  | **0** | 0.0569 | -0.0159 |  | **0** | 0.180 | 0.0420 |
| *C.nil* | 82.1 | 29 | 0.0856 | **0** | 0.0512 | -0.0156 | 0.0106 | **0** | 0.179 | 0.0386 |
| *C.nil* | 84.7 | 30 | 0.0850 | **0** | 0.0418 | **0** | 0.00707 | **0** | 0.178 | 0.0366 |
| *C.nil* | 87.3 | 31 | 0.0689 | **0** | 0.0368 | **0** | 0.0135 | **0** | 0.174 | 0.0330 |
| *C.nil* | 89.7 | 32 | 0.0512 | **0** | 0.0377 | **0** | 0.00803 | **0** | 0.167 | 0.0270 |
| *C.nil* | 91.9 | 33 |  | **0** | 0.0312 | **0** |  | **0** | 0.153 | 0.0222 |
| *C.nil* | 94.0 | 34 |  | **0** | 0.0263 | **0** |  | **0** | 0.144 | 0.0207 |
| *C.nil* | 95.8 | 35 | 0.0162 | **0** | 0.0237 | **0** | 0.00481 | **0** | 0.120 | 0.0178 |
| *C.nil* | 97.5 | 36 | **0** | **0** | 0.0127 | **0** | **0** | **0** | 0.116 | 0.0150 |
| *C.nil* | 98.8 | 37 | **0** | **0** | 0.0116 | **0** | **0** | **0** | 0.0950 | 0.0129 |
| *C.nil* | 100 | 38 | **0** | **0** | 0.00990 | **0** | **0** | **0** | 0.0797 | 0.00688 |
| *C.sia* | 2.25 | 1 | 0.234 | 0.048 | 0.116 | **0** | 0.065 | 0.026 | 0.149 | 0.517 |
| *C.sia* | 4.80 | 2 | 0.216 | 0.00954 | 0.117 | -0.164 | 0.063 | 0.042 | 0.170 | 0.507 |
| *C.sia* | 7.45 | 3 | 0.232 | 0.035 | 0.112 | -0.144 | 0.0587 | 0.032 | 0.177 | 0.501 |
| *C.sia* | 10.5 | 4 | 0.236 | 0.044 | 0.117 | -0.266 | 0.057 | 0.031 | 0.200 | 0.487 |
| *C.sia* | 13.6 | 5 | 0.229 | 0.023 | 0.103 | -0.188 | 0.044 | 0.042 | 0.205 | 0.463 |
| *C.sia* | 16.6 | 6 | 0.232 | 0.0439 | 0.114 | -0.207 | 0.050 | 0.034 | 0.200 | 0.451 |
| *C.sia* | 19.7 | 7 | 0.221 | 0.028 | 0.091 | -0.199 | 0.051 | 0.048 | 0.207 | 0.437 |
| *C.sia* | 22.8 | 8 | 0.216 | 0.036 | 0.091 | -0.191 | 0.045 | 0.062 | 0.207 | 0.409 |
| *C.sia* | 25.9 | 9 | 0.209 | 0.030 | 0.087 | -0.222 | 0.040 | 0.063 | 0.208 | 0.389 |
| *C.sia* | 29.0 | 10 | 0.222 | 0.03 | 0.079 | -0.166 | 0.039 | 0.052 | 0.206 | 0.375 |
| *C.sia* | 32.2 | 11 | 0.235 | 0.031 | 0.091 | -0.212 | 0.041 | 0.058 | 0.210 |  |
| *C.sia* | 35.5 | 12 |  | 0.03 | 0.0803 | -0.167 |  | 0.0362 | 0.220 | 0.329 |
| *C.sia* | 38.8 | 13 | 0.185 | 0.0313 | 0.0864 |  | 0.0283 | 0.0477 | 0.220 | 0.288 |
| *C.sia* | 42.1 | 14 | 0.245 | 0.0303 | 0.0802 |  | 0.0336 | 0.0218 | 0.219 | 0.164 |
| *C.sia* | 45.5 | 15 |  | **0** | 0.0698 | -0.118 |  | **0** | 0.224 | 0.106 |
| *C.sia* | 48.9 | 16 |  | **0** | 0.0766 |  |  | **0** | 0.226 | 0.109 |
| *C.sia* | 52.2 | 17 |  | **0** | 0.0751 |  |  | **0** | 0.223 | 0.107 |
| *C.sia* | 55.6 | 18 |  | **0** | 0.0675 | -0.131 |  | **0** | 0.223 | 0.105 |
| *C.sia* | 59.0 | 19 |  | **0** | 0.0771 | -0.109 |  | **0** | 0.227 | 0.0974 |
| *C.sia* | 62.3 | 20 |  | **0** | 0.0721 |  |  | **0** | 0.224 | 0.0934 |
| *C.sia* | 65.5 | 21 |  | **0** | 0.0616 | -0.113 |  | **0** | 0.214 | 0.0831 |
| *C.sia* | 68.8 | 22 | 0.247 | **0** | 0.0598 | -0.112 | 0.0268 | **0** | 0.217 | 0.0807 |
| *C.sia* | 72.0 | 23 | 0.207 | **0** | 0.0631 | -0.109 | 0.0255 | **0** | 0.214 | 0.0788 |
| *C.sia* | 75.2 | 24 | 0.194 | **0** | 0.0635 | -0.108 | 0.0272 | **0** | 0.212 | 0.0725 |
| *C.sia* | 78.3 | 25 | 0.182 | **0** | 0.0581 | -0.0770 | 0.0232 | **0** | 0.206 | 0.0661 |
| *C.sia* | 81.2 | 26 | 0.156 | **0** | 0.0386 |  | 0.0239 | **0** | 0.194 | 0.0562 |
| *C.sia* | 84.0 | 27 | 0.111 | **0** | 0.0477 | -0.0487 | 0.0172 | **0** | 0.188 | 0.0510 |
| *C.sia* | 86.7 | 28 | 0.110 | **0** | 0.0482 | **0** | 0.0152 | **0** | 0.175 | 0.0488 |
| *C.sia* | 89.1 | 29 | 0.075 | **0** | 0.0336 | **0** | 0.0164 | **0** | 0.164 | 0.0437 |
| *C.sia* | 91.5 | 30 | 0.0651 | **0** | 0.0397 | **0** | 0.0091 | **0** | 0.153 | 0.0369 |
| *C.sia* | 93.5 | 31 |  | **0** | 0.0334 | **0** |  | **0** |  | 0.0372 |
| *C.sia* | 95.2 | 32 |  | **0** | 0.0256 | **0** |  | **0** | 0.114 | 0.0293 |
| *C.sia* | 96.5 | 33 | **0** | **0** | 0.0241 | **0** | **0** | **0** | 0.0902 | 0.0240 |
| *C.sia* | 97.7 | 34 | **0** | **0** | 0.0215 | **0** | **0** | **0** | 0.0806 | 0.0212 |
| *C.sia* | 99.0 | 35 | **0** | **0** | 0.0210 | **0** | **0** | **0** | 0.0816 | 0.0227 |
| *C.sia* | 100 | 36 | **0** | **0** | 0.0088 | **0** | **0** | **0** | 0.0681 | 0.0091 |
| *V.nil* | 1.65 | 1 | 0.241 | 0.016 | 0.0601 | **0** | 0.0928 | 0.0507 | 0.137 | 0.492 |
| *V.nil* | 3.55 | 2 | 0.263 | 0.013 | 0.0572 | **0** | 0.0860 | 0.0475 | 0.159 | 0.497 |
| *V.nil* | 5.56 | 3 | 0.260 | 0.022 | 0.0562 | **0** | 0.0866 | 0.0410 | 0.167 | 0.478 |
| *V.nil* | 7.64 | 4 | 0.262 | 0.028 | 0.0661 | **0** | 0.0775 | 0.0378 | 0.173 | 0.452 |
| *V.nil* | 9.59 | 5 | 0.260 | 0.021 | 0.0582 | -0.146 | 0.0848 | 0.0442 | 0.162 | 0.431 |
| *V.nil* | 11.5 | 6 | 0.277 | 0.016 | 0.0575 | -0.139 | 0.0791 | 0.0316 | 0.160 | 0.403 |
| *V.nil* | 13.5 | 7 | 0.281 | 0.014 | 0.0564 | -0.166 | 0.0799 | 0.0396 | 0.166 | 0.382 |
| *V.nil* | 15.5 | 8 | 0.274 | 0.00953 | 0.0524 | -0.155 | 0.0747 | 0.0405 | 0.165 | 0.367 |
| *V.nil* | 17.5 | 9 | 0.279 | 0.0114 | 0.0596 | -0.188 | 0.0680 | 0.0372 | 0.164 | 0.356 |
| *V.nil* | 19.5 | 10 | 0.277 | 0.0126 | 0.0599 | -0.194 | 0.0620 | 0.0361 | 0.173 | 0.347 |
| *V.nil* | 21.6 | 11 | 0.293 | 0.014 | 0.0564 | -0.182 | 0.0666 | 0.0453 | 0.175 | 0.336 |
| *V.nil* | 23.5 | 12 | 0.271 | 0.0131 | 0.0701 | -0.195 | 0.0621 | 0.0354 | 0.154 | 0.324 |
| *V.nil* | 25.2 | 13 | 0.272 | 0.0127 | 0.0661 | -0.193 | 0.0596 | 0.0361 | 0.139 | 0.320 |
| *V.nil* | 26.9 | 14 |  | 0.017 | 0.0647 | -0.192 |  | 0.0243 | 0.144 | 0.308 |
| *V.nil* | 28.8 | 15 | 0.271 | 0.000238 | 0.0570 | -0.180 | 0.0570 | 0.0281 | 0.157 | 0.304 |
| *V.nil* | 30.5 | 16 | 0.247 | 0.00485 | 0.0691 | -0.188 | 0.0435 | 0.0372 | 0.142 | 0.291 |
| *V.nil* | 32.3 | 17 | 0.249 | 0.00659 | 0.0634 | -0.177 | 0.0531 | 0.0326 | 0.152 | 0.287 |
| *V.nil* | 34.0 | 18 | 0.254 | -0.00437 | 0.0462 | -0.173 | 0.0524 | 0.0265 | 0.144 | 0.274 |
| *V.nil* | 35.9 | 19 | 0.245 | -0.00914 | 0.0529 | -0.173 | 0.0531 | 0.0324 | 0.156 | 0.281 |
| *V.nil* | 37.7 | 20 | 0.249 | 0.018 | 0.0529 | -0.162 | 0.0531 | 0.0305 | 0.148 | 0.267 |
| *V.nil* | 39.4 | 21 | 0.253 | 0.0189 | 0.0426 | -0.152 | 0.0529 | 0.0273 | 0.145 | 0.256 |
| *V.nil* | 41.2 | 22 | 0.233 | -0.00326 | 0.0516 | -0.163 | 0.0496 | 0.0280 | 0.146 | 0.248 |
| *V.nil* | 42.9 | 23 | 0.233 | 0.00413 | 0.0534 | -0.155 | 0.0505 | 0.0291 | 0.145 |  |
| *V.nil* | 44.6 | 24 | 0.220 | 0.00667 | 0.0480 | -0.168 | 0.0523 | 0.0419 | 0.142 |  |
| *V.nil* | 46.3 | 25 | 0.226 | -0.00103 | 0.0510 | -0.153 | 0.0548 | 0.0442 | 0.135 | 0.226 |
| *V.nil* | 47.8 | 26 | 0.213 | 0.00429 | 0.0480 | -0.150 | 0.0497 | 0.0338 | 0.128 | 0.217 |
| *V.nil* | 49.3 | 27 | 0.218 | 0.0157 | 0.0446 | -0.137 | 0.0499 | 0.0384 | 0.130 | 0.203 |
| *V.nil* | 50.9 | 28 | 0.178 | -0.00786 | 0.0421 | -0.134 | 0.0480 | 0.0423 | 0.131 | 0.204 |
| *V.nil* | 52.5 | 29 | 0.199 | 0.00246 | 0.0418 | -0.144 | 0.0446 | 0.0316 | 0.130 | 0.188 |
| *V.nil* | 54.0 | 30 | 0.201 | -0.00747 | 0.0407 | -0.142 | 0.0510 | 0.0319 | 0.124 | 0.198 |
| *V.nil* | 55.6 | 31 | 0.191 | 0.00429 | 0.0451 | -0.158 | 0.0505 | 0.0332 | 0.132 | 0.194 |
| *V.nil* | 57.2 | 32 | 0.181 | -0.00167 | 0.0440 | -0.120 | 0.0508 | 0.0388 | 0.140 | 0.187 |
| *V.nil* | 58.9 | 33 | 0.174 | -0.00961 | 0.0462 | -0.134 | 0.0467 | 0.0305 | 0.137 |  |
| *V.nil* | 60.5 | 34 | 0.167 | -0.00302 | 0.0413 | -0.136 | 0.0418 | 0.0370 | 0.137 | 0.176 |
| *V.nil* | 62.1 | 35 | 0.171 | -0.0085 | 0.0462 | -0.121 | 0.0377 | 0.0377 | 0.132 | 0.176 |
| *V.nil* | 63.7 | 36 | 0.161 | -0.00922 | 0.0426 | -0.114 | 0.0337 | 0.0337 | 0.133 | 0.171 |
| *V.nil* | 65.2 | 37 | 0.162 | -0.0156 | 0.0410 | -0.127 | 0.0389 | 0.0319 | 0.125 | 0.167 |
| *V.nil* | 66.8 | 38 | 0.140 | -0.00238 | 0.0397 | -0.115 | 0.0378 | 0.0307 | 0.130 | 0.162 |
| *V.nil* | 68.3 | 39 | 0.141 | 0.00087 | 0.0402 | -0.119 | 0.0324 | 0.0315 | 0.128 | 0.157 |
| *V.nil* | 69.9 | 40 | 0.144 | 0.00056 | 0.0345 | -0.107 | 0.0305 | 0.0281 | 0.128 | 0.154 |
| *V.nil* | 71.3 | 41 | 0.133 | 0.00159 | 0.0384 | -0.108 | 0.0313 | 0.0356 | 0.123 |  |
| *V.nil* | 72.9 | 42 | 0.132 | 0.00651 | 0.0410 | -0.0941 | 0.0297 | 0.0329 | 0.127 |  |
| *V.nil* | 74.4 | 43 | 0.128 | 0.00763 | 0.0318 | -0.101 | 0.0318 | 0.0319 | 0.125 | 0.147 |
| *V.nil* | 75.9 | 44 | 0.121 | 0.00620 | 0.0381 | -0.0963 | 0.0272 | 0.0272 | 0.129 | 0.142 |
| *V.nil* | 77.3 | 45 | 0.122 | 0.00453 | 0.0389 | -0.0882 | 0.0272 | 0.0267 | 0.120 | 0.134 |
| *V.nil* | 78.8 | 46 | 0.117 | 0.00389 | 0.0307 | -0.0872 | 0.0205 | 0.0353 | 0.120 | 0.131 |
| *V.nil* | 80.2 | 47 | 0.110 | 0.0076 | 0.0353 | -0.0812 | 0.0194 | 0.0294 | 0.118 | 0.126 |
| *V.nil* | 81.7 | 48 | 0.111 | 0.00429 | 0.0353 | -0.0826 | 0.0241 | 0.0241 | 0.123 | 0.123 |
| *V.nil* | 83.0 | 49 | 0.102 | 0.00850 | 0.0323 | -0.0860 | 0.0195 | 0.0261 | 0.114 | 0.123 |
| *V.nil* | 84.4 | 50 | 0.100 | 0.0178 | 0.0334 | -0.0736 | 0.0192 | 0.0302 | 0.111 | 0.116 |
| *V.nil* | 85.7 | 51 | 0.0961 | 0.0160 | 0.0350 | -0.0724 | 0.0159 | 0.0288 | 0.111 | 0.115 |
| *V.nil* | 87.1 | 52 |  | 0.0119 | 0.0324 | -0.0666 |  | 0.0257 | 0.114 | 0.111 |
| *V.nil* | 88.6 | 53 | 0.0910 | 0.0133 | 0.0302 | -0.0680 | 0.014 | 0.0257 | 0.123 | 0.110 |
| *V.nil* | 89.9 | 54 | 0.0891 | 0.00421 | 0.0297 | -0.0582 | 0.013 | 0.0195 | 0.109 | 0.104 |
| *V.nil* | 91.2 | 55 | 0.0790 | 0.0160 | 0.0288 | -0.0618 | 0.011 | 0.0264 | 0.108 |  |
| *V.nil* | 92.5 | 56 |  | 0.0105 | 0.0343 | -0.0696 |  | 0.0256 | 0.112 | 0.100 |
| *V.nil* | 93.9 | 57 |  | 0.00763 | 0.0299 | -0.0550 |  | 0.0291 | 0.110 | 0.0968 |
| *V.nil* | 95.2 | 58 | 0.0721 | 0.00540 | 0.0286 | -0.0542 | 0.013 | 0.0237 | 0.109 | 0.0988 |
| *V.nil* | 96.4 | 59 |  |  | 0.0286 | -0.0589 |  | 0.0216 | 0.104 |  |
| *V.nil* | 97.6 | 60 |  |  |  |  | 0.0097 | 0.0286 |  | 0.0933 |
| *V.nil* | 98.8 | 61 |  |  |  |  |  |  |  | 0.0925 |
| *V.nil* | 100 | 62 |  |  |  |  |  |  |  |  |
| *V.ko* | 2.38 | 1 | 0.238 | 0.00888 | 0.0901 | **0** | 0.118 | 0.0356 | 0.167 | 0.574 |
| *V.ko* | 4.70 | 2 | 0.258 | 0.00941 | 0.0699 | -0.136 | 0.105 | 0.0509 | 0.163 | 0.590 |
| *V.ko* | 6.97 | 3 | 0.261 | 0.0176 | 0.0757 | -0.174 | 0.104 | 0.0409 | 0.159 | 0.564 |
| *V.ko* | 9.61 | 4 | 0.260 | 0.00769 | 0.0731 | -0.202 | 0.0970 | 0.0353 | 0.186 | 0.541 |
| *V.ko* | 12.1 | 5 | 0.256 | 0.00133 | 0.0707 | -0.201 | 0.101 | 0.0296 | 0.171 | 0.518 |
| *V.ko* | 14.3 | 6 | 0.251 | -0.0138 | 0.0607 | -0.209 | 0.0887 | 0.0305 | 0.157 | 0.484 |
| *V.ko* | 16.3 | 7 |  | -0.0100 | 0.0576 | -0.218 |  | 0.0324 | 0.142 | 0.433 |
| *V.ko* | 18.4 | 8 | 0.255 | -0.00494 | 0.0533 | -0.180 | 0.0798 | 0.0310 | 0.145 |  |
| *V.ko* | 20.5 | 9 | 0.280 | 0.000651 | 0.0588 | -0.161 | 0.0729 | 0.0438 | 0.150 | 0.430 |
| *V.ko* | 22.7 | 10 | 0.281 | -0.0123 | 0.0501 | -0.187 | 0.0766 | 0.0321 | 0.153 | 0.412 |
| *V.ko* | 24.8 | 11 | 0.264 | -0.0130 | 0.0516 | -0.143 | 0.0814 | 0.0294 | 0.148 |  |
| *V.ko* | 26.8 | 12 | 0.266 | -0.0235 | 0.0491 | -0.126 | 0.0805 | 0.0271 | 0.140 | 0.382 |
| *V.ko* | 29.1 | 13 | 0.250 | -0.024 | 0.0509 |  | 0.0827 | 0.0276 | 0.164 |  |
| *V.ko* | 31.2 | 14 | 0.255 | -0.015 | 0.0422 | -0.123 | 0.0731 | 0.0308 | 0.145 |  |
| *V.ko* | 33.2 | 15 | 0.234 | -0.0430 | 0.0467 |  | 0.0719 | 0.0347 | 0.140 |  |
| *V.ko* | 35.2 | 16 | 0.225 | -0.0469 | 0.0394 |  | 0.0737 | 0.0367 | 0.142 |  |
| *V.ko* | 37.0 | 17 | 0.218 | -0.0567 | 0.0368 |  | 0.0763 | 0.0372 | 0.128 |  |
| *V.ko* | 38.9 | 18 | 0.196 | -0.0574 | 0.0466 |  | 0.0662 | 0.0433 | 0.135 | 0.307 |
| *V.ko* | 41.0 | 19 | 0.206 | -0.0520 | 0.0392 | -0.128 | 0.0608 | 0.0359 | 0.146 | 0.295 |
| *V.ko* | 42.8 | 20 | 0.186 | -0.0473 | 0.0331 | -0.131 | 0.0604 | 0.0370 | 0.126 | 0.284 |
| *V.ko* | 44.7 | 21 | 0.184 | -0.0479 | 0.0405 |  | 0.0647 | 0.0440 | 0.136 | 0.277 |
| *V.ko* | 46.6 | 22 | 0.174 | -0.0503 | 0.0383 | -0.121 | 0.0596 | 0.0370 | 0.127 | 0.264 |
| *V.ko* | 48.4 | 23 | 0.174 | -0.0488 | 0.0402 | -0.122 | 0.0564 | 0.0369 | 0.128 | 0.259 |
| *V.ko* | 50.3 | 24 | 0.163 | -0.0497 | 0.0409 | -0.112 | 0.0559 | 0.0308 | 0.132 | 0.244 |
| *V.ko* | 52.0 | 25 |  | -0.0278 |  | -0.116 | 0.0565 | 0.0415 |  | 0.244 |
| *V.ko* | 53.8 | 26 | 0.153 | -0.0509 | 0.0340 | -0.125 | 0.0500 | 0.0389 | 0.126 | 0.237 |
| *V.ko* | 55.4 | 27 |  | -0.0291 |  | -0.137 | 0.0489 | 0.0356 | 0.118 |  |
| *V.ko* | 57.1 | 28 | 0.139 | -0.0414 | 0.0383 | -0.102 | 0.0513 | 0.0328 | 0.113 | 0.214 |
| *V.ko* | 58.7 | 29 |  | -0.0224 |  | -0.125 | 0.0404 | 0.0377 | 0.114 | 0.208 |
| *V.ko* | 60.4 | 30 | 0.111 | -0.0399 | 0.0286 |  | 0.0414 | 0.0427 | 0.119 | 0.199 |
| *V.ko* | 61.9 | 31 | 0.120 | -0.0389 | 0.0295 |  | 0.0441 | 0.0381 | 0.106 | 0.185 |
| *V.ko* | 63.6 | 32 | 0.109 | -0.0395 | 0.0269 | -0.0858 | 0.0391 | 0.0392 | 0.120 | 0.184 |
| *V.ko* | 65.1 | 33 |  | -0.0298 |  | -0.0930 | 0.0364 | 0.0330 | 0.105 | 0.165 |
| *V.ko* | 66.5 | 34 | 0.101 | -0.0314 | 0.0229 | -0.102 | 0.0211 | 0.0293 | 0.0992 | 0.159 |
| *V.ko* | 67.9 | 35 | 0.109 | -0.0356 | 0.0316 | -0.0717 | 0.0369 | 0.0343 | 0.102 | 0.167 |
| *V.ko* | 69.4 | 36 | 0.102 | -0.0292 | 0.0225 |  | 0.0314 | 0.0332 | 0.100 | 0.152 |
| *V.ko* | 70.7 | 37 | 0.0927 | -0.0327 | 0.0280 |  | 0.0324 | 0.0331 | 0.0913 | 0.153 |
| *V.ko* | 72.0 | 38 | 0.0903 | -0.0298 | 0.0244 |  | 0.0273 | 0.0322 | 0.0936 | 0.145 |
| *V.ko* | 73.2 | 39 | 0.0885 | -0.0362 | 0.0236 |  | 0.0319 | 0.0348 | 0.0873 | 0.144 |
| *V.ko* | 74.5 | 40 | 0.0776 | -0.0244 | 0.0208 | -0.0441 | 0.0309 | 0.0343 | 0.0895 | 0.131 |
| *V.ko* | 75.8 | 41 | 0.0744 | -0.0220 | 0.0252 |  | 0.0259 | 0.0385 | 0.0892 |  |
| *V.ko* | 77.1 | 42 |  | -0.0172 | 0.0236 | -0.0542 |  | 0.0318 | 0.0907 | 0.123 |
| *V.ko* | 78.2 | 43 | 0.0757 | -0.031 | 0.0202 |  | 0.0256 | 0.0293 | 0.0796 | 0.125 |
| *V.ko* | 79.3 | 44 | 0.0717 | -0.0195 | 0.0202 | -0.0443 | 0.0263 | 0.0307 | 0.0787 | 0.120 |
| *V.ko* | 80.5 | 45 | 0.0760 | -0.0201 | 0.0180 |  | 0.0279 | 0.0282 | 0.0822 | 0.110 |
| *V.ko* | 81.6 | 46 | 0.0637 | -0.0245 | 0.0223 | -0.0357 | 0.0260 | 0.0228 | 0.0793 | 0.107 |
| *V.ko* | 82.7 | 47 | 0.055 | -0.0227 | 0.0166 |  |  | 0.0285 | 0.0736 | 0.107 |
| *V.ko* | 83.7 | 48 | 0.0538 | -0.0131 | 0.0178 | -0.0349 | 0.0263 | 0.0294 | 0.0730 | 0.101 |
| *V.ko* | 84.7 | 49 | 0.0537 | -0.011 | 0.0220 | -0.0324 | 0.0288 | 0.0264 | 0.0654 | 0.0931 |
| *V.ko* | 85.7 | 50 | 0.0491 | -0.00704 | 0.0219 | -0.0285 | 0.0144 | 0.0272 | 0.0726 | 0.0908 |
| *V.ko* | 86.6 | 51 | 0.0480 | -0.00864 | 0.0195 | -0.0301 | 0.0136 | 0.0230 | 0.0662 | 0.0910 |
| *V.ko* | 87.6 | 52 | 0.0477 | -0.00476 | 0.0179 | -0.0194 | 0.0221 | 0.0222 | 0.0681 | 0.0862 |
| *V.ko* | 88.4 | 53 | 0.0467 | -0.00101 | 0.0148 | -0.0202 | 0.0175 | 0.0251 | 0.0579 |  |
| *V.ko* | 89.3 | 54 | 0.0390 | -0.000296 | 0.0148 | -0.0252 | 0.0112 | 0.0193 | 0.0623 |  |
| *V.ko* | 90.1 | 55 | 0.0454 | -0.000976 | 0.0134 | -0.0187 | 0.0141 | 0.0232 | 0.0565 |  |
| *V.ko* | 90.9 | 56 | 0.0381 | -0.00840 | 0.0124 |  | 0.0130 | 0.0212 | 0.0566 |  |
| *V.ko* | 91.7 | 57 | 0.0385 | -0.00222 | 0.0132 | -0.0199 | 0.0113 | 0.0156 | 0.0520 |  |
| *V.ko* | 92.5 | 58 | 0.0308 | -0.00189 | 0.0105 |  | 0.00858 | 0.0169 | 0.0573 | 0.0698 |
| *V.ko* | 93.1 | 59 | 0.0309 | -0.00967 | 0.0154 | -0.0112 | 0.00888 | 0.0168 | 0.0432 | 0.0633 |
| *V.ko* | 93.8 | 60 | 0.0321 | -0.0130 | 0.0133 | -0.0120 | 0.0101 | 0.0157 | 0.0525 | 0.0613 |
| *V.ko* | 94.6 | 61 | 0.0354 | -0.00692 | 0.0118 |  | 0.00941 | 0.0202 | 0.0505 |  |
| *V.ko* | 95.2 | 62 | 0.0288 | -0.00195 | 0.0129 | -0.0182 | 0.00598 | 0.0167 | 0.0474 | 0.0525 |
| *V.ko* | 95.9 | 63 | 0.0270 | -0.00228 | 0.0109 | -0.0130 | 0.00751 | 0.0163 | 0.0443 | 0.0490 |
| *V.ko* | 96.4 | 64 | 0.0236 | -0.00494 | 0.0125 |  | 0.00627 | 0.0158 | 0.0408 |  |
| *V.ko* | 97.1 | 65 | 0.0178 |  | 0.0131 |  | 0.0023 | 0.0172 | 0.0424 | 0.0491 |
| *V.ko* | 97.7 | 66 | 0.0267 | -0.00577 | 0.0111 |  | 0.0051 | 0.0124 | 0.0438 | 0.0489 |
| *V.ko* | 98.3 | 67 | 0.0212 | -0.00577 | 0.00976 |  | 0.0035 | 0.0148 |  | 0.0473 |
| *V.ko* | 98.9 | 68 | 0.0240 | -0.00769 | 0.00935 |  | 0.0040 | 0.0120 | 0.0422 | 0.0471 |
| *V.ko* | 99.4 | 69 | 0.0245 | -0.00482 | 0.00704 |  | 0.0053 | 0.0114 | 0.0393 | 0.0455 |
| *V.ko* | 100 | 70 |  | -0.00683 | 0.0107 |  |  | 0.00959 | 0.0407 |  |
| *Acin* | 3.52 | 1 | 0.0649 | 0.0179 | 0.0201 | **0** | 0.0281 | 0.0220 | 0.0878 | 0.156 |
| *Acin* | 6.91 | 2 | **0** | 0.00072 | 0.0214 | **0** | **0** | 0.0257 | 0.0847 | 0.143 |
| *Acin* | 10.7 | 3 | **0** | 0.00669 | 0.0220 | **0** | **0** | 0.0465 | 0.0955 | 0.123 |
| *Acin* | 15.1 | 4 | **0** | 0.0126 | 0.0215 | **0** | **0** | 0.0248 | 0.108 | 0.136 |
| *Acin* | 19.9 | 5 | **0** | 0.00876 | 0.0223 | **0** | **0** | 0.0295 | 0.120 | 0.117 |
| *Acin* | 25.1 | 6 | **0** | 0.00556 | 0.0232 | **0** | **0** | 0.0314 | 0.130 | 0.0992 |
| *Acin* | 30.8 | 7 | **0** | 0.0031 | 0.0229 | **0** | **0** | 0.0228 | 0.143 | 0.0833 |
| *Acin* | 36.7 | 8 | **0** | **0** | 0.0212 | **0** | **0** | **0** | 0.148 | 0.0729 |
| *Acin* | 42.9 | 9 | **0** | **0** | 0.0220 | **0** | **0** | **0** | 0.154 | 0.0653 |
| *Acin* | 49.3 | 10 | **0** | **0** | 0.0207 | **0** | **0** | **0** | 0.160 | 0.0554 |
| *Acin* | 55.4 | 11 | **0** | **0** | 0.0212 | **0** | **0** | **0** | 0.152 | 0.0500 |
| *Acin* | 61.2 | 12 | **0** | **0** | 0.0189 | **0** | **0** | **0** | 0.147 | 0.0426 |
| *Acin* | 66.8 | 13 | **0** | **0** | 0.0194 | **0** | **0** | **0** | 0.139 | 0.0388 |
| *Acin* | 72.1 | 14 | **0** | **0** | 0.0174 | **0** | **0** | **0** | 0.133 | 0.0307 |
| *Acin* | 76.9 | 15 | **0** | **0** | 0.0162 | **0** | **0** | **0** | 0.118 | 0.0279 |
| *Acin* | 81.0 | 16 | **0** | **0** | 0.0147 | **0** | **0** | **0** | 0.103 | 0.0234 |
| *Acin* | 84.6 | 17 | **0** | **0** | 0.0126 | **0** | **0** | **0** | 0.0920 | 0.0221 |
| *Acin* | 88.0 | 18 | **0** | **0** | 0.0098 | **0** | **0** | **0** | 0.0827 | 0.0167 |
| *Acin* | 91.7 | 19 | **0** | **0** | 0.0083 | **0** | **0** | **0** | 0.0935 | 0.0130 |
| *Acin* | 95.0 | 20 | **0** | **0** | 0.0077 | **0** | **0** | **0** | 0.0812 | 0.0109 |
| *Acin* | 97.6 | 21 | **0** | **0** | 0.0062 | **0** | **0** | **0** | 0.0666 | 0.0111 |
| *Acin* | 99.3 | 22 | **0** | **0** | 0.0057 | **0** | **0** | **0** | 0.0411 | 0.0114 |
| *Acin* | 100 | 23 | **0** | **0** | 0.0041 | **0** | **0** | **0** | 0.0185 | 0.00689 |
| *Macr* | 4.01 | 1 | **0** | 0.0406 | 0.109 | **0** | **0** | 0.171 | 0.168 | 0.269 |
| *Macr* | 8.44 | 2 | **0** | 0.0471 | 0.113 | **0** | **0** | 0.201 | 0.186 | 0.300 |
| *Macr* | 12.8 | 3 | **0** | 0.0399 | 0.113 | -0.0978 | **0** | 0.124 | 0.181 | 0.299 |
| *Macr* | 17.3 | 4 | **0** | 0.0107 | 0.106 | -0.242 | **0** | 0.117 | 0.191 | 0.299 |
| *Macr* | 22.9 | 5 | **0** | 0.00981 | 0.113 | -0.204 | **0** | 0.103 | 0.234 | 0.288 |
| *Macr* | 29.4 | 6 | **0** | 0.0241 | 0.110 | -0.238 | **0** | 0.102 | 0.272 | 0.268 |
| *Macr* | 36.4 | 7 | **0** | 0.0225 | 0.116 | -0.162 | **0** | 0.0744 | 0.296 | 0.235 |
| *Macr* | 43.6 | 8 | **0** | -0.000194 | 0.119 | -0.0672 | **0** | 0.0564 | 0.303 | 0.200 |
| *Macr* | 50.1 | 9 | **0** | -0.00643 | 0.120 | -0.190 | **0** | 0.0589 | 0.270 | 0.167 |
| *Macr* | 56.9 | 10 | **0** | -0.0203 | 0.100 | -0.196 | **0** | 0.0554 | 0.285 | 0.171 |
| *Macr* | 63.4 | 11 | **0** | -0.0165 | 0.111 | -0.187 | **0** | 0.0581 | 0.273 | 0.168 |
| *Macr* | 69.5 | 12 | **0** | -0.0205 | 0.101 | -0.186 | **0** | 0.0565 | 0.256 | 0.160 |
| *Macr* | 75.1 | 13 | **0** | -0.0135 | 0.0911 | -0.180 | **0** | 0.0531 | 0.236 | 0.144 |
| *Macr* | 80.2 | 14 | **0** | -0.00750 | 0.0856 | -0.0710 | **0** | 0.0479 | 0.215 | 0.129 |
| *Macr* | 84.6 | 15 | **0** | -0.0116 | 0.0797 | -0.0526 | **0** | 0.0406 | 0.183 | 0.114 |
| *Macr* | 88.5 | 16 | **0** | -0.00169 | 0.0686 | -0.108 | **0** | 0.0352 | 0.167 | 0.0981 |
| *Macr* | 92.0 | 17 | **0** | -0.00349 | 0.0540 | -0.0803 | **0** | 0.0299 | 0.145 | 0.0782 |
| *Macr* | 94.9 | 18 | **0** | 0.00535 | 0.0420 | **0** | **0** | 0.0243 | 0.123 | 0.0623 |
| *Macr* | 96.8 | 19 | **0** | 0.00182 | 0.0341 | **0** | **0** | 0.0299 | 0.0773 | 0.0562 |
| *Macr* | 98.5 | 20 | **0** | 0.00380 | 0.0284 | **0** | **0** | 0.0112 | 0.0733 | 0.0323 |
| *Macr* | 99.5 | 21 | **0** | **0** | 0.0218 | **0** | **0** | **0** | 0.0408 | 0.0221 |
| *Macr* | 100 | 22 | **0** | **0** | 0.0209 | **0** | **0** | **0** | 0.0222 | 0.0169 |

Table S3. Complete dataset (all taxa studied and all vertebral parameters). Taxon abbreviations are given in Table S1. The loss of a vertebral feature is indicated by the zero values in bold.

Table S4. Averages of the tail values by tail region (proximal, middle and distal)

| Vertebrae | Height normalised with femur length | | | Depth normalised with femur length | Length normalised with femur length | | | Width normalised with femur length |
| --- | --- | --- | --- | --- | --- | --- | --- | --- |
| Taxa | Neural spine | Transverse process | Centrum | Chevron | Neural spine | Transverse process | Centrum | Vertebral |
| *Coel*_prox | 0.0613 | 0.116 | 0.0911 |  | 0.0311 | 0.0649 | 0.165 | 0.0832 |
| *Coel*_mid | 0.00987 | 0.00891 | 0.0600 |  | 0.0148 | 0.0185 | 0.195 |  |
| *Coel*_distal | 0 | 0 | 0.0332 |  | 0 | 0 | 0.182 |  |
| *Dilo*_prox | 0.107 | 0.0826 | 0.0735 | -0.165 | 0.0257 | 0.0467 | 0.138 |  |
| *Dilo*_mid | 0.0316 | 0.00341 | 0.0506 |  | 0.00590 | 0.00218 | 0.141 | 0.0485 |
| *Dilo*_distal | 0 | 0 | 0.0265 |  | 0 | 0 | 0.130 | 0.0297 |
| *Trex*_prox | 0.176 | 0.113 | 0.136 | -0.132 | 0.0810 | 0.0641 | 0.137 | 0.231 |
| *Trex*_mid | 0.0757 | 0.0136 | 0.0898 | -0.0598 | 0.029 | 0.00976 | 0.133 | 0.111 |
| *Trex*_distal | 0.0453 | 0 | 0.0637 | -0.0490 | 0.0224 | 0 | 0.108 | 0.0802 |
| *Jura*_prox | 0.0466 | 0.0770 | 0.0815 | -0.125 | 0.0128 | 0.0530 | 0.131 |  |
| *Jura*_mid | 0 |  | 0.0641 | -0.0664 | 0 |  | 0.149 |  |
| *Jura*_distal | 0 |  | 0.0591 |  | 0 |  | 0.150 |  |
| *Comp*_prox | 0.120 |  | 0.0607 | -0.154 | 0.0743 |  | 0.150 |  |
| *Ornit*_prox |  | 0.0637 | 0.0911 |  |  | 0.0151 | 0.148 |  |
| *Ornit*_mid | 0.0317 | 0 | 0.0489 | -0.0210 | 0.135 | 0 | 0.180 | 0.0644 |
| *Ornit*_distal |  | 0 | 0.0162 |  |  | 0 | 0.163 | 0.0386 |
| *Shuv*_prox | 0.0680 | 0.0531 | 0.0724 | -0.0539 | 0.0360 | 0.0419 | 0.166 |  |
| *Shuv*_mid | 0.0530 | 0.0292 | 0.0530 |  | 0.0328 | 0.0292 | 0.112 | 0.0659 |
| *Citi*_prox |  |  |  | -0.0878 |  | 0.0361 |  | 0.298 |
| *Citi*_mid | 0.0584 | 0.00118 | 0.0423 | -0.0863 | 0.0306 | 0.0612 | 0.0860 | 0.180 |
| *Citi*_distal | 0.0168 | 0.0011 | 0.0249 | -0.0188 | 0.0361 | 0.0393 | 0.0660 | 0.0663 |
| *Khaa*_prox | 0.125 | 0.0223 | 0.0737 |  | 0.0268 | 0.0353 | 0.0820 | 0.299 |
| *Khaa*_mid | 0.0244 | -0.00907 | 0.0187 |  | 0.0234 | 0.0451 |  | 0.244 |
| *Khaa*_distal | 0.0212 | -0.00937 | 0.0206 |  | 0.018 | 0.0280 | 0.0590 | 0.0615 |
| *Caud*_prox | 0.0612 |  | 0.0588 | -0.169 | 0.0279 |  | 0.0797 |  |
| *Caud*_mid | 0.00599 |  | 0.0658 | -0.0777 | 0.0116 | 0.0224 | 0.0729 |  |
| *Caud* _distal | 0 | 0 | 0.024 | -0.0120 | 0 | 0 | 0.0482 | 0.0297 |
| *Mei*_prox | 0.0214 | 0.019 | 0.0374 | -0.0394 | 0.0393 | 0.0137 | 0.101 | 0.0652 |
| *Mei*_mid | 0 | 0 | 0.0351 | -0.0281 | 0 | 0 | 0.171 | 0.0451 |
| *Mei*_distal | 0 | 0 | 0.024 | -0.0247 | 0 | 0 | 0.170 | 0.0276 |
| *Sino*_prox | 0 | 0.0135 | 0.0355 | -0.0319 | 0 | 0.0263 | 0.128 | 0.106 |
| *Sino*_mid | 0 | 0 | 0.0378 | -0.0132 | 0 | 0 | 0.189 | 0.0424 |
| *Sino*_distal | 0 | 0 | 0.0248 | -0.0128 | 0 | 0 | 0.161 | 0.0191 |
| *Sinu*_prox | 0 | 0.0321 | 0.0514 | -0.0856 | 0 | 0.0198 | 0.115 | 0.112 |
| *Sinu*_mid | 0 | 0 | 0.0436 | -0.0235 | 0 | 0 | 0.191 | 0.0446 |
| *Sinu*_distal | 0 | 0 | 0.0327 | -0.0186 | 0 | 0 | 0.163 | 0.0217 |
| *Maha*_prox | 0.0433 | 0.030 | 0.0479 | -0.0665 | 0.0422 | 0.0308 | 0.147 | 0.0643 |
| *Maha* _mid | 0.0215 | 0.0032 | 0.0429 | -0.0307 | 0.102 | 0.0091 | 0.155 | 0.0369 |
| *Dein*_prox | 0.0430 | 0.0258 | 0.0676 | -0.0578 | 0.0502 | 0.0512 | 0.142 | 0.0617 |
| *Dein*_mid | 0.0311 | 0 | 0.0452 | -0.0290 | 0.0870 | 0 | 0.146 | 0.0463 |
| *Dein*_distal | 0 | 0 | 0.020 | -0.0324 | 0 | 0 | 0.102 | 0.0260 |
| *Velo*_prox | 0.0820 | 0.013 | 0.0734 | -0.0740 | 0.0306 | 0.026 | 0.122 | 0.248 |
| *Velo*_mid | 0.00215 | 0 | 0.0473 | -0.0466 | 0.00327 | 0 | 0.116 | 0.0471 |
| *Vel*_F_prox | 0.173 |  | 0.0726 | -0.188 |  | 0.0586 | 0.151 |  |
| *Vel*­_F_mid |  | 0 |  | -0.0601 |  | 0 | 0.174 |  |
| *Vel*_F_distal |  | 0 |  | -0.0303 |  | 0 | 0.135 |  |
| *Bamb*_prox | 0.114 | 0.0086 | 0.0506 | -0.0383 | 0.0340 | 0.0497 | 0.151 | 0.0574 |
| *Bamb*_mid |  | 0 | 0.0466 |  |  | 0 |  |  |
| *Drom*_prox |  |  |  | -0.0774 |  | 0.0638 |  |  |
| *Drom*_mid | 0 |  | 0.0893 |  | 0 |  | 0.537 |  |
| *Drom*_distal | 0 |  | 0.0502 |  | 0 |  | 0.398 |  |
| *M*.*gui*_prox |  |  | 0.0650 |  |  |  | 0.318 |  |
| *M*.*gui*_mid |  |  | 0.0608 |  |  |  | 0.302 |  |
| *M.gui*_distal |  |  |  |  |  |  | 0.289 |  |
| *M.zha*_prox |  | 0 |  |  |  | 0 | 0.180 |  |
| *M.zha*_mid |  | 0 |  |  |  | 0 | 0.225 |  |
| *M.zha*_distal |  | 0 |  |  |  | 0 | 0.151 |  |
| *Epid*_prox |  |  |  |  |  | 0.0382 | 0.0758 | 0.250 |
| *Epid*_mid |  |  |  |  |  |  | 0.107 |  |
| *Epid*_distal |  |  |  |  |  |  | 0.0827 |  |
| *Ar*.B_prox | 0.0299 | 0.0058 | 0.0454 | -0.052 | 0.0183 | 0.00916 | 0.122 |  |
| *Ar*.B_mid |  | 0 | 0.0294 | -0.0223 |  | 0 | 0.215 |  |
| *Ar*.B_distal |  | 0 | 0.0351 |  |  | 0 | 0.198 |  |
| *Ar*.J_prox | 0 | 0.0072 | 0.043 | -0.0612 | 0 | 0.0344 | 0.134 |  |
| *Ar*.J_mid | 0 | 0 | 0.0435 | -0.032 | 0 | 0 | 0.226 |  |
| *Ar*.J_distal | 0 | 0 | 0.0369 | -0.025 | 0 | 0 | 0.171 | 0.019 |
| *Ar*.H_prox | 0 | 0.0043 | 0.0437 | -0.0717 | 0 | 0.029 | 0.126 |  |
| *Ar*.H_mid | 0 | 0 | 0.0407 | -0.016 | 0 | 0 | 0.208 |  |
| *Ar*.H_distal | 0 | 0 |  |  | 0 | 0 | 0.162 |  |
| *Jeho*_prox | 0.0319 | 0.0001 | 0.0631 | -0.0336 | 0.0103 | 0.0152 | 0.243 | 0.0605 |
| *Jeho*_mid | 0 | 0 | 0.0468 | -0.0192 | 0 | 0 | 0.258 |  |
| *Jeho*_distal | 0 | 0 | 0.026 | -0.013 | 0 | 0 | 0.193 | 0.012 |
| *Sape*_prox |  |  |  |  | 0.013 | 0.016 | 0.0627 | 0.136 |
| *Sape*_mid |  |  |  | -0.0416 |  | 0.0192 | 0.0653 | 0.124 |
| Sape_distal |  |  |  |  |  | 0.0284 | 0.0366 | 0.142 |
| *Conf*_prox |  |  |  |  |  | 0.016 | 0.0683 |  |
| *Hong*_prox | 0.019 |  | 0.0582 | -0.033 | 0.014 |  | 0.0499 |  |
| *Hong*_mid | 0.028 |  | 0.047 |  | 0.0083 |  | 0.043 |  |
| *Yixi*_prox | 0.0723 | 0.025 | 0.0678 |  | 0.022 | 0.023 | 0.0699 |  |
| *Yixi*_mid | 0.0447 |  | 0.0554 | -0.0420 | 0.022 |  | 0.0413 |  |
| *Yixi*_distal | 0.0347 |  | 0.0457 | -0.0542 | 0.019 |  | 0.0498 |  |
| *Mele*_prox | 0.0576 | -0.024 | 0.0449 | 0 | 0.0339 | 0.0676 | 0.0775 | 0.281 |
| *Mele*_mid | 0.0620 | -0.0554 | 0.0459 | 0 | 0.0178 | 0.0529 | 0.0571 | 0.254 |
| *Pavo*_prox | 0.0697 | -0.0258 | 0.0551 | 0 | 0.0297 | 0.0875 | 0.0776 | 0.326 |
| *Pavo*_mid | 0.0724 | -0.0466 | 0.0547 | 0 | 0.0370 | 0.0928 | 0.0760 | 0.372 |
| *Strut*_prox | 0.0898 | 0.0460 | 0.0678 | 0 | 0.0386 | 0.0296 | 0.0851 | 0.140 |
| *Strut*_mid | 0.0618 | 0.0143 | 0.0584 | 0 | 0.0275 | 0.0244 | 0.0721 | 0.140 |
| *Strut*_distal | 0.0492 | 0.00955 | 0.0490 | 0 | 0.0187 | 0.0196 | 0.0579 | 0.105 |
| *Colu*_prox | 0.0917 | -0.0565 | 0.0470 | 0 | 0.0272 | 0.0545 | 0.0806 | 0.336 |
| *Colu*_mid | 0.0829 | -0.112 | 0.0360 | 0 | 0.0365 | 0.0484 | 0.0807 | 0.375 |
| *Colu*_distal | 0.0976 | -0.101 | 0.0438 | 0 | 0.0363 | 0.0413 | 0.0729 |  |
| *Plat*_prox | 0.174 | 0.086 | 0.11 | -0.296 | 0.047 | 0.050 | 0.11 |  |
| *Plat*_mid | 0.0908 | 0.00923 | 0.059 | -0.165 | 0.024 | 0.018 | 0.0916 | 0.132 |
| *Plat*_distal | 0 | 0.00143 | 0.0263 | -0.0688 | 0 | 0.00195 | 0.060 | 0.028 |
| *C.nil*_prox | 0.275 | 0.032 | 0.107 | -0.176 | 0.0657 | 0.0340 | 0.190 | 0.429 |
| *C.nil* _mid | 0.243 | 0.0181 | 0.0772 | -0.117 | 0.0313 | 0.00847 | 0.202 | 0.139 |
| *C*.*nil*_distal | 0.0496 | 0 | 0.0372 | -0.0140 | 0.00718 | 0 | 0.157 | 0.0342 |
| *C.sia*_prox | 0.226 | 0.033 | 0.10 | -0.178 | 0.050 | 0.045 | 0.194 | 0.454 |
| *C.sia*_mid | 0.215 | 0.01 | 0.0747 | -0.128 | 0.0310 | 0.0106 | 0.222 | 0.148 |
| *C.sia*_distal | 0.10 | 0 | 0.039 | -0.0326 | 0.014 | 0 | 0.154 | 0.045 |
| *V.nil*_prox | 0.267 | 0.014 | 0.0606 | -0.135 | 0.0708 | 0.0379 | 0.158 | 0.375 |
| *V.nil*_mid | 0.206 | -0.00034 | 0.0461 | -0.146 | 0.048 | 0.0340 | 0.137 | 0.214 |
| *V.nil*_distal | 0.113 | 0.0073 | 0.0340 | -0.0820 | 0.023 | 0.0280 | 0.118 | 0.122 |
| *V.ko*_prox | 0.258 | -0.0076 | 0.0600 | -0.159 | 0.0880 | 0.0341 | 0.155 | 0.493 |
| *V.ko*_mid | 0.164 | -0.0425 | 0.0357 | -0.115 | 0.0528 | 0.0373 | 0.123 | 0.233 |
| *V.ko*_distal | 0.0517 | -0.0132 | 0.0170 | -0.0297 | 0.017 | 0.0233 | 0.0661 | 0.0955 |
| *Acin*_prox | 0.00927 | 0.0079 | 0.0219 | 0 | 0.00402 | 0.0290 | 0.110 | 0.123 |
| *Acin*_mid | 0 | 0 | 0.0208 | 0 | 0 | 0 | 0.152 | 0.0572 |
| *Acin*_distal | 0 | 0 | 0.011 | 0 | 0 | 0 | 0.0880 | 0.0194 |
| *Macr*_prox | 0 | 0.0287 | 0.111 | -0.130 | 0 | 0.136 | 0.205 | 0.287 |
| *Macr*_mid | 0 | -0.00418 | 0.113 | -0.160 | 0 | 0.0606 | 0.286 | 0.188 |
| *Macr*_distal | 0 | -0.00430 | 0.0570 | -0.0617 | 0 | 0.0299 | 0.140 | 0.0829 |

Table S4. Average values for the three regions of the tail using the complete dataset. ‘Prox’, ‘mid’, and ‘distal’ represent the proximal, middle and distal tail regions respectively. Taxon abbreviations are given in Table S1.

Table S5. Standard deviations for the complete dataset

|  | Height normalised with femur length | | | Depth  normalised  with femur  length | Length normalised with femur length | | | Width normal-ised with femur length |
| --- | --- | --- | --- | --- | --- | --- | --- | --- |
| Taxon | Neural spine | Transverse process | Centrum | Chevron | Neural spine | Transverse process | Centrum | Vertebral |
| *Coel*_prox | 0.00802 | 0.0615 | 0.00835 |  | 0.00846 | 0.0191 | 0.0179 | 0 |
| *Coel*_mid | 0.0178 | 0.0187 | 0.0160 |  | 0.0271 | 0.0272 | 0.0374 |  |
| *Coel*_distal | 0 | 0 | 0.00831 |  | 0 | 0 | 0.0515 |  |
| *Dilo*_prox | 0.0387 | 0.0503 | 0.0169 | 0.0115 | 0 | 0.0215 | 0.00521 |  |
| *Dilo*_mid | 0.0267 | 0.00805 | 0.00500 |  | 0.00504 | 0.00724 | 0.00394 | 0.00453 |
| *Dilo*_distal | 0 | 0 | 0.00751 |  | 0 | 0 | 0.00470 | 0.00779 |
| *Trex*_prox | 0.0272 | 0.0279 | 0.0174 | 0.0267 | 0.0115 | 0.0127 | 0.00977 | 0.0204 |
| *Trex*_mid | 0.0230 | 0.0180 | 0.00979 | 0.0169 | 0.029 | 0.0134 | 0.0118 | 0.0358 |
| *Trex*_distal | 0 | 0 | 0 | 0 | 0 | 0 | 0 | 0 |
| *Jura*_prox | 0.0623 | 0 | 0.0106 | 0.0447 | 0.0246 | 0.00555 | 0.0115 |  |
| *Jura*_mid | 0 |  | 0.00501 | 0.00113 | 0 |  | 0.00804 |  |
| *Jura*_distal | 0 |  | 0.00273 |  | 0 |  | 0.00385 |  |
| *Comp*_prox | 0.0453 |  | 0.00614 | 0.0297 | 0.0340 |  | 0.0110 |  |
| *Ornit*_prox |  | 0.0259 | 0.0163 |  |  | 0 | 0.0172 |  |
| *Ornit*_mid | 0.00357 | 0 | 0.012 | 0.00420 | 0.0315 | 0 | 0.00780 | 0.0110 |
| *Ornit*_distal |  | 0 | 0.00129 |  |  | 0 | 0.0133 | 0.00492 |
| *Shuv*_prox | 0.0276 | 0.0133 | 0.0162 | 0.0120 | 0.0111 | 0.00135 | 0.0383 |  |
| *Shuv*_mid | 0.00701 | 0.0215 | 0.00557 |  | 0.0079 | 0 | 0.0793 | 0.00222 |
| *Citi*_prox |  |  |  | 0 |  | 0.00400 |  | 0.0296 |
| *Citi*_mid | 0.0162 | 0.00662 | 0.00253 | 0.0243 | 0.00341 | 0.00353 | 0.00339 | 0.0263 |
| *Citi*_distal | 0.0101 | 0.0027 | 0.00729 | 0.0122 | 0.0197 | 0.0229 | 0.0125 | 0.0399 |
| *Khaa*_prox | 0 | 0 | 0 |  | 0.00858 | 0.00794 | 0 | 0.0317 |
| *Khaa*_mid | 0.00302 | 0.00161 | 0.00243 |  | 0.00723 | 0.00642 |  | 0.0182 |
| *Khaa*_distal | 0.00927 | 0.00679 | 0.00245 |  | 0.0096 | 0.0233 | 0.00416 | 0 |
| *Caud*_prox | 0.0139 |  | 0.0117 | 0.0391 | 0.00912 |  | 0.0234 |  |
| *Caud*_mid | 0.00468 |  | 0.00230 | 0.0430 | 0.00416 | 0.00270 | 0.00571 |  |
| *Caud*_distal | 0 | 0 | 0.015 | 0 | 0 | 0 | 0.0164 | 0.00923 |
| *Mei*_prox | 0.0126 | 0.017 | 0.00353 | 0.0155 | 0.0384 | 0.00994 | 0.0506 | 0.0340 |
| *Mei*_mid | 0 | 0 | 0.00453 | 0.00686 | 0 | 0 | 0.00697 | 0.00535 |
| *Mei*_distal | 0 | 0 | 0.0055 | 0.00412 | 0 | 0 | 0.00569 | 0.0102 |
| *Sino*_prox | 0 | 0.0137 | 0.00402 | 0.0184 | 0 | 0.0157 | 0.0380 | 0.0678 |
| *Sino*_mid | 0 | 0 | 0.00495 | 0.00273 | 0 | 0 | 0.00455 | 0 |
| *Sino*_distal | 0 | 0 | 0.00489 | 0.00502 | 0 | 0 | 0.0126 | 0.00197 |
| *Sinu*_prox | 0 | 0.0446 | 0.0194 | 0.0554 | 0 | 0.0258 | 0.0251 | 0.0703 |
| *Sinu*_mid | 0 | 0 | 0.00385 | 0.00359 | 0 | 0 | 0.00712 | 0.00771 |
| *Sinu*_distal | 0 | 0 | 0.0125 | 0.00535 | 0 | 0 | 0.0218 | 0.00689 |
| *Maha*_prox | 0.00496 | 0.018 | 0.00607 | 0.0108 | 0.0112 | 0.00821 | 0.00960 | 0.00984 |
| *Maha* _mid | 0.00440 | 0.0056 | 0.00248 | 0 | 0.00109 | 0.016 | 0.00283 | 0.00605 |
| *Dein*_prox | 0.00661 | 0.00902 | 0.00279 | 0.00397 | 0.0410 | 0 | 0.0106 | 0.00501 |
| *Dein*_mid | 0.0142 | 0 | 0.00538 | 0.00361 | 0.0616 | 0 | 0.00468 | 0.00411 |
| *Dein*_distal | 0 | 0 | 0.0061 | 0.0119 | 0 | 0 | 0.0194 | 0.0102 |
| *Velo*_prox | 0.0318 | 0.011 | 0.0118 | 0.0139 | 0.0109 | 0.019 | 0.0209 | 0.0819 |
| *Velo*_mid | 0.00681 | 0 | 0.00945 | 0.0132 | 0.0103 | 0 | 0.0108 | 0.00796 |
| *Vel*_F_prox | 0 |  | 0.00447 | 0.0504 |  | 0.0103 | 0.0250 |  |
| *Vel*_F_mid |  | 0 |  | 0.00806 |  | 0 | 0.0111 |  |
| *Vel*_F_distal |  | 0 |  | 0 |  | 0 | 0.0451 |  |
| *Bamb*_prox | 0.0199 | 0.017 | 0.00571 | 0.0196 | 0.00337 | 0.0440 | 0.0412 | 0.00599 |
| *Bamb*_mid |  | 0 | 0 |  |  | 0 |  |  |
| *Drom* _prox |  |  |  | 0 |  | 0.00645 |  |  |
| *Drom*_mid | 0 |  | 0.00430 |  | 0 |  | 0.0306 |  |
| *Drom*_distal | 0 |  | 0 |  | 0 |  | 0.170 |  |
| *M.gui*_prox |  |  | 0.00206 |  |  |  | 0.0186 |  |
| *M.gui*_mid |  |  | 0.00723 |  |  |  | 0.0185 |  |
| *M.gui*_distal |  |  |  |  |  |  | 0 |  |
| *M.zha*_prox |  | 0 |  |  |  | 0 | 0.0672 |  |
| *M.zha*_mid |  | 0 |  |  |  | 0 | 0.00661 |  |
| *M.zha*_distal |  | 0 |  |  |  | 0 | 0.0451 |  |
| *Epid*_prox |  |  |  |  |  | 0.00942 | 0.0120 | 0 |
| *Epid*_mid |  |  |  |  |  |  | 0.0181 |  |
| *Epid*_distal |  |  |  |  |  |  | 0.0321 |  |
| *Ar*.B_prox | 0.00205 | 0.0083 | 0.00683 | 0.044 | 0.00130 | 0.0130 | 0.0523 |  |
| *Ar*.B_mid |  | 0 | 0.000952 | 0 |  | 0 | 0.00479 |  |
| *Ar*.B_distal |  | 0 | 0 |  |  | 0 | 0.0116 |  |
| *Ar*.J_prox | 0 | 0.0052 | 0.0095 | 0.0141 | 0 | 0.0185 | 0.0500 |  |
| *Ar*.J_mid | 0 | 0 | 0.00506 | 0.0057 | 0 | 0 | 0.00387 |  |
| *Ar*.J_distal | 0 | 0 | 0.00377 | 0.0055 | 0 | 0 | 0.0435 | 0.0019 |
| *Ar*.H_prox | 0 | 0.0029 | 0.00937 | 0.0249 | 0 | 0.013 | 0.0393 |  |
| *Ar*.H_mid | 0 | 0 | 0.00122 | 0 | 0 | 0 | 0.0243 |  |
| *Ar*.H_distal | 0 | 0 |  |  | 0 | 0 | 0.456 |  |
| *Jeho*_prox | 0.0451 | 0.0002 | 0.00309 | 0.0129 | 0.0145 | 0.0210 | 0.0463 | 0 |
| *Jeho*_mid | 0 | 0 | 0.0064 | 0.00559 | 0 | 0 | 0.00694 |  |
| *Jeho*_distal | 0 | 0 | 0.0088 | 0.0050 | 0 | 0 | 0.0240 | 0 |
| *Sape*_prox |  |  |  |  | 0 | 0.0061 | 0.00430 | 0.000902 |
| *Sape*_mid |  |  |  | 0 |  | 0.00329 | 0.00222 | 0.00295 |
| *Sape*_distal |  |  |  |  |  | 0.00809 | 0.0132 | 0.00825 |
| *Conf*_prox |  |  |  |  |  | 0 | 0.00618 |  |
| *Hong*_prox | 0 |  | 0 | 0 | 0 |  | 0 |  |
| *Hong*_mid | 0 |  | 0.010 |  | 0 |  | 0.013 |  |
| *Yixi*_prox | 0 | 0.0045 | 0.00281 |  | 0 | 0.0044 | 0.00660 |  |
| *Yixi*_mid | 0 |  | 0.00951 | 0.0112 | 0 |  | 0 |  |
| *Yixi*_distal | 0 |  | 0 | 0 | 0 |  | 0 |  |
| *Mele*_prox | 0.00440 | 0.025 | 0.00776 | 0 | 0.0133 | 0.0135 | 0.0138 | 0.00941 |
| *Mele*_mid | 0 | 0.0218 | 0.00298 | 0 | 0.00411 | 0.00765 | 0.00699 | 0.0260 |
| *Pavo*_prox | 0.00469 | 0.00630 | 0.000910 | 0 | 0.00359 | 0.0000479 | 0.00254 | 0.0167 |
| *Pavo*_mid | 0.000192 | 0.00244 | 0.00249 | 0 | 0.00311 | 0.00201 | 0.00632 | 0.0195 |
| *Strut*_prox | 0.0117 | 0.00932 | 0.00357 | 0 | 0.00850 | 0.00659 | 0.00969 | 0.00226 |
| *Strut*_mid | 0.00864 | 0.00364 | 0.00152 | 0 | 0.00293 | 0.000775 | 0.00174 | 0.00557 |
| *Strut*_distal | 0.00262 | 0.00354 | 0.00296 | 0 | 0.00111 | 0.00509 | 0.00304 | 0.0196 |
| *Colu*_prox | 0.00672 | 0.00910 | 0.000611 | 0 | 0.00476 | 0.0125 | 0.00269 | 0.0107 |
| *Colu*_mid | 0.0101 | 0.0209 | 0.000855 | 0 | 0.00354 | 0.0134 | 0.000366 | 0 |
| *Colu*_distal | 0.0177 | 0 | 0.00427 | 0 | 0.00110 | 0 | 0.0140 |  |
| *Plat*_prox | 0.0332 | 0.012 | 0.023 | 0.0455 | 0.0038 | 0.013 | 0.0090 |  |
| *Plat*_mid | 0.0160 | 0.0122 | 0.012 | 0.0390 | 0.0094 | 0.012 | 0.0133 | 0.0500 |
| *Plat*_distal | 0 | 0.00352 | 0.00687 | 0.0270 | 0 | 0.00315 | 0.0067 | 0.014 |
| *C.nil*_prox | 0.00490 | 0.014 | 0.0110 | 0.0905 | 0.0122 | 0.0129 | 0.0150 | 0.0961 |
| *C.nil*_mid | 0.0334 | 0.0264 | 0.00833 | 0.0231 | 0.00813 | 0.0124 | 0.00360 | 0.0782 |
| *C*.*nil*_distal | 0.0467 | 0 | 0.0168 | 0.0229 | 0.00661 | 0 | 0.0367 | 0.0184 |
| *C*.*sia*_prox | 0.00886 | 0.010 | 0.014 | 0.0644 | 0.0089 | 0.012 | 0.0191 | 0.0477 |
| *C*.*sia*_mid | 0.0297 | 0.01 | 0.00679 | 0.0210 | 0.00262 | 0.0172 | 0.00351 | 0.0833 |
| *C*.*sia*_distal | 0.085 | 0 | 0.017 | 0.0461 | 0.011 | 0 | 0.0538 | 0.022 |
| *V.nil*_prox | 0.0133 | 0.0063 | 0.00491 | 0.0765 | 0.0135 | 0.00656 | 0.0115 | 0.0695 |
| *V.nil*_mid | 0.0313 | 0.0094 | 0.00420 | 0.0173 | 0.0057 | 0.00501 | 0.00821 | 0.0369 |
| *V.nil*_distal | 0.0213 | 0.0053 | 0.00395 | 0.0194 | 0.0082 | 0.00389 | 0.00777 | 0.0213 |
| *V.ko*_prox | 0.0127 | 0.015 | 0.0128 | 0.0552 | 0.0139 | 0.00633 | 0.0128 | 0.0710 |
| *V.ko*_mid | 0.0392 | 0.0101 | 0.00617 | 0.0149 | 0.0133 | 0.00387 | 0.0125 | 0.0452 |
| *V.ko*_distal | 0.0252 | 0.0111 | 0.00580 | 0.0155 | 0.010 | 0.00787 | 0.0191 | 0.0379 |
| *Acin*_prox | 0.0227 | 0.0054 | 0.000967 | 0 | 0.00984 | 0.00782 | 0.0204 | 0.0234 |
| *Acin*_mid | 0 | 0 | 0.00104 | 0 | 0 | 0 | 0.00461 | 0.0108 |
| *Acin*_distal | 0 | 0 | 0.0050 | 0 | 0 | 0 | 0.0348 | 0.00958 |
| *Macr*_prox | 0 | 0.0148 | 0.00270 | 0.104 | 0 | 0.0368 | 0.0363 | 0.0139 |
| *Macr*_mid | 0 | 0.0151 | 0.00716 | 0.0481 | 0 | 0.00701 | 0.0127 | 0.0265 |
| *Macr*_distal | 0 | 0.00769 | 0.0281 | 0.0682 | 0 | 0.0188 | 0.0758 | 0.0474 |

Table S5. Standard deviations for the complete dataset (Table S3). Taxon abbreviations are given in Table S1.

Table S6. Vertebral parameter loadings on the first three principal components

| **A**  Parameter | PC1 | PC1 % loading | PC2 | PC2 % loading | PC3 | PC3 % loading |
| --- | --- | --- | --- | --- | --- | --- |
| Neural spine height | -0.50 | 24 | 0.37 | 16 | -0.68 | 29 |
| Transverse process height | -0.044 | 2.1 | 0.11 | 5.0 | 0.037 | 1.6 |
| Centrum height | -0.11 | 5.6 | 0.10 | 4.4 | 0.25 | 11 |
| Chevron depth | 0.29 | 14 | -0.62 | 27 | -0.24 | 10 |
| Neural spine length | -0.13 | 6.3 | -0.0084 | 0.37 | -0.16 | 6.6 |
| Transverse process length | -0.076 | 3.7 | -0.12 | 5.1 | 0.20 | 8.5 |
| Centrum length | -0.10 | 5.0 | 0.41 | 18 | 0.54 | 23 |
| Vertebral width | -0.79 | 39 | -0.52 | 23 | 0.24 | 10 |

| **B** Parameter | PC1 | PC1 % loading | PC2 | PC2 % loading | PC3 | PC3 % loading |
| --- | --- | --- | --- | --- | --- | --- |
| Neural spine height | -0.40 | 18 | 0.34 | 13 | -0.18 | 9.0 |
| Transverse process height | -0.096 | 4.4 | 0.54 | 21 | -0.21 | 10 |
| Centrum height | -0.18 | 8.4 | 0.33 | 13 | 0.010 | 0.52 |
| Chevron depth | 0.12 | 5.6 | -0.46 | 18 | -0.084 | 4.2 |
| Neural spine length | -0.16 | 7.3 | 0.21 | 8.0 | -0.22 | 11 |
| Transverse process length | -0.22 | 9.9 | -0.043 | 1.7 | -0.11 | 5.3 |
| Centrum length | 0.17 | 8.0 | 0.34 | 13 | 0.86 | 43 |
| Vertebral width | -0.83 | 38 | -0.33 | 13 | 0.35 | 17 |

| **C** Parameter | PC1 | PC1 % loading | PC2 | PC2 % loading | PC3 | PC3 % loading |
| --- | --- | --- | --- | --- | --- | --- |
| Neural spine height | -0.51 | 20 | 0.033 | 1.9 | -0.12 | 6.5 |
| Transverse process height | -0.32 | 13 | 0.11 | 6.3 | -0.014 | 0.75 |
| Centrum height | -0.31 | 12 | 0.19 | 11 | -0.039 | 2.0 |
| Chevron depth | 0.32 | 13 | -0.12 | 6.5 | -0.23 | 12 |
| Neural spine length | -0.24 | 9.3 | -0.15 | 8.5 | -0.87 | 45 |
| Transverse process length | -0.18 | 7.0 | -0.19 | 11 | -0.12 | 6.1 |
| Centrum length | 0.050 | 2.0 | 0.93 | 52 | -0.16 | 8.2 |
| Vertebral width | -0.59 | 24 | -0.055 | 3.0 | 0.38 | 20 |

Table S6: Vertebral parameter loadings on the first three principal components: A, complete dataset, B, theropod dataset (outgroups excluded), and C, non-avian theropod dataset (no outgroups and Avialae/Aves).

Reconstructed nodal values for tail parameters (from mapping data in Table S4 and S7 over EBL and SBL tree topologies) are shown in Tables S8-10.

Table S7. Size-normalised tail lengths and caudal counts for the amniotes studied

| Taxon | Tail length | Caudal count |
| --- | --- | --- |
| *Coelophysis bauri*, UCMP cast | 7.01 (Linear interpolation was used for caudals 6 and 35.) | 39 (Caudal 39 is the last one preserved but it is probably not the last caudal in the tail. This is because there is no completely preserved tail in any close phylogenetic relative that might help to estimate caudal count more accurately [4].) |
| *Dilophosaurus wetherilli*, UCMP V4214/37302 | 5.91 (Linear interpolation was used for caudals 3 and 14, whilst linear extrapolation was used for caudals 35-44.) | 44 (Caudal 44 is the last one preserved but it is probably not the last caudal in the tail. This is because there is no completely preserved tail in any close phylogenetic relative that might help to estimate caudal count more accurately [5,6].) |
| *Tyrannosaurus rex*, OUMNH Stan cast | 4.99 (Linear interpolation was used for caudal 25, whilst linear extrapolation was used for caudals 1-3 and 27-47.) | 47 (from ‘Sue’ specimen, FMNH PR2081 [1]) |
| *Juravenator starki*, JME Sch 200 | 7.57 (Linear interpolation was used for caudals 14, 26 and 35, whilst linear extrapolation was used for caudals 1 and 44-55.) | 55 (from *Sinosauropterx prima*, NGMC 2123 and NIGP 127586 [7]) |
| *Compsognathus longipes*, BSPG AS I 563 | 8.57 (Linear interpolation was used for caudals 6-8, whilst linear extrapolation was used for caudals 1-2 and 10-55.) | 55 (from *Sinosauropterx prima*, NGMC 2123 and NIGP 127586 [7]) |
| *Ornitholestes hermanni*, AMNH 619 | 6.34 (Linear interpolation was used for caudals 8-12 and 25, whilst linear extrapolation was used for caudals 31-44.) | 44 (from [8]) |
| *Shuvuuia deserti*, MPC 100/1305 | 4.98 (Linear extrapolation was used for caudals 1-2 and 15-35. The latter used the 20% decrease in centrum length observed along the distal tail of MPC 100/20 [9].) | 35 (from MPC 100/20 [9]) |
| *Citipati osmolskae*, MPC 100/978 | 2.49 (Linear interpolation was used for caudals 14, 15 and 18-20, whilst linear extrapolation was used for caudals 1-12. | 32 (complete tail) |
| *Khaan mckennai*, MPC 100/1127 | 1.87 (Linear interpolation was used for caudals 2-20, whilst linear extrapolation was used for caudals 25-27.) | 27 (complete tail) |
| *Caudipteryx zoui*, IVPP V12430 | 1.55 (Linear interpolation was used for caudals 4 and 6.) | 24 (complete tail) |
| *Mei long*, IVPP V12733 | 3.26 (Linear interpolation was used for caudal 4, whilst linear extrapolation was used for caudals 23-25.) | 25 (Broken distal tail fragments were reassembled to reconstruct the originally complete tail)*.* |
| *Sinornithoides youngi*, IVPP V9612 | 4.29 (Linear interpolation was used for caudals 3-6, whilst linear extrapolation was used for caudals 27-32.) | 32 (Caudal 27 is the last caudal preserved. Although *Sinusonasus* probably had 30 caudals, linear extrapolation of the *Sinornithoides* tail data suggests that it probably had two more caudals than *Sinusonasus.* |
| *Sinusonasus magnodens*, IVPP V11527 | 4.44 (Linear interpolation was used for caudals 4, 7 and 13.) | 30 (from [10]) |
| *Mahakala osmolskae*, MPC 100/1033 | 6.15 (Linear interpolation was used for caudal 2, whilst linear extrapolation was used for caudals 18-40.) | 40 (from [11]) |
| *Deinonychus antirrhopus*, YPM 5203 | 4.78 (Linear interpolation was used for caudals 10, 15 and 27, whilst linear extrapolation was used for caudals 1-6 and 36-40.) | 40 (from [11]) |
| *Velociraptor mongoliensis*, MPC 100/985 | 4.62 (Linear interpolation was used for caudals 2, 3, 6, 15, 16, 18 and 19, whilst linear extrapolation was used for caudals 26-40.) | 40 (from [11]) |
| *Velociraptor mongoliensis*, MPC fighting specimen | 4.93 (Linear extrapolation was used for caudals 31-40.) | 40 (from [11]) |
| *Bambiraptor feinbergi*, AMNH 001 | 7.68 (Linear extrapolation was used for caudals 14-40.) | 40 (from [11]) |
| Undescribed Liaoning dromaeosaurid*,* IVPP 2008.5 | 11.0 (Linear extrapolation was used for caudals 1-6 and 16-24.) | 24 (complete tail) |
| *Microraptor gui*, IVPP V13352 | 6.24 (Linear interpolation was used for caudal 10, whilst linear extrapolation was used for caudals 15-23.) | 23 (complete tail, contra to the 26 caudals estimated by [12]) |
| *Microraptor zhaoianus*, CAGS 20-8-001 | 4.67 (Complete set of measurements from [2].) | 26 (complete tail) |
| *Epidexipteryx hui*, IVPP V15471A | 1.38 (Linear interpolation was used for caudals 4 and 6.) | 16 (complete tail) |
| *Archaeopteryx lithographica*, BSPG S6 | 3.31 (Complete set of measurements.) | 21 (complete tail) |
| *Archaeopteryx lithographica*, JM2257 | 3.72 (Linear interpolation was used for caudal 4, whilst linear extrapolation was used for caudal 23.) | 23 (complete tail) |
| *Archaeopteryx lithographica*, HMN 1880 | 3.34 (Linear interpolation was used for caudals 13, 18 and 19.) | 21 (complete tail) |
| *Jeholornis prima*, IVPP V13274 | 5.01 (Linear interpolation was used for caudals 14-15, whilst linear extrapolation was used for caudal 1.) | 22 (complete tail) |
| *Sapeornis chaoyangensis*, IVPP V13276 | 2.12 (Linear interpolation was used for caudals 3, 7 and 8.) | 9 (complete tail) |
| *Confuciusornis sanctus*, IVPP V11374 | 0.914 (Complete set of measurements.) | 4 (complete tail) |
| *Hongshanornis longicresta*, IVPP V14533 | 0.24 (Complete set of measurements.) | 3 (complete tail) |
| *Yixianornis grabaui*, IVPP V12631 | 0.60 (Linear interpolation was used for caudals 3-6.) | 8 (complete tail) |
| *Meleagris gallopavo*, NHMUK 1898.5.30.1 | 0.550 (Complete set of measurements.) | 5 (complete tail) |
| *Pavo muticus*, NHMUK 1847.12.11.13 | 0.619 (Complete set of measurements.) | 4 (complete tail) |
| *Struthio camelus*, NHMUK S/2006.11.1 | 0.793 (Complete set of measurements.) | 9 (complete tail) |
| *Columba livia*, UCL GMZ | 0.601 (Complete set of measurements.) | 6 (complete tail) |
| *Plateosaurus engelhardti*, MB.R. 4430 | 4.0 (Linear interpolation was used for caudal 43, whilst linear extrapolation was used for caudals 45-50.) | 50 (from [13]) |
| *Crocodylus niloticus*, CMZ | 6.87 (Complete set of measurements.) | 38 (complete tail) |
| *Crocodylus siamensis*, X 226 UCL GMZ | 6.65 (Linear interpolation was used for caudal 31.) | 36 (complete tail) |
| *Varanus niloticus*, UCL GMZ | 8.32 (Linear extrapolation was used for caudals 60-62.) | 62 (complete tail) |
| *Varanus komodoensis*, CMZ | 7.02 (Linear extrapolation was used for caudals 25 and 67.) | 70 (complete tail) |
| *Acinonyx jubatus*, CMZ K.5441 | 2.50 (Complete set of measurements.) | 23 (complete tail) |
| *Macropus giganteus*, CMZ A12.17/1 | 4.20 (Complete set of measurements.) | 22 (complete tail) |

Table S7. Size-normalised tail lengths for all of the taxa studied (sum of all centrum lengths and, when applicable, the length of a completely fused pygostyle or the sum of element lengths within a partially ankylosed pygostyle) and the caudal counts of these tails (number of caudal vertebrae excluding the pygostyle). Missing values were filled artificially using linear interpolation and extrapolation.

Table S8. Vertebral parameter nodal values reconstructed using EBL assumptions

|  | Neural spine height | | | Transverse process height | | | Centrum height | | | Chevron depth | | |
| --- | --- | --- | --- | --- | --- | --- | --- | --- | --- | --- | --- | --- |
| Node | Prox | Mid | Dist | Prox | Mid | Dist | Prox | Mid | Dist | Prox | Mid | Dist |
| 1 | 0.109 | 0.0773 | 0.0289 | 0.023 | -0.00402 | -0.00181 | 0.0727 | 0.0610 | 0.032 | -0.115 | -0.102 | -0.0392 |
| 2 | 0.179 | 0.129 | 0.0482 | 0.026 | -0.0053 | -0.0016 | 0.0771 | 0.0570 | 0.0308 | -0.148 | -0.117 | -0.0447 |
| 3 | 0.193 | 0.143 | 0.045 | 0.045 | 0.004 | -4.57E-04 | 0.093 | 0.0636 | 0.033 | -0.182 | -0.123 | -0.0428 |
| 4 | 0.168 | 0.0997 | 0.0210 | 0.072 | 0.00704 | 3.71E-04 | 0.10 | 0.062 | 0.0318 | -0.219 | -0.129 | -0.0540 |
| 5 | 0.138 | 0.0653 | 0.0183 | 0.0859 | 0.00772 | 1.39E-04 | 0.0995 | 0.0637 | 0.0360 | -0.180 | **-0.0989** | **-0.0504** |
| 6 | 0.145 | 0.0607 | 0.0277 | 0.0907 | 0.00944 | 1.17E-07 | 0.110 | 0.0709 | 0.0443 | -0.148 | -0.0689 | -0.0468 |
| 7 | **0.119** | 0.0411 | **0.0196** | 0.0732 | 0.00700 | -1.39E-04 | 0.0936 | 0.0593 | 0.0332 | **-0.133** | -0.0481 | **-0.0410** |
| 8 | 0.0941 | 0.0308 | 0.0116 | 0.0652 | **0.0115** | **-0.000416** | 0.0799 | 0.0580 | 0.0390 | -0.118 | -0.0544 | **-0.0351** |
| 9 | 0.0760 | 0.0360 | **0.00924** | 0.0513 | 0.0161 | **-0.000694** | 0.0720 | 0.0537 | **0.0348** | -0.0893 | **-0.0547** | **-0.0293** |
| 10 | 0.0659 | 0.0242 | 0.00694 | 0.0356 | 0.00755 | -0.000972 | 0.0638 | 0.0501 | 0.031 | -0.0957 | -0.0550 | -0.0235 |
| 11 | 0.0459 | 0.0150 | 0.00421 | 0.024 | 0.0036 | -8.23E-04 | 0.0557 | 0.0456 | 0.031 | -0.0744 | -0.0400 | -0.0232 |
| 12 | 0.0311 | 0.00883 | 0.00352 | 0.014 | 0.00109 | -0.00107 | 0.053 | 0.0432 | 0.0323 | -0.063 | -0.031 | -0.022 |
| 13 | **0.0321** | **0.00859** | **0.00518** | **0.0105** | **-0.000615** | **-0.00213** | **0.0563** | **0.0447** | **0.0313** | **-0.0514** | **-0.0287** | **-0.0204** |
| 14 | 0.0331 | 0.00835 | 0.00685 | 0.007 | -0.00232 | -0.00318 | 0.0598 | 0.0462 | 0.030 | -0.0403 | -0.0260 | -0.018 |
| 15 | **0.0354** | **0.0165** | **0.0154** | **0.00955** | **-0.00634** | **-0.00741** | **0.0601** | **0.0472** | **0.0338** | **-0.0359** | **-0.0301** | **-0.0217** |
| 16 | **0.0377** | **0.0246** | **0.0239** | **0.0124** | **-0.0104** | **-0.0116** | **0.0603** | **0.0482** | **0.0373** | **-0.0315** | -0.0343 | **-0.0251** |
| 17 | 0.040 | 0.033 | **0.0324** | **0.0152** | **-0.0144** | **-0.0159** | 0.0606 | 0.049 | **0.0407** | -0.027 | **-0.0311** | **-0.0284** |
| 18 | 0.0633 | 0.0455 | 0.0409 | 0.018 | **-0.0184** | **-0.0201** | 0.0633 | 0.0522 | 0.0441 | **-0.0167** | -0.0279 | -0.0318 |
| 19 | 0.0775 | 0.0591 | 0.0555 | 0.0140 | -0.0224 | -0.0243 | 0.0614 | 0.0521 | 0.0460 | -0.00637 | -0.0106 | -0.0127 |
| 20 | 0.0794 | 0.0701 | **0.0555** | -0.0222 | -0.0632 | **-0.0243** | 0.0532 | 0.0456 | **0.0460** | -0.00239 | -0.00399 | **-0.0127** |
| 21 | 0.0689 | 0.0682 | **0.0555** | -0.024 | -0.0551 | **-0.0243** | 0.0511 | 0.0487 | **0.0460** | -7.96E-04 | -0.00133 | **-0.0127** |

|  | Neural spine length | | | Transverse process length | | | Centrum length | | | Vertebral width | | |
| --- | --- | --- | --- | --- | --- | --- | --- | --- | --- | --- | --- | --- |
| Node | Prox | Mid | Dist | Prox | Mid | Dist | Prox | Mid | Dist | Prox | Mid | Dist |
| 1 | 0.0315 | 0.0184 | 0.00635 | 0.0634 | 0.0279 | 0.0146 | 0.158 | 0.186 | 0.111 | 0.290 | 0.149 | 0.0625 |
| 2 | 0.0512 | 0.0306 | 0.011 | 0.0506 | 0.0262 | 0.0144 | 0.158 | 0.164 | 0.108 | 0.347 | 0.166 | 0.0701 |
| 3 | 0.052 | 0.0297 | 0.0085 | 0.047 | 0.0183 | 0.00669 | 0.158 | 0.165 | 0.117 | 0.347 | 0.145 | 0.052 |
| 4 | 0.049 | 0.028 | 0.00510 | 0.050 | 0.016 | 0.00344 | 0.14 | 0.134 | 0.10 | **0.283** | 0.125 | 0.042 |
| 5 | 0.0482 | 0.0296 | 0.00677 | 0.0514 | 0.0125 | 0.00167 | 0.142 | 0.147 | 0.123 | 0.219 | 0.0987 | 0.0457 |
| 6 | 0.0606 | 0.044 | 0.0130 | 0.0502 | 0.0102 | 0.00101 | 0.141 | 0.145 | 0.124 | 0.223 | 0.0972 | 0.0576 |
| 7 | **0.0525** | 0.0741 | **0.00970** | 0.0352 | 0.00844 | 0.00136 | 0.144 | 0.156 | 0.141 | **2.19E-01** | 0.0820 | 0.0468 |
| 8 | 0.0444 | 0.0433 | 0.00644 | 0.0403 | **0.0151** | **0.00308** | 0.143 | 0.143 | 0.136 | **2.15E-01** | **0.0843** | **0.0441** |
| 9 | 0.0369 | 0.0341 | **0.00640** | 0.0391 | 0.0217 | **0.00479** | 0.143 | 0.128 | **0.124** | **2.11E-01** | 0.0866 | **0.0415** |
| 10 | 0.0302 | 0.0262 | 0.0064 | 0.0350 | 0.0208 | 0.00650 | 0.121 | 0.127 | 0.112 | 0.208 | 0.110 | 0.0388 |
| 11 | 0.0251 | 0.0236 | 0.00353 | 0.031 | 0.011 | 0.00387 | 0.123 | 0.157 | 0.137 | 0.167 | 0.0917 | 0.0333 |
| 12 | 0.0160 | 0.0122 | 0.00240 | 0.028 | 0.00640 | 0.00311 | 0.122 | 0.172 | 0.142 | **0.176** | **0.101** | 0.032 |
| 13 | **0.0144** | **0.00897** | **0.00288** | 0.0292 | **0.00629** | **0.00468** | 0.117 | 0.154 | 0.121 | 0.184 | **0.110** | **0.0362** |
| 14 | 0.0128 | 0.00572 | 0.00336 | 0.0211 | 0.00619 | 0.00626 | 0.153 | 0.182 | 0.139 | 0.127 | **0.119** | 0.041 |
| 15 | **0.0138** | **0.00818** | **0.0072** | 0.019 | **0.0123** | **0.0141** | 0.0990 | **0.135** | **0.103** | **0.136** | **0.129** | **0.0743** |
| 16 | 0.015 | **0.0106** | **0.0110** | 0.020 | 0.0184 | 0.0219 | 0.0758 | 0.0876 | 0.0675 | 0.145 | 0.138 | 0.108 |
| 17 | 0.017 | 0.013 | **0.0149** | **0.0237** | **0.0236** | **0.0233** | 0.0657 | 0.063 | **0.0626** | **0.163** | **0.161** | **0.108** |
| 18 | 0.023 | 0.020 | 0.019 | 0.028 | **0.0289** | **0.0246** | 0.0714 | 0.0570 | 0.0578 | **0.181** | **0.184** | **0.108** |
| 19 | 0.0305 | 0.0261 | 0.0222 | 0.0371 | 0.0342 | 0.0259 | 0.0785 | 0.0670 | 0.0608 | 0.199 | 0.206 | **0.108** |
| 20 | 0.0296 | 0.0303 | **0.0222** | 0.0537 | 0.0492 | **0.0259** | 0.0790 | 0.0720 | **0.0608** | 0.277 | 0.296 | **0.108** |
| 21 | 0.0311 | 0.0284 | **0.0222** | 0.0696 | 0.0650 | **0.0259** | 0.0780 | 0.0684 | **0.0608** | 0.295 | 0.307 | **0.108** |

Table S8. Reconstructed nodal values for the vertebral parameters using EBL assumptions (interpolated values in bold font). ‘Prox’, ‘Mid’ and ‘Dist’ represent the proximal, middle and distal tail regions. Node numbers correspond to those in Figure 1.

Table S9. Reconstructed nodal values of the vertebral parameters by adopting SBL assumptions

|  | Neural spine height | | | Transverse process height | | | Centrum height | | | Chevron depth | | |
| --- | --- | --- | --- | --- | --- | --- | --- | --- | --- | --- | --- | --- |
| Node | Prox | Mid | Dist | Prox | Mid | Dist | Prox | Mid | Dist | Prox | Mid | Dist |
| 1 | 0.106 | 0.0627 | 0.0155 | 0.050 | 0.00199 | -7.61E-04 | 0.0813 | 0.0604 | 0.032 | -0.154 | -0.111 | -0.0439 |
| 2 | 0.141 | 0.0847 | 0.0209 | 0.062 | 0.0034 | -2.7E-04 | 0.0866 | 0.0581 | 0.0308 | -0.184 | -0.122 | -0.0484 |
| 3 | 0.141 | 0.0847 | 0.021 | 0.062 | 0.003 | -2.74E-04 | 0.087 | 0.0581 | 0.031 | -0.184 | -0.122 | -0.0484 |
| 4 | 0.121 | 0.0568 | 0.00610 | 0.078 | 0.00570 | 2.31E-04 | 0.090 | 0.057 | 0.0301 | -0.202 | -0.125 | -0.0530 |
| 5 | 0.121 | 0.0567 | 0.00610 | 0.0783 | 0.00570 | 2.31E-04 | 0.0895 | 0.0568 | 0.0301 | -0.202 | **-0.0854** | **-0.0410** |
| 6 | 0.0569 | 0.0174 | 0.00505 | 0.0446 | 0.00156 | -7.64E-04 | 0.0695 | 0.0530 | 0.0372 | -0.0989 | -0.0460 | -0.0289 |
| 7 | **0.0569** | 0.0174 | **0.00505** | 0.0446 | 0.00156 | -7.64E-04 | 0.0695 | 0.0530 | 0.0372 | **-0.0989** | -0.0459 | **-0.0289** |
| 8 | 0.0569 | 0.0174 | 0.00504 | 0.0446 | **0.00156** | **-7.65E-04** | 0.0694 | 0.0530 | 0.0372 | -0.0989 | -0.0459 | **-0.0289** |
| 9 | 0.0568 | 0.0174 | **0.00504** | 0.0445 | 0.00156 | **-7.66E-04** | 0.0694 | 0.0530 | **0.0372** | -0.0988 | **-0.0459** | **-0.0289** |
| 10 | 0.0568 | 0.0174 | 0.00504 | 0.0445 | 0.00155 | -7.7E-04 | 0.0694 | 0.0530 | 0.037 | -0.0988 | -0.0459 | -0.0289 |
| 11 | 0.0568 | 0.0174 | 0.00504 | 0.044 | 0.0015 | -7.67E-04 | 0.0694 | 0.0530 | 0.037 | -0.0987 | -0.0459 | -0.0289 |
| 12 | 0.0568 | 0.0173 | 0.00504 | 0.044 | 0.00155 | -7.68E-04 | 0.069 | 0.0530 | 0.0372 | -0.099 | -0.046 | -0.029 |
| 13 | **0.0506** | **0.0213** | **0.0114** | **0.0305** | **-0.00232** | **-0.00309** | **0.0664** | **0.0515** | **0.0368** | **-0.0704** | **-0.0400** | **-0.0297** |
| 14 | 0.0445 | 0.0252 | 0.0178 | 0.02 | -0.00619 | -0.00541 | 0.0634 | 0.0500 | 0.037 | -0.0421 | -0.0341 | -0.031 |
| 15 | **0.0445** | **0.0252** | **0.0195** | **0.0167** | **-0.0127** | **-0.0104** | **0.0634** | **0.0500** | **0.0372** | **-0.0421** | **-0.0341** | **-0.0319** |
| 16 | **0.0445** | **0.0252** | **0.0212** | **0.0169** | **-0.0192** | **-0.0155** | **0.0634** | **0.0500** | **0.0379** | **-0.0421** | -0.0341 | **-0.0332** |
| 17 | 0.045 | 0.025 | **0.0228** | **0.0172** | **-0.0257** | **-0.0205** | 0.0634 | 0.050 | **0.0386** | -0.042 | **-0.0344** | **-0.0344** |
| 18 | 0.0535 | 0.0325 | 0.0245 | 0.017 | **-0.0323** | **-0.0255** | 0.0641 | 0.0514 | 0.0394 | **-0.0263** | -0.0346 | -0.0357 |
| 19 | 0.0722 | 0.0600 | 0.0542 | -0.00537 | -0.0388 | -0.0306 | 0.0569 | 0.0493 | 0.0436 | -0.0106 | -0.00932 | -0.0141 |
| 20 | 0.0722 | 0.0600 | **0.0542** | -0.00538 | -0.0388 | **-0.0306** | 0.0569 | 0.0493 | **0.0436** | -0.0106 | -0.00932 | **-0.0141** |
| 21 | 0.0681 | 0.0634 | **0.0542** | -0.015 | -0.0446 | **-0.0306** | 0.0536 | 0.0498 | **0.0436** | -0.00557 | -0.00489 | **-0.0141** |

|  | Neural spine length | | | Transverse process length | | | Centrum length | | | Vertebral width | | |
| --- | --- | --- | --- | --- | --- | --- | --- | --- | --- | --- | --- | --- |
| Node | Prox | Mid | Dist | Prox | Mid | Dist | Prox | Mid | Distal | Prox | Mid | Dist |
| 1 | 0.0307 | 0.0170 | 0.00312 | 0.0581 | 0.0201 | 0.00772 | 0.148 | 0.170 | 0.117 | 0.222 | 0.121 | 0.0455 |
| 2 | 0.0407 | 0.0230 | 0.0042 | 0.0494 | 0.0165 | 0.00519 | 0.144 | 0.153 | 0.118 | 0.228 | 0.120 | 0.0435 |
| 3 | 0.041 | 0.0230 | 0.0042 | 0.049 | 0.0165 | 0.00519 | 0.144 | 0.153 | 0.118 | 0.228 | 0.120 | 0.043 |
| 4 | 0.037 | 0.020 | 0.00147 | 0.050 | 0.014 | 0.00226 | 0.13 | 0.141 | 0.12 | **0.198** | 0.102 | 0.034 |
| 5 | 0.0365 | 0.0202 | 0.00147 | 0.0496 | 0.0137 | 0.00226 | 0.134 | 0.141 | 0.117 | 0.168 | 0.102 | 0.0344 |
| 6 | 0.0258 | 0.030 | 0.00280 | 0.0342 | 0.00712 | 0.00264 | 0.124 | 0.158 | 0.138 | 0.188 | 0.0891 | 0.0377 |
| 7 | **0.0258** | 0.0301 | **0.00280** | 0.0342 | 0.00711 | 0.00264 | 0.124 | 0.158 | 0.138 | **0.188** | 0.0891 | 0.0377 |
| 8 | 0.0258 | 0.0301 | 0.00279 | 0.0342 | **0.00712** | **0.00264** | 0.124 | 0.158 | 0.138 | **0.188** | **0.0891** | **0.0377** |
| 9 | 0.0258 | 0.0301 | **0.00279** | 0.0342 | 0.00712 | **0.00264** | 0.124 | 0.158 | **0.138** | **0.188** | 0.0891 | **0.0377** |
| 10 | 0.0258 | 0.0300 | 0.0028 | 0.0342 | 0.00712 | 0.00264 | 0.124 | 0.158 | 0.138 | 0.188 | 0.0891 | 0.0377 |
| 11 | 0.0258 | 0.0300 | 0.00279 | 0.034 | 0.0071 | 0.00264 | 0.124 | 0.158 | 0.138 | 0.188 | 0.0891 | 0.0377 |
| 12 | 0.0258 | 0.0300 | 0.00279 | 0.034 | 0.00711 | 0.00264 | 0.124 | 0.158 | 0.138 | **0.188** | **0.0983** | 0.038 |
| 13 | **0.0211** | **0.0218** | **0.00594** | 0.0342 | **0.00992** | **0.00796** | 0.124 | 0.158 | 0.137 | 0.188 | **0.108** | **0.0544** |
| 14 | 0.0165 | 0.0135 | 0.00908 | 0.0207 | 0.0127 | 0.0133 | 0.101 | 0.107 | 0.0984 | 0.128 | **0.117** | 0.071 |
| 15 | **0.0165** | **0.0135** | **0.00993** | 0.021 | **0.0127** | **0.0133** | 0.100 | **0.107** | **0.0983** | **0.128** | **0.126** | **0.0713** |
| 16 | 0.017 | **0.0135** | **0.0108** | 0.021 | 0.0127 | 0.0133 | 0.100 | 0.107 | 0.0982 | 0.128 | 0.135 | 0.0713 |
| 17 | 0.017 | 0.014 | **0.0116** | **0.0218** | **0.0226** | **0.0169** | 0.100 | 0.11 | **0.0911** | **0.163** | **0.172** | **0.0713** |
| 18 | 0.019 | 0.017 | 0.012 | 0.023 | **0.0324** | **0.0205** | 0.0914 | 0.0881 | 0.0840 | **0.197** | **0.208** | **0.0713** |
| 19 | 0.0287 | 0.0264 | 0.0216 | 0.0481 | 0.0422 | 0.0241 | 0.0835 | 0.0764 | 0.0727 | 0.232 | 0.244 | **0.0713** |
| 20 | 0.0287 | 0.0264 | **0.0216** | 0.0481 | 0.0422 | **0.0241** | 0.0835 | 0.0764 | **0.0727** | 0.232 | 0.245 | **0.0713** |
| 21 | 0.0302 | 0.0269 | **0.0216** | 0.0621 | 0.0568 | **0.0241** | 0.0807 | 0.0717 | **0.0727** | 0.266 | 0.277 | **0.0713** |

Table S9. Reconstructed nodal values for the vertebral parameters using SBL assumptions (interpolated values in bold font). ‘Prox’, ‘Mid’ and ‘Dist’ represent the proximal, middle and distal tail regions. Node numbers correspond to those in Figure 1.

Table S10. Tail length (size-normalised) and caudal count nodal values reconstructed using EBL and SBL assumptions.

|  | Tail length nodal values | | Caudal number nodal values | |
| --- | --- | --- | --- | --- |
| Node | EBL | SBL | EBL | SBL |
| 1 | 4.91 | 4.96 | 37 | 38 |
| 2 | 5.96 | 5.53 | 47 | 43 |
| 3 | 5.86 | 5.53 | 44 | 43 |
| 4 | 5.2 | 5.3 | 46 | 43 |
| 5 | 5.63 | 5.26 | 45 | 43 |
| 6 | 5.54 | 4.50 | 45 | 32 |
| 7 | 6.00 | 4.50 | 45 | 32 |
| 8 | 6.13 | 4.50 | 44 | 32 |
| 9 | 4.97 | 4.49 | 37 | 32 |
| 10 | 3.79 | 4.49 | 31 | 32 |
| 11 | 3.85 | 4.49 | 28 | 32 |
| 12 | 3.29 | 4.49 | 23 | 32 |
| 13 | 2.62 | 4.48 | 19 | 32 |
| 14 | 3.18 | 1.99 | 17 | 11 |
| 15 | 1.91 | 1.98 | 10 | 11 |
| 16 | 1.63 | 1.98 | 8 | 11 |
| 17 | 0.87 | 2.0 | 6 | 11 |
| 18 | 0.73 | 1.6 | 7 | 10 |
| 19 | 0.722 | 0.892 | 7 | 7 |
| 20 | 0.642 | 0.892 | 6 | 7 |
| 21 | 0.604 | 0.746 | 5 | 6 |

Table S10. Tail length (size-normalised) and caudal count nodal values using EBL and SBL assumptions. Node numbers correspond to those in Figure 1.

Table S11. Functionally informative qualitative phylogenetic tail characters (1-13).

| Char-acter | Character description |
| --- | --- |
| 1 | Number of unfused caudal vertebrae (‘free’ caudals): 44 or more (0); 43-40 (1); 39-33 (2); 32-20 (3); 19-9 (4); 8 or fewer (5) (modified from [14]). |
| 2 | Centrum articular face geometry: typical amphicoelous (0); procoelous (1); notochordal amphicoelous (2); platycoelous (3); amphiplatyan (4) or opisthocoelous (5) [15]. |
| 3 | Number of caudal vertebrae with ‘well-developed’ neural spines (dorsoventrally tall and craniocaudally short): 11 or more (0); 10 or fewer (1) (after [16]). |
| 4 | Shape of mid-caudal neural spines: dorsoventrally tall and craniocaudally short, ‘rod-like’ (0); dorsoventrally lower than craniocaudally long, ‘sub-rectangular’ and ‘sheet-like’ (1); reduced to a low ridge (2); absent (3); sagittal sulcus (4) (modified from [16]). |
| 5 | Dorsal surface of the distal caudals: low ridge (0); neural spine absent (1); sagittal sulcus (2) or neural spine present (3) (after [17,18]). |
| 6 | Last transverse process found: on caudal 23 or beyond (0); on or between caudal 22 and 8 (1) or on or before caudal 7 (2) (modified after [19]). |
| 7 | Craniocaudal length of the middle caudal prezygapophyses: short prezygapophyses that extend over half of the preceding centrum, or less (0); moderate prezygapophyses that extend over more than one half, but less than the whole length of the preceding centra (1); extremely long prezygapophyses that extend beyond the length of the preceding centra (2); or no prezygapophyses (3) (modified from [20]). |
| 8 | Craniocaudal length of the distal caudal prezygapophyses: short prezygapophyses that extend over half of the preceding centra, or less (0); moderate prezygapophyses that extend over more than one half, but less than the whole length of the preceding centra (1); extremely long prezygapophyses that extend beyond the length of the preceding centra (up to 12 centra) (2); or no prezygapophyses (3) (modified from [20]). |
| 9 | Proximal chevron shape: at least three times dorsoventrally deeper than craniocaudally short (0); less than three times dorsoventrally deeper than craniocaudally short (1); dorsoventrally flattened and craniocaudally elongated (2); or absent (3) (after [20,21,22]). |
| 10 | Middle and distal chevron shape: dorsoventrally deep and craniocaudally short (0); dorsoventrally flattened and craniocaudally elongated (1); or chevrons absent (2) (after [23,24]). |
| 11 | For middle and distal chevrons that are dorsoventrally flattened and craniocaudally elongated, these first appear on or after the caudal with the last transverse process (0); or before the caudal with the last transverse process (1) (after [23,24]). |
| 12 | Position of chevron ‘transition’ along the tail (change in chevron shape from dorsoventrally deep and craniocaudally short to dorsoventrally flattened and craniocaudally elongated): beyond caudal 17 (0); between caudal 10 and 17 (1); before caudal 10 (2); or no transition present (3) (after [20]). |
| 13 | Degree of fusion in the distal caudal vertebrae: fusion absent (0); a few vertebrae are partially ankylosed (intervening elements are discernible) (1); or vertebrae are completely fused into a pygostyle (2) [14]. |

Table S11. List of functionally informative, qualitative phylogenetic theropod tail characters (1-13) for character mapping.

Table S12 displays the qualitative phylogenetic tail data that was used to produce the results shown in Figure 8. The details of the characters listed (1-13) can be seen in Table S11 above.

Table S12. Matrix of qualitative phylogenetic tail data

| Taxon | 1 | 2 | 3 | 4 | 5 | 6 | 7 | 8 | 9 | 10 | 11 | 12 | 13 |
| --- | --- | --- | --- | --- | --- | --- | --- | --- | --- | --- | --- | --- | --- |
| *Macropus giganteus* | 3 | 0 | 1 | 2 | 0 | 2 | 0 | 0 | ? | 1 | 1 | 2 | ? |
| *Acinonyx jubatus* | 3 | 0 | 1 | 2 | 1 | 2 | 0 | 0 | 3 | 2 | - | - | 0 |
| *Varanus niloticus* | 0 | 1 | 0 | 0 | 3 | 0 | 0 | 0 | 0 | 0 | - | - | 0 |
| *Varanus komodoensis* | 0 | 1 | 0 | 0 | 3 | 0 | 0 | 0 | 1 | 0 | - | - | 0 |
| *Crocodylus niloticus* | 2 | 1 | 0 | 0 | 3 | 1 | 0 | 0 | 1 | 1 | 0 | 0 | 0 |
| *Crocodylus siamensis* | 2 | 1 | 0 | 0 | 3 | 1 | 0 | 0 | 0 | 0 | - | - | 0 |
| *Plateosaurus engelhardti* | 0 | 0 | 0 | 0 | 3 | 0 | 0 | 0 | 1 | 0 | - | 3 | 0 |
| *Dilophosaurus wetherilli* | 0 | 0 | 0 | 0 | 0 | 1 | 0 | 0 | 1 | ? | ? | ? | ? |
| *Coelophysis bauri* | 2 | ? | 0 | 2 | ? | 1 | 0 | 0 | ? | 1 | ? | ? | 0 |
| *Tyrannosaurus rex* | 0 | 0 | 0 | 1 | 0 | 1 | 0 | 1 | 0 | 1 | 0 | 1 | 0 |
| *Ornitholestes hermanni* | ? | 0 | ? | 2 | ? | 1 | 0 | ? | ? | 1 | ? | ? | ? |
| *Compsognathus longipes* | ? | 3 | 0 | ? | ? | 1 | 0 | ? | 1 | 1 | 1 | 1 | ? |
| *Juravenator starki* | 0 | ? | 0 | ? | ? | ? | 0 | 0 | 0 | 0 | ? | 0 | ? |
| *Shuvuuia deserti* | 2 | 1 | 1 | 2 | 0 | 1 | 0 | 0 | ? | 1 | 1 | 2 | 0 |
| *Caudipteryx zoui* | 3 | ? | 0 | ? | ? | 1 | 0 | 0 | 0 | 1 | ? | 1 | 0 |
| *Khaan mckennai* | 3 | 4 | 0 | 1 | 0 | 1 | 0 | 0 | 0 | ? | ? | ? | 0 |
| *Citipati osmolskae* | 3 | 4 | 0 | 1 | 0 | 0 | 0 | 0 | 1 | 1 | 1 | 1 | 1 |
| *Mei long* | 3 | 0 | 1 | 4 | 2 | 1 | 0 | 0 | ? | 1 | ? | 2 | 0 |
| *Sinusonasus magnodens* | 3 | ? | 1 | 4 | 2 | 2 | 0 | 0 | 1 | 1 | 1 | 2 | 0 |
| *Sinornithoides youngi* | 3 | ? | ? | ? | ? | 1 | 0 | 0 | 0 | 1 | 1 | 2 | 0 |
| *Mahakala osmolskae* | ? | 0 | 1 | 2 | ? | 1 | 0 | 0 | ? | 1 | 1 | 2 | ? |
| *Velociraptor mongoliensis -* MPC 100/985 | ? | 0 | 0 | 2 | 0 | 1 | 2 | 2 | 0 | 1 | 1 | 1 | 0 |
| *Velociraptor mongoliensis -* fighting specimen | 3 | 0 | 0 | ? | ? | 1 | 2 | 2 | 0 | 1 | 1 | 2 | 0 |
| *Deinonychus antirrhopus* | 1 | 0 | 0 | 2 | 1 | 1 | 2 | 2 | 0 | 1 | 1 | 2 | ? |
| *Bambiraptor feinbergi* | ? | 0 | ? | ? | 1 | 2 | 2 | 2 | 0 | 1 | 0 | 2 | ? |
| Undescribed Liaoning dromaeosaurid | 3 | ? | ? | ? | ? | ? | 2 | 2 | ? | 1 | ? | ? | 0 |
| *Microraptor zhaoianus* | 3 | ? | ? | ? | ? | 2 | 2 | 2 | ? | 1 | 0 | 2 | 0 |
| *Microraptor gui* | 3 | ? | ? | ? | ? | ? | 2 | 2 | ? | 1 | ? | ? | 0 |
| *Archaeopteryx lithographica –* Munich | 3 | ? | 1 | ? | ? | 2 | 0 | 0 | 0 | 1 | ? | 2 | 0 |
| *Archaeopteryx lithographica –* Eichstätt | 3 | ? | 1 | 3 | 1 | 2 | 0 | 0 | 1 | 1 | 1 | 2 | 0 |
| *Archaeopteryx lithographica –* Berlin | 3 | ? | 1 | 3 | 1 | 1 | 0 | 0 | 1 | 1 | 1 | 2 | 0 |
| *Epidexipteryx hui* | 4 | ? | ? | ? | ? | 1 | ? | ? | ? | ? | ? | ? | 0 |
| *Jeholornis prima* | 3 | ? | 1 | ? | ? | 2 | 0 | 0 | 1 | 1 | 1 | 2 | 0 |
| *Confuciusornis sanctus* | 5 | 0 | 1 | 0 | 3 | 2 | 0 | 0 | ? | ? | ? | ? | 1 |
| *Sapeornis chaoyangensis* | 4 | 0 | 1 | ? | 0 | 1 | 0 | 0 | ? | 1 | 1 | 2 | 1 |
| *Hongshanornis longicresta* | ? | ? | ? | ? | 3 | ? | ? | 0 | ? | ? | ? | ? | 1 |
| *Yixianornis grabaui* | 5 | 0 | 1 | 0 | 3 | ? | 0 | 0 | ? | 1 | ? | 2 | 1 |
| *Struthio camelus* | 4 | 0 | 1 | 0 | 3 | 1 | 3 | 3 | 3 | 2 | - | - | 1 |
| *Columba livia* | 5 | 1 | 1 | 0 | 3 | 2 | 0 | 0 | 3 | 2 | - | - | 2 |
| *Meleagris gallopavo* | 5 | 1 | 1 | 0 | 3 | 2 | 0 | 0 | 3 | 2 | - | - | 2 |
| *Pavo muticus* | 5 | 1 | 1 | 0 | 3 | 2 | 0 | 0 | 3 | 2 | - | - | 2 |

Table S12. Matrix of functionally informative, qualitative phylogenetic theropod tail data. ‘?’ denotes missing data, whilst ‘-’ is a character that is not applicable to the taxon coded.

The character list (1-13) is given in Table S11.

References

1. Brochu CA (2003) Osteology of *Tyrannosaurus rex*: insights from a nearly complete skeleton and high-resolution computed tomographic analysis of the skull. Society of Vertebrate Paleontology Memoir 7: 1-138.

2. Hwang SH, Norell MA, Ji Q, Gao KQ (2002) New specimens of *Microraptor zhaoianus* (Theropoda: Dromaeosauridae) from Northeastern China. American Museum Novitates 3381: 1-44.

3. Zhou ZH, Zhang FC (2002) A long-tailed, seed-eating bird from the Early Cretaceous of China. Nature 418: 405-409.

4. Ezcurra MD, Brusatte SL (2011) Taxonomic and phylogenetic reassessment of the early neotheropod dinosaur *Camposaurus arizonensis* from the Late Triassic of North America. Palaeontology 54: 763-772.

5. Smith ND, Makovicky PJ, Hammer WR, Currie PJ (2007) Osteology of *Cryolophosaurus ellioti* (Dinosauria: Theropoda) from the Early Jurassic of Antarctica and implications for early theropod evolution. Zoological Journal of the Linnean Society 151: 377-421.

6. Yates AM (2006) A new theropod dinosaur from the Early Jurassic of South Africa and its implications for the early evolution of theropods. Palaeontologia Africana 41: 105-122.

7. Currie PJ, Chen PJ (2001) Anatomy of *Sinosauropteryx prima* from Liaoning, northeastern China. Canadian Journal of Earth Sciences 38: 1705-1727.

8. Osborn HF (1916) Skeletal adaptations of *Ornitholestes*, *Struthiomimus*, *Tyrannosaurus*. Bulletin of the American Museum of Natural History 35: 733-771.

9. Suzuki S, Chiappe LM, Dyke GJ, Watabe M, Barsbold R, et al. (2002) A new specimen of *Shuvuuia deserti* Chiappe *et al*., 1998 from the Mongolian Late Cretaceous with a discussion of the relationships of alvarezsaurids to other theropod dinosaurs. Contributions in Science, Natural History Museum of Los Angeles County 494: 1-18.

10. Xu X, Wang XL (2004) A new troodontid (Theropoda: Troodontidae) from the Lower Cretaceous Yixian Formation of western Liaoning, China. Acta Geologica Sinica 78: 22-26.

11. Norell MA, Makovicky PJ (2004) Dromaeosauridae. In: Weishampel DB, Dodson P, Osmólska H, editors. The Dinosauria. Berkeley: University of California Press. pp. 196-209.

12. Xu X, Zhou ZH, Wang XL, Kuang X, Zhang F, et al. (2003) Four-winged dinosaurs from China. Nature 421: 335-340.

13. Galton PM, Upchurch P (2004) Prosauropoda. In: Weishampel DB, Dodson P, Osmólska H, editors. The Dinosauria. 2nd ed. Berkeley: University of California Press. pp. 232-258.

14. Gao CL, Chiappe LM, Meng QJ, O'Connor JM, Wang XR, et al. (2008) A new basal lineage of early Cretaceous birds from China and its implications on the evolution of the avian tail. Palaeontology 51: 775-791.

15. Romer AS (1956) Osteology of the reptiles. Chicago: University of Chicago. 772 p.

16. Rauhut OWM (2003) The interrelationships and evolution of basal theropod dinosaurs. Special Papers in Palaeontology. London: The Palaeontological Association. pp. 213.

17. Osmólska H, Currie PJ, Barsbold R (2004) Oviraptorosauria. In: Weishampel DB, Dodson P, Osmólska H, editors. The Dinosauria 2ed. Berkeley: University of California Press. pp. 165-183.

18. Russell DA, Dong, Z.M. (1993) A nearly complete skeleton of a new troodontid dinosaur from the Early Cretaceous of the Ordos Basin, Inner Mongolia, People's Republic of China. Canadian Journal of Earth Sciences 30: 2163-2173

19. Makovicky PJ, Apesteguía, S., Agnolin, F.L. (2005) The earliest dromaeosaurid theropod from South America. Nature 437: 1007-1011.

20. Gauthier JA (1986) Saurischian monophyly and the origin of birds. In: Padian K, editor. The origin of birds and the evolution of flight. San Francisco: Memoirs of the California Academy of Science. pp. 1-55.

21. Chiappe LM (2002) Early bird phylogeny: problems & solutions. In: Chiappe LM, Witmer L, editors. Mesozoic birds: above the heads of dinosaurs. Berkeley: University of California Press. pp. 448-472.

22. Makovicky PJ, Sues, H.D. (1998) Anatomy and phylogenetic relationships of the theropod dinosaur *Microvenator celer* from the Lower Cretaceous of Montana. American Museum Novitates 3240: 1-27.

23. Sereno PC, Wilson, J.A., Larsson, H.C.E., Dutheil, D.B., Sues, H.D. (1994) Early Cretaceous dinosaurs from the Sahara. Science 266: 267-270.

24. Sereno PC, Dutheil DB, Larochene M, Larsson HCE, Lyon GH, et al. (1996) Predatory dinosaurs from the Sahara and Late Cretaceous faunal differentiation. Science 272: 986-991.
