# Supplementary material for: Shake a Tail Feather: The Evolution of the Theropod Tail into a Stiff Aerodynamic Surface
Source: PLoS One. 2013 May 15;8(5):e63115. doi: 10.1371/journal.pone.0063115 (PMC3655181; doi:10.1371/journal.pone.0063115)
Supplement: File S3 — Supporting Information Figure showing the correlations between size-normalised tail and centrum length nodal values reconstructed for amniotes. Figure S1. Correlations between size-normalised amniote tail and centrum length nodal values: A, tail length shows a strong linear correlation with proximal centrum length (EBL data: y = 59.164x−3.5387, R2 = 0.8551, r = 0.925 which is significant at the 0.01 level (p (2-tailed) = 0.000); SBL data: y = 79.919x−5.716, R2 = 0.9504, r = 0.975 which is significant at the 0.01 level (p (2-tailed) = 0.000)), B, tail length also shows a strong linear correlation with middle centrum length (EBL data: y = 38.284x−1.3233, R2 = 0.6211, r = 0.788 which is significant at the 0.01 level (p (2-tailed) = 0.000); SBL data, y = 48.134x - 2.7588, R2 = 0.8856, r = 0.941 which is significant at the 0.01 level (p (2-tailed) = 0.000)), C, tail length shows a weak linear correlation with distal centrum length (EBL data: y = 44.356x−0.9222, R2 = 0.4356, r = 0.660 which is significant at the 0.01 level (p (2-tailed) = 0.005); SBL data: y = 54.93x−2.5022, R2 = 0.5878, r = 0.767 which is significant at the 0.01 level (p (2-tailed) = 0.001)). Node numbers (1–21, Fig. 1) are marked next to each EBL and SBL data point. (DOCX) [file pone.0063115.s003.docx]

File S3. Supporting Information Figure

Shake a tail feather: the evolution of the theropod tail into a stiff aerodynamic surface

Michael Pittman^1^*, Stephen M. Gatesy^2^, Paul Upchurch^1^, Anjali Goswami^1,3^, John R. Hutchinson^4^

^1^Department of Earth Sciences, University College London, Gower Street, London, WC1E 6BT, U.K.

^2^Department of Ecology and Evolutionary Biology, Brown University, Providence, RI, 02912, U.S.A.

^3^Department of Genetics, Evolution, and Environment, University College London, Wolfson House, Stephenson Way, London, NW1 2HE, U.K.

^4^Structure & Motion Laboratory, Department of Comparative Biomedical Sciences, The Royal Veterinary College, Hawkshead Lane, Hatfield, AL9 7TA, U.K.

*mpittman@hku.hk

Current address: Department of Earth Sciences, The University of Hong Kong, Pokfulam, Hong Kong.

One figure (Figure S1) comprises File S3.

Figure S1. Correlations between size-normalised tail and centrum length nodal values reconstructed for amniotes


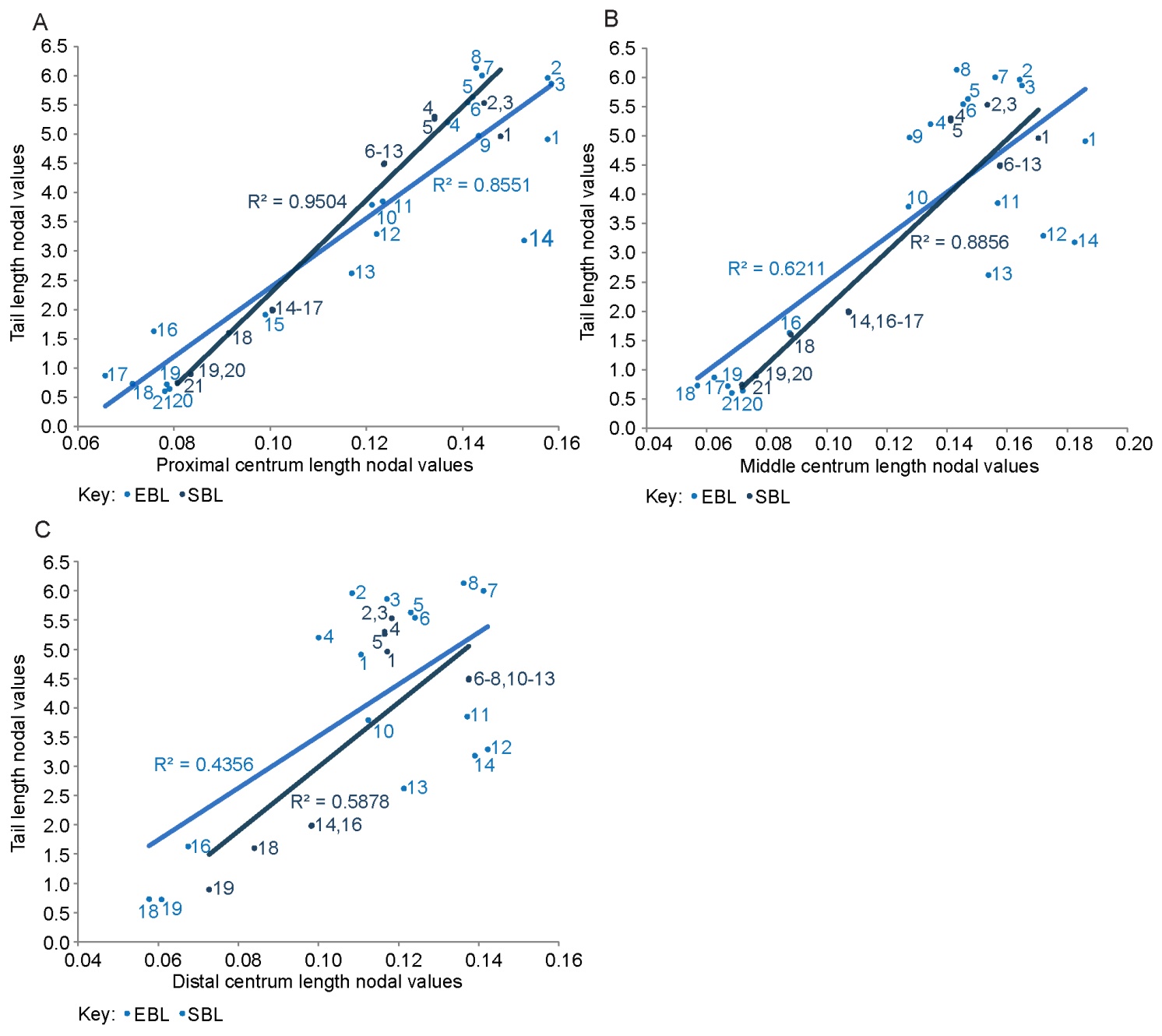


Figure S1. Correlations between size-normalised amniote tail and centrum length nodal values: A, tail length shows a strong linear correlation with proximal centrum length (EBL data: y = 59.164x - 3.5387, R² = 0.8551, *r* = 0.925 which is significant at the 0.01 level (*p* (2-tailed) *=* 0.000); SBL data: y = 79.919x - 5.716, R² = 0.9504, *r* = 0.975 which is significant at the 0.01 level (*p* (2-tailed) *=* 0.000)), B, tail length also shows a strong linear correlation with middle centrum length (EBL data: y = 38.284x - 1.3233, R² = 0.6211, *r* = 0.788 which is significant at the 0.01 level (*p* (2-tailed) *=* 0.000); SBL data, y = 48.134x - 2.7588, R² = 0.8856, *r* = 0.941 which is significant at the 0.01 level (*p* (2-tailed) *=* 0.000)), C, tail length shows a weak linear correlation with distal centrum length (EBL data: y = 44.356x - 0.9222, R² = 0.4356, *r* = 0.660 which is significant at the 0.01 level (*p* (2-tailed) *=* 0.005); SBL data: y = 54.93x - 2.5022, R² = 0.5878, *r* = 0.767 which is significant at the 0.01 level (*p* (2-tailed) *=* 0.001)). Node numbers (1-21, Fig. 1) are marked next to each EBL and SBL data point.
